# LAP2α preserves genome integrity through assisting RPA deposition on damaged chromatin

**DOI:** 10.1186/s13059-022-02638-6

**Published:** 2022-02-28

**Authors:** Kaiwen Bao, Qi Zhang, Shuai Liu, Nan Song, Qiushi Guo, Ling Liu, Shanshan Tian, Jihui Hao, Yi Zhu, Kai Zhang, Ding Ai, Jie Yang, Zhi Yao, Roland Foisner, Lei Shi

**Affiliations:** 1 State Key Laboratory of Experimental Hematology, The Province and Ministry Co-sponsored Collaborative Innovation Center for Medical Epigenetics, Key Laboratory of Immune Microenvironment and Disease (Ministry of Education), Key Laboratory of Breast Cancer Prevention and Therapy (Ministry of Education), Tianjin Medical University Cancer Institute and Hospital, Tianjin Medical University General Hospital, School of Basic Medical Sciences, Tianjin Medical University, Tianjin, 300070 China; 2 Department of Clinical Laboratory, First Affiliated Hospital of Xi’an Jiaotong University, Xi’an, 710061 Shaanxi China; 3 Max Perutz Laboratories, Center of Medical Biochemistry, Medical University of Vienna, Vienna Biocenter (VBC), Vienna, Austria; 4 Department of Biochemistry and Molecular Biology, Tianjin Medical University, 22 Qixiangtai Road, Tianjin, 300070 China

**Keywords:** RPA loading, Genome stability, ATR activation, Homologous recombination, LAP2α, PARP1

## Abstract

**Background:**

Single-stranded DNA (ssDNA) coated with replication protein A (RPA) acts as a key platform for the recruitment and exchange of genome maintenance factors in DNA damage response. Yet, how the formation of the ssDNA-RPA intermediate is regulated remains elusive.

**Results:**

Here, we report that the lamin-associated protein LAP2α is physically associated with RPA, and LAP2α preferentially facilitates RPA deposition on damaged chromatin via physical contacts between LAP2α and RPA1. Importantly, LAP2α-promoted RPA binding to ssDNA plays a critical role in protection of replication forks, activation of ATR, and repair of damaged DNA. We further demonstrate that the preference of LAP2α-promoted RPA loading on damaged chromatin depends on poly ADP-ribose polymerase PARP1, but not poly(ADP-ribosyl)ation.

**Conclusions:**

Our study provides mechanistic insight into RPA deposition in response to DNA damage and reveals a genome protection role of LAP2α.

**Supplementary Information:**

The online version contains supplementary material available at 10.1186/s13059-022-02638-6.

## Background

During each cell cycle of eukaryotic cells, genetic inheritance is constantly challenged by endogenous or exogenous assaults that can undermine the integrity of the replicating genome [1–3]. In physiological settings, mild forms of replication stress stochastically occur during cell-cycle progression, while the severe forms could be further induced in pathological states such as oncogenic transformation and chemotherapeutic agents, which could lead to DNA damage and instigate genomic instability [4–6]. There are numerous impediments that block the progression of DNA replication forks, including DNA lesions, the transcription machinery, DNA-protein complex, RNA-DNA hybrids, and secondary DNA structures [7–10]. If replication stress persists, stalled forks could be converted into collapsed forks and even DNA double-strand breaks (DSBs) that pose the most serious threat to genome integrity [10]. Although these complexed structures may differ greatly, they all result in the formation of long stretches of single-stranded DNA (ssDNA) that are coated by replication protein A (RPA), a common intermediate product, which, in turn, provides a platform for the recruitment and exchange of genome maintenance factors to respond to stresses or damages and orchestrates a genome surveillance pathway [11–13].

As one of the early phase responders in the replication stress, ssDNA-RPA is the key structure that triggers the activation of serine/threonine kinase Ataxia telangiectasia and Rad3 related (ATR), a master regulator of the DNA damage response (DDR) [3, 11]. Once activated, ATR coordinates with its downstream effectors to counteract adverse effects of replication stress and repair of damaged DNA both by delaying cell-cycle progression and by stabilizing stalled forks [14, 15]. In support of the notion that RPA-ATR signalling axis is essential for the maintenance of genome integrity, disruption of RPA in mice results in defective repair of DSBs, chromosomal instability, and tumorigenesis [16, 17], and defects of ATR signalling leads to chromosome fragmentation, developmental failures, accelerated aging, and predisposition of carcinogenesis [18–21]. When DSBs occur, RPA sequesters the ssDNA of the resected DSB, allowing the formation of the RAD51-ssDNA nucleoprotein filament to catalyze strand invasion for accurate repair of DSBs by homologous recombination (HR) [22]. These evidences pinpoint the importance of RPA-dependent surveillance network in genome stability.

RPA is composed of heterotrimeric subunits RPA1, RPA2, and RPA3, and it binds to ssDNA with sub-nanomolar affinity and protects it against nucleolytic degradation or breakage as well as removes secondary structures [13, 23]. As a stable, flexible, and modular protein complex, RPA contains six oligosaccharide/oligonucleotide binding (OB) folds that are responsible for the association of RPA with a short or long stretch of ssDNA [13, 23]. The largest subunit, RPA1, possesses four OB domains commonly referred to as DNA-binding domains (DBDs): DBD-A, DBD-B, DBD-C, and DBD-F, which are connected by flexible linkers [24–26]. RPA2 has a single OB-fold (DBD-D) to constitute the trimerisation core (Tri-C) with DBD-C of RPA1 and DBD-E of RPA3 [24, 25]. Structural, biochemical, and biophysical studies have shown that each individual DBD from A to E possesses intrinsic ssDNA binding activity, albeit with different affinity [24, 25, 27, 28], and the combinatorial action of these domains enables RPA binding to ssDNA with a defined polarity from 5′ to 3′ using two different modes of binding [24, 25, 27, 29]: the 8 to 10 nucleotides (nt) occluded only by its DBD-A and BDD-B uses an extended conformation with lower affinity, whereas the 28- to 30-nt binding by fully engaged RPA goes along with a compact protein structure and higher affinity. These two different binding modalities appear to act sequentially to facilitate the association of RPA with ssDNA.

A large number of RPA or ssDNA-RPA interaction proteins or protein complexes have been characterized, and ssDNA-RPA complex is believed to act as a key platform to coordinate the arrival and departure of these factors whose combined activities permit the protection of eukaryotic genomes against damages or stresses [13]. Interestingly, several studies have reported that RPA binding to ssDNA could be regulated by defined factors such as histone methyltransferase G9a [30], DNA helicase Fanconi anemia group J protein (FANCJ) [31], and tumor suppressor protein phosphatase and tensin homolog (PTEN) [17]. Relevantly, cell division cycle protein 45 (CDC45), a component of the active replicative helicase CDC45/MCM2-7/GINS (CMG), has been proposed to directly load RPA on the emerging nascent ssDNA at replication fork [32], and protein regulator of Ty1 transposition 105 (Rtt105) from yeast has been shown to function as a canonical RPA chaperone that helps deposit RPA at stalled replication forks [33]. These two studies point that RPA could be actively loaded onto ssDNA by chaperone or chaperone-like proteins. Yet, it is still not fully understood, in mammalian cells, how the formation of ssDNA-RPA intermediate generated during replication stress or at DNA lesions is regulated.

Lamin-associated polypeptide 2 alpha (LAP2α) is the largest splicing product of the LAP2 gene (*TMPO*) that encodes six variants sharing a constant N-terminal LAP2, emerin, and MAN1 (LEM) like domain linked through a flexible tether to the LEM domain [34, 35]. Unlike other LAP2 isoforms that are preferentially anchored to the nuclear inner membrane through a transmembrane domain and binding to lamins, the LAP2α isoform replaces the transmembrane domain with a unique coiled-coil domain in the C-terminal region and localizes throughout the nucleus [34]. This distinct C-terminal region interacts with A-type lamins and defines Lamin A/C localization in the nucleoplasm [36], which in turn affects retinoblastoma protein (pRb)-mediated regulation of gene expression, and progenitor cell proliferation and differentiation in highly regenerative tissues [37]. However, the activity of LAP2α in other fundamental cellular processes including DDR and genome stability, dysregulation of which will contribute to tumorigenesis, has not been well documented.

Here, we report that LAP2α functions to assist RPA deposition and maintain genome stability in mammalian cells. Specifically, we revealed that LAP2α preserves genome integrity through controlling the formation of ssDNA-RPA complex on damaged chromatin, and LAP2α-promoted RPA loading is required for fork stability, ATR activation, and HR repair of DSBs.

## Results

### RPA is physically associated with LAP2α

**Fig. 1:**
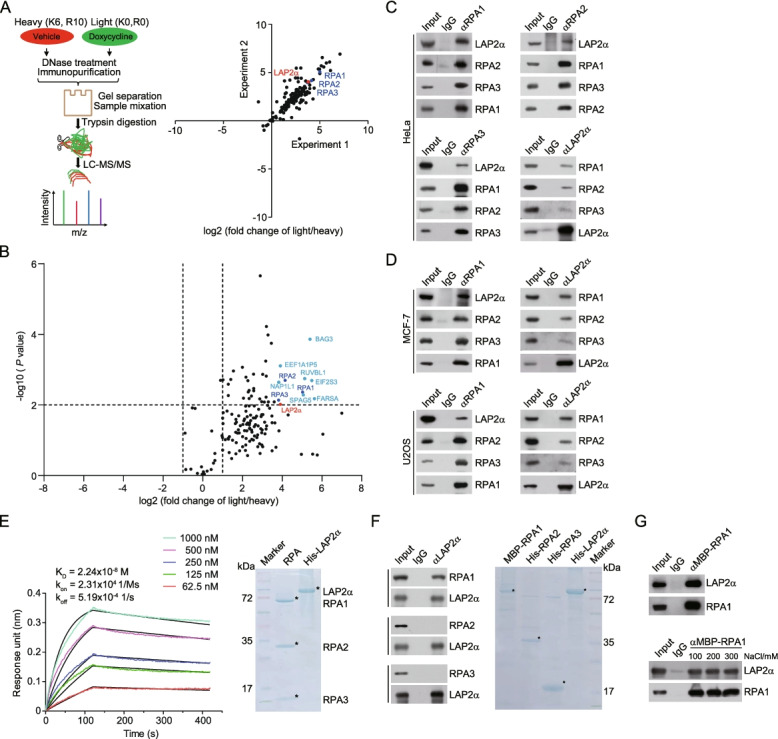
RPA is physically associated with LAP2α. **A** SILAC-based quantitative mass spectrometry analysis of RPA1-containing protein complexes with HeLa cells that allow doxycycline (Dox)-inducible expression of stably integrated FLAG-RPA1. Control cells were labelled with “heavy isotopic lysine and arginine” (K6R10) and the cells under Dox (1 ng/μl) treatment were labelled with “light isotopic lysine and arginine” (K0R0). DNase (300 U/ml)-treated cellular extracts were immunopurified with anti-FLAG affinity beads and eluted with FLAG peptide. The eluates were desalted by gel separation and mixed for digestion followed by mass spectrometry analysis. Biological duplicate experiments were performed. **B** Volcano plot showing the relative enrichment of RPA1-containing protein complex from SILAC-based quantitative mass spectrometry. Each point represents the potential interactor, and the 11 top candidates (fold change > 15 and *P* value < 0.01) are indicated. **C** Co-immunoprecipitation analysis of the interaction between LAP2α and RPA. Whole-cell lysates from HeLa cells were pre-treated with DNase followed by immunoprecipitation and immunoblotting with antibodies against the indicated proteins. α, anti-. **D** Co-immunoprecipitation analysis of the interaction between LAP2α and RPA. Whole-cell lysates from MCF-7 and U2OS cells were pre-treated with DNase followed by immunoprecipitation and immunoblotting with antibodies against the indicated proteins. **E** Analysis of the binding affinity of recombinant RPA purified from bacteria cells and His-tagged LAP2α purified from insect cells by biolayer interferometry (BLI) assay. The black line indicates fitted curves and the color traces represent raw data. Data are representative of two independent experiments. RPA1, RPA2, and RPA3 of the RPA complex were co-purified and examined by Coomassie brilliant blue staining. **F** Co-immunoprecipitation analysis of the association of individual recombinant RPA subunit with LAP2α. All proteins were individually purified from insect cells. **G** Co-immunoprecipitation analysis of the association of recombinant RPA1 with LAP2α in buffer with an increasing amount of ionic strength

To identify potential regulators that control RPA loading onto ssDNA, we employed immunopurification and mass spectrometry with stable isotope labelling with amino acids in cell culture (SILAC) to interrogate free RPA interactome. Quantitative mass spectrometry analysis of FLAG-RPA1 containing protein complex in the presence of DNase revealed that RPA1 was associated with a number of proteins, including RPA2 and RPA3 (Fig. 1A, B, and Additional file 1: Table S1). Interestingly, LAP2α, a previously characterized Lamin A/C and pRB-associated nuclear protein, was identified as one of the top candidates (Fig. 1A, B, and Additional file 1: Table S1). Since the enrichment of LAP2α was almost comparable to that of each RPA subunit (Fig. 1A, B, and Additional file 1: Table S1), we focused on further characterization of LAP2α in RPA binding and regulation. To confirm the association of LAP2α with RPA, co-immunoprecipitation experiments were then performed and the results showed that LAP2α was efficiently co-immunoprecipitated with RPA, and vice versa under DNase treatment (Fig. 1C,D).

To validate the interaction between LAP2α and RPA and to gain a molecular insight into the assembly of the LAP2α/RPA complex, real-time binding of LAP2α to RPA was monitored by biolayer interferometry (BLI) with recombinant proteins. After immobilizing RPA on the surface of a sensor chip, various concentrations of recombinant LAP2α were injected over the immobilized RPA to measure the binding affinities. This quantitative biophysical analysis shown as representative sensorgrams with global fitting curves revealed that LAP2α strongly binds to RPA with an apparent dissociation constant (*K*_D_) of 22.4 nM (Fig. 1E). Next, we examined the binding of LAP2α to each recombinant component of RPA with pull-down assays. The results indicated that only RPA1 could directly and strongly interact with LAP2α (Fig. 1F, G). Collectively, these results suggest that LAP2α is physically associated with RPA through directly binding to RPA1.

### Key determinants for LAP2α-RPA binding

**Fig. 2:**
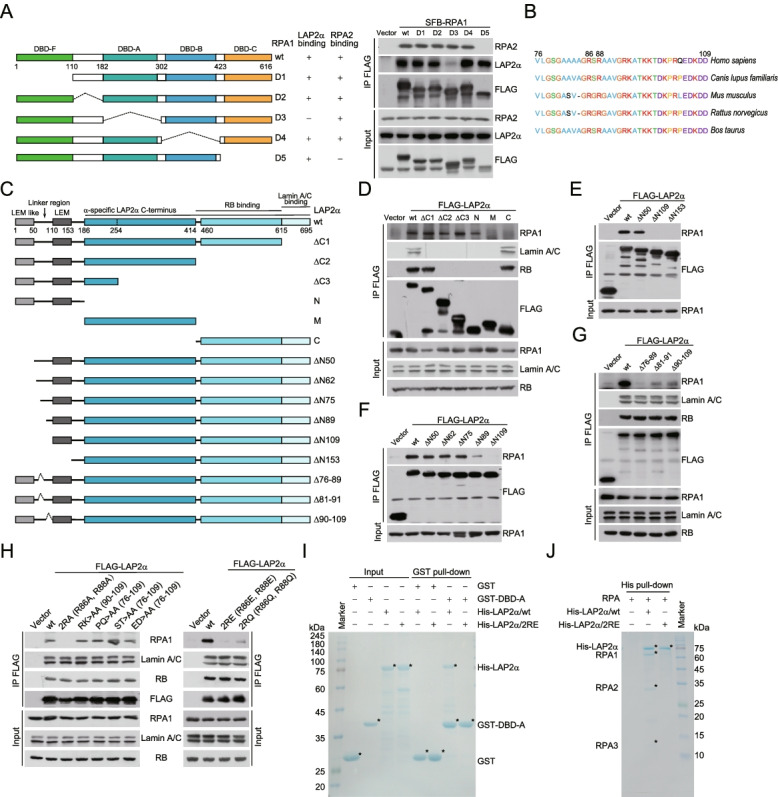
Key determinants for LAP2α-RPA binding. **A** Whole-cell lysates from HeLa cells expressing S protein-FLAG-Streptavidin binding peptide (SFB)-tagged RPA1 deletions were immunoprecipitated and immunoblotted with antibodies against the indicated proteins. The RPA1 domain and deletion mutants annotated with residue numbers are shown. DBD represents DNA-binding domain. **B** Schematic representation of the evolutionarily conserved residues in the linker region of LAP2α. **C** Schematic representation of the domain structure of LAP2α and its variants. The domains and truncated or deleted variants are marked with the indicated residue numbers. The LEM (LAP2, emerin, MAN1) domain, LEM-like domain, LAP2α-specific C-terminus, and RB or Lamin A/C binding regions are shown. N, N-terminus from amino acid 1 to 185; M, middle region from 186 to 414; C, C-terminus from 415-695. ΔN50, residues from 1-50 are deleted; Δ76-89, residues from 76 to 89 are deleted; and so forth. **D–F** Whole-cell lysates from HeLa cells expressing FLAG-GFP-tagged LAP2α truncation mutants were immunoprecipitated and immunoblotted with antibodies against the indicated proteins. **G** Whole-cell lysates from HeLa cells expressing FLAG-GFP-tagged LAP2α deletion mutants were immunoprecipitated and immunoblotted with antibodies against the indicated proteins. **H** FLAG-tagged LAP2α variants were transfected into HeLa cells followed by co-immunoprecipitation and immunoblotting analysis. RK>AA (90-109) represents that all arginine and lysine residues from amino acid 90 to 109 of LAP2α are simultaneously replaced by alanine, and so forth. **I** GST pull-down assays with recombinant GST-DBD-A and His-tagged LAP2α variants. GST-DBD-A and His-tagged LAP2α were purified from bacteria cells and insect cells, respectively. **J** His pull-down assays with recombinant His-tagged LAP2α variants and RPA. RPA (including RPA1, RPA2, and RPA3) and His-tagged LAP2α variants were purified from bacteria cells and insect cells, respectively. The asterisks indicate the recombinant proteins stained by Coomassie brilliant blue

To understand the molecular details of the association of LAP2α with RPA1, domain deletion mutants of RPA1 were generated. Co-immunoprecipitation experiments with RPA1 mutants followed by immunoblotting with RPA2 and LAP2α revealed that the DBD-A domain of RPA1 was specifically required for the interaction of RPA1 with LAP2α, while the DBD-C domain deletion variant that could not bind RPA2 was still able to interact with LAP2α (Fig. 2A). These results further confirmed that RPA1 functions to directly engage LAP2α into the RPA complex. Reciprocally, co-immunoprecipitation assays indicated that the linker region, which is conserved across different species (Fig. 2B, C), was responsible for the interaction of LAP2α with RPA1 (Fig. 2D, E), and the C-terminal region of LAP2α was responsible for its binding to Lamin A/C and pRB as previously reported [36] (Fig. 2D). Further chopping of the linker region of LAP2α showed that the region spanning from amino acid 76 to 89 (76–89) was required for the interaction of LAP2α with RPA1 (Fig. 2F), and co-immunoprecipitation assays with deletion mutants suggested that the fragment 90–109 was also essential in LAP2α-RPA1 binding (Fig. 2G).

We then applied site-directed mutagenesis and measured the effect of these mutations on the interaction between LAP2α and RPA1. The results indicated that Arg 86 (R86) and Arg 88 (R88) were critically required for LAP2α-RPA1 binding, as alanine (LAP2α/2RA), glutamate (LAP2α/2RE), or glutamine (LAP2α/2RQ) substitution dramatically compromised the interaction (Fig. 2H). Meanwhile, we found these variants had minor effect on the association of LAP2α with Lamin A/C or pRB (Fig. 2H). Since all of the mutations among 90–109 had little effect on LAP2α-RPA1 partnering (Fig. 2H), we propose that 76–89 in the linker region of LAP2α likely binds to RPA1 directly, while 90–109 may contribute to the interaction indirectly. Moreover, pull-down experiments with recombinant proteins demonstrated that wild type LAP2α (LAP2α/wt), but not LAP2α/2RE, could bind DBD-A or RPA (Fig. 2I, J). These results suggest R86/R88 act as key determinants for the interaction of LAP2α with RPA.

### LAP2α promotes the loading of RPA onto damaged chromatin

**Fig. 3:**
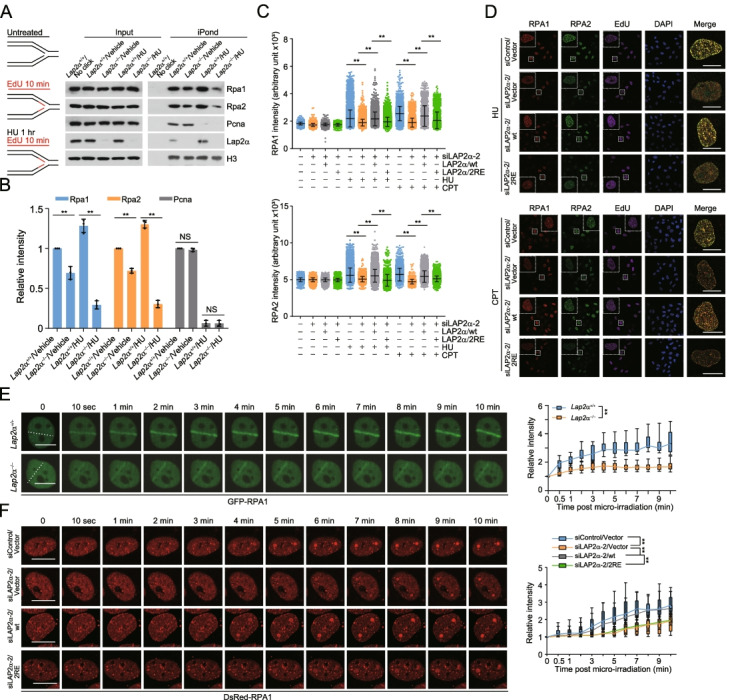
LAP2α promotes the loading of RPA onto damaged chromatin. **A** Proteins associated with replication forks were isolated by iPOND as described in “Methods” and detected by immunoblotting. *Lap2α*^*+*/*+*^ or *Lap2α*^*−*/*−*^ MEFs were EdU-labelled for 10 min and harvested immediately or after a 1-h chase with 2 mM HU. **B** Quantitative analysis of Rpa1, Rpa2, and Pcna binding to EdU-labelled nascent replication forks with iPOND assays. The relative intensity of chromatin bound factors to histone H3 was normalized against that in vehicle-treated control cells. **C** High content imaging analysis of foci intensity of RPA1 and RPA2 in U2OS cells. pLenti-vector, LAP2α/wt or LAP2α/2RE stably integrated U2OS cells were transfected with control or LAP2α 3′UTR siRNA (siLAP2α-2) and treated with 2 mM HU for 4 h or 1 μM CPT for 2 h followed by pre-extraction, fixation, immunostaining, and high content microscopy analysis. Before collection, cells were labelled with EdU for 1 h. **D** Representative images from **C** collected by confocal microscopy. **E** Laser micro-irradiation (IR) (50% laser energy) followed by live cell imaging analysis of GFP-RPA1 recruitment kinetics in GFP-RPA1 expressing *Lap2α*^*+*/*+*^ or *Lap2α*^*−*/*−*^ MEFs. Fluorescence intensities in micro-irradiated areas relative to the nuclear background were quantified (*n* > 20). **F** pLenti-vector, LAP2α/wt or LAP2α/2RE stably integrated U2OS cells were co-transfected with DsRed-RPA1 and control siRNA or LAP2α 3′UTR siRNA followed by Hoechst sensitization, micro-IR (405 nm) and live cell imaging analysis. Fluorescence intensities in micro-irradiated areas relative to the nuclear background were quantified (*n* > 20). Data are mean ± SDs for **B**, **E**, and **F** from biological triplicate experiments, and **C** from biological duplicate experiments. **P < 0.01; NS, not significant; one-way ANOVA for **B**; Mann-Whitney test for **C**; two-way ANOVA for **E** and **F**. Scale bar, 10 μm

Since the ssDNA-RPA platform constitutes a key physiological signal in orchestrating DNA replication and repair, we wondered whether LAP2α could facilitate RPA deposition onto ssDNA in vivo. First, isolation of proteins on nascent DNA (iPOND) assay was performed to analyze chromatin factors bound to nascent replicating forks in the absence or presence of hydroxyurea (HU), a reversible inhibitor of ribonucleotide reductase that stalls DNA replication forks by reducing cellular deoxynucleotide pools [38]. The results revealed that, in *Lap2α* knockout (*Lap2α* ^*−*/*−*^) mouse embryonic fibroblast cells (MEFs), RPA loading at EdU-labelled nascent ssDNA of replication forks was dramatically disrupted under HU treatment (Fig. 3A, B). We showed that *Lap2α* knockout or LAP2α knockdown only had a moderate effect on RPA deposition in the absence of HU treatment (Fig. 3A, B and Additional file 2: Figure S1A), suggesting LAP2α may play a limited role in RPA loading when DNA replicates. By contrast, the iPOND signal of RPA was markedly reduced in cells expressing siRNA against CDC45 (Additional file 2: Figure S1A), which has been proposed to directly load RPA on the emerging nascent ssDNA at replication fork [32]. The binding of DNA sliding clamp proliferating nuclear antigen A (PCNA), a processivity factor for the elongation of nascent DNA strands at replication forks, was examined to monitor replication fork activity and it was largely unaffected in *Lap2*α ^*−*^/^*−*^ cells without HU treatment (Fig. 3A, B). Unlike CDC45, only a minor fraction of LAP2α is associated with unperturbed forks undergoing replication, but it becomes more abundant when forks stall (Fig. 3A and Additional file 2: Figure S1A). Possibly, there is a transient interaction between LAP2α and the replicating chromatin, while LAP2α-directed RPA loading in HU-challenged cells is controlled by certain factor that functions to actively recruit the LAP2α-RPA complex or retain LAP2α on damaged chromatin.

To further investigate the role of LAP2α in promoting RPA deposition in stressed condition, ssDNA-pull-down assay was performed with nuclear extracts from wild type (*Lap2α* ^*+*^/^*+*^) and *Lap2α* ^*−*/*−*^ MEFs to assess ssDNA-RPA formation. In this assay, it has been proven that 70-nucleotide (nt) length of ssDNA may, to certain extent, mimic perturbed replication fork and will efficiently activate replication stress response [39, 40]. The results indicated that Lap2α deficiency dramatically impaired RPA loading (Additional file 2: Figure S1B). The same is true when ssDNA-pull-down assays were performed with LAP2α-knockdown cells (Additional file 2: Figure S1C). Furthermore, we demonstrated that LAP2α/wt, but not LAP2α/2RE, could efficiently restore RPA binding in LAP2α-depleted cells (Additional file 2: Figure S1D). At the time of analysis, *Lap2*α knockout or LAP2α knockdown did not significantly alter the cell-cycle profile (Additional file 2: Figure S1E and S1F). In agreement with the observations from iPOND assays under HU-challenged condition, we found LAP2α as well as its variant LAP2α/2RE could be also pulled down with RPA by biotinylated-ssDNA (Additional file 2: Figure S1D).

Then, we examined whether LAP2α affects RPA foci formation on stalled or collapsed replication forks. As shown in Fig. 3C and D, discrete foci of RPA1 and RPA2 are observed upon HU treatment in the nuclei of EdU-positive cells. Remarkably, LAP2α depletion impaired the recruitment of RPA1 and RPA2 to the blocked forks, and the defective RPA foci formation could be significantly overcome by overexpression of LAP2α/wt, but not LAP2α/2RE (Fig. 3C, D). Similar results were obtained when these cells were challenged with camptothecin (CPT) (Fig. 3C, D), which poisons Topoisomerase I and leads to stalling or collapse of replication forks [41, 42]. Next, we tested whether LAP2α is required for RPA loading onto blocked replication fork using the Lac operon-Lac repressor (LacO-LacI) tethering system [43]. In this approach, the stably integrated LacO operator is inherently and tightly bound by LacI, and the LacO-LacI nucleating complex poses an obstacle to DNA polymerase progression, leading to site-specific replication fork blockage [43, 44]. A robust accumulation of RPA around the LacO arrays was observed in cells expressing an mCherry-LacI fusion protein, manifested by the co-localization of RPA1 with mCherry-LacI (Additional file 2: Figure S1G). LAP2α knockdown prominently compromised RPA accumulation on mCherry-LacI marked blocked replication site, and this effect could be reverted by forced expression of LAP2α/wt, but not LAP2α/2RE (Additional file 2: Figure S1G). These results further support the argument that LAP2α is essentially required for RPA deposition in responding to replication stress.

Next, we wondered whether LAP2α could control RPA loading to DNA lesions. To this end, breaks were induced by laser micro-point equipped with 365-nm UV laser beam that is generally used to create single-strand breaks (SSBs) and DSBs, and the kinetics of RPA deposition was first evaluated in *Lap2*α^*+*^/^*+*^ and *Lap2*α^*−*^/^*−*^ MEFs. Live cell imaging followed by confocal microscopy analysis indicated that Lap2α deficiency resulted in a low efficiency of RPA loading, evidenced by a mild reduction of laser stripe formation of GFP-tagged RPA1 at early response phase and a remarkably weakened pattern at late time points (Fig. 3E and Additional file 2: Figure S1H). The increasing difference of RPA intensity at laser stripes from early to late time points in wild type versus knockout cells (Additional file 2: Figure S1H), suggests that there could be a differential extent of requirement of LAP2α for RPA loading during a progressive DNA end resection-coupled RPA deposition process. Furthermore, we showed that LAP2α/wt could significantly restore LAP2α depletion-associated defects of RPA1 recruitment, whereas LAP2α/2RE failed to do so (Fig. 3F). Taken together, our results support the notion that LAP2α acts as an essential regulator in promoting RPA association with damaged chromatin in the presence of replication stress or DNA breaks, indicating that LAP2α may chaperone RPA in vivo.

Since all splicing variants of LAP2 share the same N-terminal region, we wondered whether other isoforms of LAP2 anchored at nuclear envelope act similarly as LAP2α. We first found LAP2β knockdown had marginal effect on site-specific RPA recruitment upon replication fork stalling (Additional file 2: Figure S1I). Moreover, rescue experiments indicated that the defects of RPA accumulation induced by LAP2 depletion could be efficiently restored by overexpression of siRNA-resistant LAP2α, but not that of LAP2β (Additional file 2: Figure S1J). Although we could not exclude the possibility that the inner membrane-associated LAP2s may function to facilitate RPA binding to ssDNA that is adjacent to nuclear envelope, these results suggest that LAP2α is the major splicing variant of LAP2 that controls RPA loading in the nucleoplasm territories. Meanwhile, we examined whether Lamin A/C, which has been involved in DNA damage repair [45–47], plays a role in RPA loading. The results indicated that the recruitment of GFP-RPA1 was largely unaffected in *Lmna*^*−*/*−*^ MEFs (Additional file 2: Figure S1K). Similar results were obtained when the foci formation of RPA1 and RPA2 were examined in *Lmna*^*−*/*−*^ MEFs and Lamin A/C knockout U2OS cells (Additional file 2: Figure S1L and S1M). Consistently, co-immunoprecipitation experiments showed that Lamin A/C could not be precipitated by RPA1 (Additional file 2: Figure S1N). Thus, we propose that Lamin A/C is not involved in RPA loading and LAP2α functions independently of Lamin A/C in this process.

### LAP2α maintains DNA replication integrity through loading RPA onto ssDNA

**Fig. 4:**
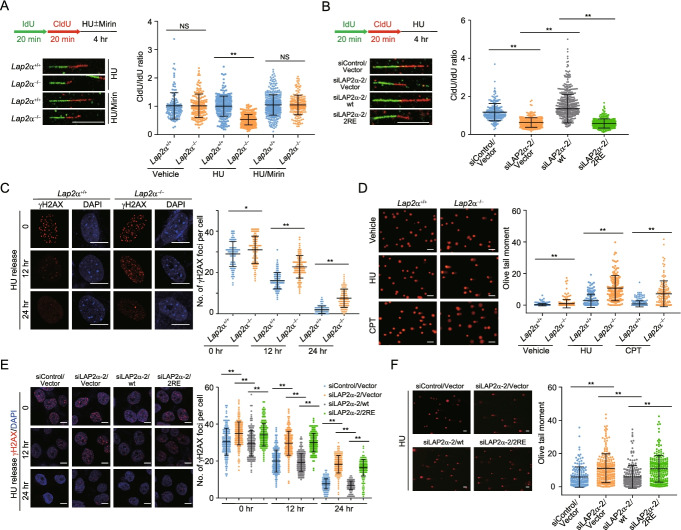
LAP2α maintains DNA replication integrity through loading RPA onto ssDNA. **A** DNA fiber assay with *Lap2α*^*+*/*+*^ or *Lap2α*^*−*/*−*^ MEFs. Cells were sequentially labelled with DNA analog IdU and CldU for the indicated time followed by HU (4 mM, 4 h) treatment in the absence or presence of mirin (100 μM, 4 h). The ratios of CldU and IdU length were calculated in each treatment (*n* > 150). Scale bar, 10 μm. **B** pLenti-vector, LAP2α/wt or LAP2α/2RE stably integrated U2OS cells were transfected with control siRNA or LAP2α 3′UTR siRNA and experiments analogous to **A** were performed (*n* > 150). Scale bar, 10 μm. **C** *Lap2α*^*+*/*+*^ or *Lap2α*^*−*/*−*^ MEFs were treated with 2 mM HU for 16 h and released followed by γH2AX staining and confocal microscopy inspection. The foci number of γH2AX per cell in each treatment was quantified (*n* > 100). Scale bar, 10 μm. **D** Accumulation of damaged DNA was examined and quantified with alkaline comet assay in HU (2 mM, 4 h) or CPT (1 μM, 2 h) treated *Lap2α*^*+*/*+*^ or *Lap2α*^*−*/*−*^ MEFs (*n* > 150). Scale bar, 100 μm. **E** pLenti-vector, LAP2α/wt or LAP2α/2RE stably integrated U2OS cells were transfected with control siRNA or LAP2α 3′UTR siRNA and experiments analogous to **C** were performed (*n* > 150). Scale bar, 10 μm. **F** pLenti-vector, LAP2α/wt or LAP2α/2RE stably integrated U2OS cells were transfected with control siRNA or LAP2α 3′UTR siRNA followed by HU treatment and experiments analogous to **D** were performed (*n* > 300). Scale bar, 100 μm. Data are mean ± SDs for **A** and **B** from biological duplicate experiments, and **C–F** from biological triplicate experiments. *P < 0.05; **P < 0.01; NS, not significant; Mann-Whitney test

Because RPA functions as an essential module in binding ssDNA and protecting stalled forks from nucleolytic degradation, we wondered whether LAP2α plays a role in DNA replication integrity. To this end, DNA fiber analysis was carried out to assess replication fidelity at single-molecule resolution. First, *Lap2α*^*+*/*+*^ or *Lap2α*^*−*/*−*^ MEFs were labelled sequentially with nucleoside analogs 5′-iodo-2-deoxyuridine (IdU) and 5′-chloro-2-deoxyuridine (CldU), and the track lengths of single DNA fibers were measured following HU treatment. The ratios of the lengths of adjacent CldU and IdU approximated unity under HU treatment in *Lap2α*^*+*/*+*^ MEFs (Fig. 4A), indicating that the integrity of stalled forks is largely unaffected during prolonged replication stress. In contrast, the CldU/IdU ratios were significantly reduced in HU-treated *Lap2α*^*−*/*−*^ MEFs (Fig. 4A), implying a defect in stalled fork protection. This was prevented by addition of the nuclease inhibitor mirin (Fig. 4A). Identical results were obtained upon analysis of fibers from LAP2α-knockdown human cells (Fig. 4B). Importantly, we showed LAP2α depletion-associated fork degradation could be successfully compensated by forced expression of LAP2α/wt, but not by LAP2α/2RE that is defective in binding and loading RPA (Fig. 4B). These results are consistent with the role of RPA in fork protection [17, 48] and raised the possibility that LAP2α functions to protect nascent strands on stalled replication forks via facilitating RPA deposition.

To further examine whether LAP2α is needed to maintain replication integrity, we analyzed the ability of LAP2α-deficient cells to recover from HU-induced replication arrest. After the release from HU treatment, γH2AX foci largely disappeared from control cells but persisted in *Lap2*α^*−*/*−*^ MEFs (Fig. 4C). Next, comet assays demonstrated that *Lap2*α-knockout cells contained markedly elevated levels of DNA breaks under either HU or CPT treatment (Fig. 4D). A moderate upregulation of damaged DNA was also observed in *Lap2α*-knockout cells without exogenous stress (Fig. 4C, D). Furthermore, we showed that LAP2α-knockdown associated accumulation of DNA damage in stressed condition could be efficiently reduced by forced expression of LAP2α/wt, but not that of LAP2α/2RE (Fig. 4E, F). Thus, we propose that LAP2α-promoted RPA deposition is critically required for cells to cope with replication stress thus maintaining replication fidelity.

### LAP2α-promoted RPA loading is required for ATR activation and homologous recombination

**Fig. 5:**
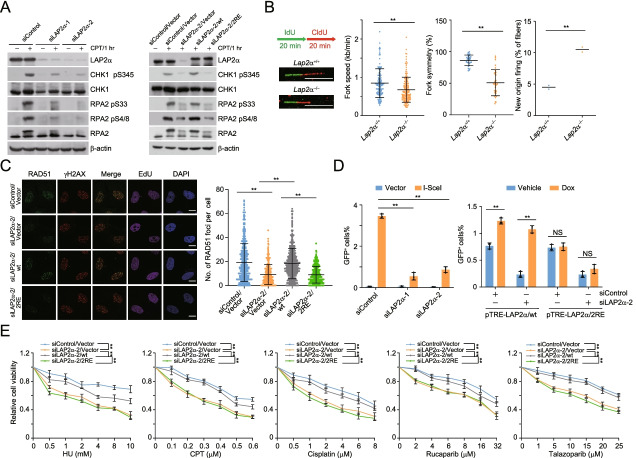
LAP2α-promoted RPA loading is required for ATR activation and homologous recombination. **A** Immunoblotting analysis of ATR kinase activity. Control U2OS cells and LAP2α/wt or LAP2α/2RE stably integrated U2OS cells were transfected with indicated siRNAs and in the absence or presence of CPT (1 μM, 1 h). The cellular extracts were collected to examine CPT-induced phosphorylation events. **B** DNA fiber assay with *Lap2α*^*+*/*+*^ or *Lap2α*^*−*/*−*^ MEFs. Cells were sequentially labelled with DNA analog IdU and CldU for the indicated time. Fork speed (*n* > 120) and fork symmetry (*n* > 25) were determined by measuring the length of CldU track, and the percentage of new origins was quantified with only CldU staining fibers. **C** Immunostaining and confocal microscopy analysis of RAD51 foci formation. LAP2α/wt or LAP2α/2RE stably integrated U2OS cells were transfected with LAP2α 3′UTR siRNA, exposed to IR (4 Gy) and cultured for 3 h followed by 1 h EdU labelling before collection. The foci number of RAD51 per EdU-positive cell in each group was quantified (*n* > 150). **D** Homologous recombination efficiencies monitored by DR-GFP reporter assays. Control DR-GFP U2OS cells and DR-GFP U2OS cells that allow for Dox-inducible expression of stably integrated pTRE-LAP2α/wt or pTRE-LAP2α/2RE were co-transfected with HA-I-SceI and the indicated siRNAs. Cells were treated with vehicle or Dox (1 ng/ μl) for 48 h to induce the expression of LAP2α/wt or LAP2α/2RE. The proportions of GFP-positive cells were determined by flow cytometry. **E** Survival analysis of U2OS cells expressing LAP2α 3′UTR siRNA and LAP2α/wt or LAP2α/2RE under different drug treatment. Data are mean ± SDs for **B** from biological duplicate experiments, and **C–E** from biological triplicate experiments. **P < 0.01; NS, not significant; Mann-Whitney test for **B** and **C**; one-way ANOVA for **D**; two-way ANOVA for **E**. Scale bar, 10 μm

Next, we tested whether LAP2α plays a role in RPA-directed ATR activation upon replication stress. Immunoblotting analysis indicated that LAP2α depletion markedly reduced the phosphorylation level of RPA2 S33 and CHK1 S345, which are substrates of ATR and generally used to monitor ATR activity (Fig. 5A). Unlike LAP2α/wt, LAP2α/2RE overexpression was incapable of reverting LAP2α-depletion-associated defects of ATR activation in LAP2α-knockdown cells (Fig. 5A). Similar results were obtained when the intensity of RPA2 S33 phosphorylation was examined with fluorescent immunostainings in HU-challenged EdU-positive cells (Additional file 2: Figure S2A). Meanwhile, we found *Lap2α*^*−*/*−*^ MEFs in normal conditions showed mildly reduced fork elongation rates, accompanied by a decrease in the symmetry of bidirectional replication forks and an increase in new origin firing (Fig. 5B). This is consistent with the understanding that ATR inhibition leads to compromised replication fork elongation and unscheduled replication timing [49].

To assess the role of LAP2α-promoted RPA loading in DSB repair, we then examined the foci formation of RAD51 that functions downstream of RPA [22]. As shown in Fig. 5C, LAP2α depletion resulted in a significant impairment of RAD51 foci formation upon irradiation, and this effect could be faithfully reverted by LAP2α/wt, but not LAP2α/2RE. Meanwhile, we found that, in LAP2α-deficient cells, the expression of RAD51 and RPA was not affected (Additional file 2: Figure S2B). Importantly, we showed LAP2α depletion had minor effect on BrdU foci or laser stripe formation under non-denaturing conditions (Additional file 2: Figure S2C and Figure S2D), excluding the possibility that LAP2α-promoted RPA deposition on DNA damage sites is a secondary effect of enhanced DNA end resection. Next, the DNA repair efficiency was monitored in DR-GFP U2OS cells, a well-known tool for detecting HR activity manifested by alterations of percentage of GFP-positive cells [50, 51]. The results showed LAP2α knockdown significantly compromised the efficiency of HR (Fig. 5D). In support of the importance of the functional link between LAP2α and RPA, we found that overexpression of LAP2α/wt, but not LAP2α/2RE, could evidently compensate the HR defects in LAP2α-knockdown cells (Fig. 5D and Additional file 2: Figure S2E). Collectively, these data indicate that LAP2α-promoted RPA loading plays an important role in HR repair of DSBs.

We next examined the effect of LAP2α depletion on cell survival upon replication stress induced DNA damage. The results indicated that LAP2α-depleted cells were more vulnerable to HU, CPT, or cisplatin (a DNA crosslinker that will generate replication stress) (Fig. 5E). Since long-term treatment with these agents will lead to fork collapse and the repair of resultant DSBs relies on HR, we envisioned that the hypersensitivity of LAP2α-deficient cells reflects an additive effect of fork instability, ATR inactivation, and HR deficiency. As cells with defects in HR or ATR signalling are synthetically lethal with poly(ADP-ribose)-polymerase (PARP) inhibition [52–54], we wondered whether cells lacking LAP2α are hypersensitive to PARP inhibitors (PARPis). Indeed, LAP2α-depleted cells exhibited a much lower survival rate following rucaparib or talazoparib treatment (Fig. 5E). Furthermore, cell survival analysis confirmed that LAP2α-protecting cells from replication stress-associated DNA damage depends on its association with and loading of RPA, as LAP2α/2RE failed to compensate LAP2α depletion-induced effects (Fig. 5E). Collectively, these data suggest that LAP2α is critically involved in responding to replication stress and repair of DNA lesions via actively promoting RPA deposition.

### LAP2α is engaged into damaged chromatin in a PARP1-dependent manner

As LAP2α could be pulled down by ssDNA-nucleated protein complexes, and it is present on nascent ssDNA upon HU challenging, we suspected that LAP2α is able to localize to damaged chromatin. Indeed, confocal microscopy analysis confirmed that LAP2α was recruited to mCherry-LacI and RPA marked blocked forks (Additional file 2: Figure S3A). Meanwhile, we showed that GFP-tagged LAP2α rather than LAP2β could form evident micro-IR path (Additional file 2: Figure S3B). In agreement with the observations from ssDNA-pull-down assays (Additional file 2: Figure S3C), endogenous LAP2α was co-localized with γH2AX and RPA1 and it departed from DNA lesions progressively (Additional file 2: Figure S3D and S3E). Then, we sought to explore whether RPA recruits LAP2α to damaged chromatin. Interestingly, RPA1 depletion has minor effect on the engagement of LAP2α around damaged DNA sites (Additional file 2: Figure S3F), and LAP2α/2RE behaved similarly as LAP2α/wt when its recruitment kinetics was examined by micro-IR (Additional file 2: Figure S3G). This is consistent with the observations from ssDNA-pull-down assays, from which we can see LAP2α/2RE could be also precipitated with ssDNA-RPA complex (Additional file 2: Figure S1D). These results imply that the physical contact between RPA and LAP2α is likely lost once RPA has been deposited, and LAP2α is finally removed from damaged chromatin after loading RPA.

**Fig. 6:**
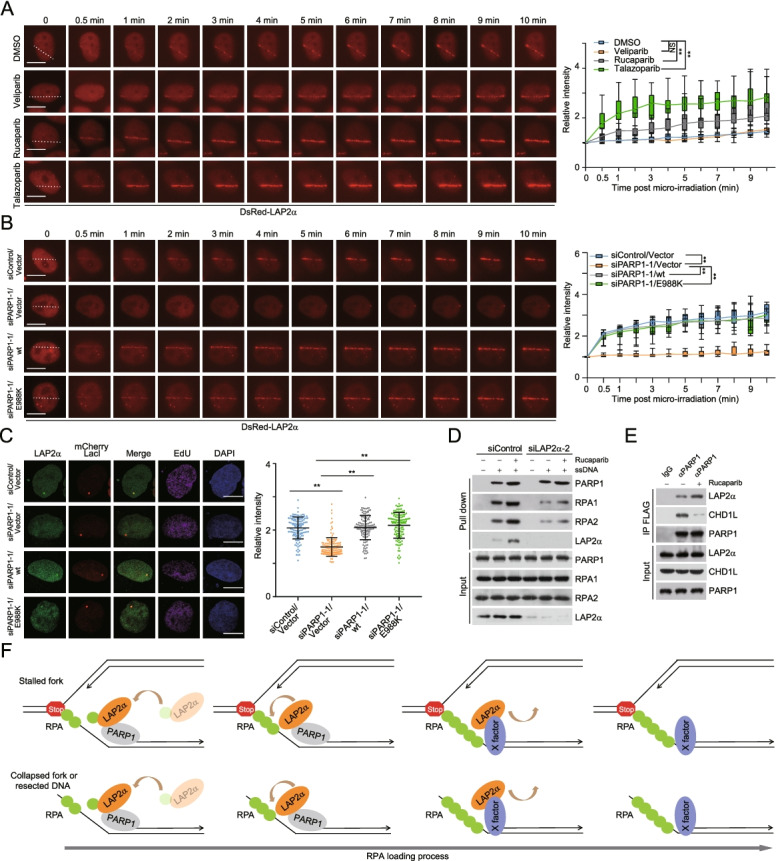
LAP2α is engaged into damaged chromatin in a PARP1-dependent manner. **A** Laser micro-IR (35% laser energy) followed by live cell imaging analysis of DsRed-LAP2α recruitment kinetics in PARP1 inhibitor (PARPi)-treated U2OS cells. DsRed-LAP2α expressing cells were pre-treated with different PARPis (10 μM) for 4 h before laser micro-IR. Fluorescence intensities in micro-irradiated areas relative to the nuclear background were quantified (*n* > 20). **B** Laser micro-IR (50% laser energy) followed by live cell imaging analysis of DsRed-LAP2α recruitment kinetics in PARP1-knockdwon or -overexpression U2OS cells. pLenti-vector, PARP1/wt, or PARP1/E988K stably integrated U2OS cells were co-transfected with control siRNA or PARP1 3′UTR siRNA (siPARP1-1) and DsRed-LAP2α. Fluorescence intensities in micro-irradiated areas relative to the nuclear background were quantified (*n* > 20). **C** U2OS-LacO cells expressing mCherry-LacI, PARP1 variants, and PARP1 3′UTR siRNA were labelled with EdU for 1 h before immunostaining and confocal microscopy analysis. The intensity of LAP2α foci in mCherry-LacI and EdU-positive cells was quantified and normalized to the nuclear background (*n* > 100). **D** ssDNA-pull-down analysis of LAP2α accumulation, PARP1 binding, and RPA loading with nuclear extracts from LAP2α-knockdown U2OS cells under rucaparib (10 μM, 4 h) treatment. 5’ biotin-labelled 70-nt ssDNA was used in pull-down assays. **E** Co-immunoprecipitation analysis of the interaction between LAP2α and PARP1 with cellular extracts from U2OS cells pre-treated with vehicle or rucaparib (10 μM, 4 h). **F** A proposed regulatory model for PARP1 in directing LAP2α-promoted RPA loading at damaged chromatin. X-factor represents the molecular machinery that may function to retain the longer occupancy of LAP2α on damaged chromatin. Data are mean ± SDs for **A–C** from biological triplicate experiments. **P < 0.01; NS, not significant; two-way ANOVA for **A** and **B**; Mann-Whitney test for **C**. Scale bar, 10 μm

To understand the underlying mechanisms that determine LAP2α engagement, we generated HeLa cells stably expressing FLAG-LAP2α. Affinity purification and mass spectrometry analysis identified poly(ADP-ribose) polymerase 1 (PARP1) as a potential LAP2α-interacting protein, and the interaction was further confirmed by co-immunoprecipitation assays (Additional file 2: Figure S3H, S3I, and S3J; and Additional file 3: Table S2). Because one of the earliest events in DDR is the recruitment of PARP1 to diverse types of DNA damage, we hypothesized that PARP1 may act to localize LAP2α. Indeed, knockdown of PARP1 almost completely impaired laser stripe formation of LAP2α (Additional file 2: Figure S3K). Cells were then treated with different PARPis including veliparib, rucaparib, and talazoparib to examine the contribution of the polymerase activity of PARP1. Surprisingly, the results indicated that, although the recruitment of CHD1L, a PAR binding protein [55], was severely abolished (Additional file 2: Figure S3L), the accrual of LAP2α on damaged chromatin was not weakened, but strengthened, and the alterations were proportional to the chromatin trapping efficiency of these drugs [54], among which talazoparib had the strongest effect (Fig. 6A). These results indicate that LAP2α is co-trapped with PARP1 at DNA lesions upon PARP inhibition and PARP1-promoted LAP2α recruitment does not rely on the catalytic activity of PARP1.

Next, we showed that, in PARP1-depleted cells, the defective recruitment of LAP2α could be significantly overcome by either wild type PARP1 (PARP1/wt) or PARylation activity-deficient PARP1 (PARP1/E988K) [56], in which glutamate 988 is replaced by lysine, further indicating that the enzymatic activity of PARP1 is dispensable for LAP2α localization to damaged chromatin (Fig. 6B, C, and Additional file 2: Figure S3M). Meanwhile, ssDNA-pull-down experiments revealed that rucaparib treatment enhanced LAP2α accumulation, PARP1 binding, and RPA loading, while LAP2α depletion markedly compromised PARPi-induced RPA deposition (Fig. 6D). Furthermore, co-immunoprecipitation analysis demonstrated that PARPi treatment strengthened but not weakened the association of LAP2α with PARP1 (Fig. 6E), and LAP2α could equally bind to PARP1/wt and PARP1/E988K (Additional file 2: Figure S3N). Consistently, PARP1 knockdown weakened the loading of RPA on laser-generated DNA lesions or perturbed replication fork (Additional file 2: Figure S3O and S3P). Taken together, these results indicate that PARP1 promotes LAP2α recruitment at damaged chromatin in a PARylation activity-independent manner and provides mechanistic insight of why LAP2α is preferentially and selectively involved in damaged chromatin-associated RPA loading (Fig. 6F).

## Discussion

A central regulator of eukaryotic DNA metabolism is ssDNA-RPA intermediate. However, it remains to be investigated that how the formation of ssDNA-RPA platform is controlled during replication stress or at DNA lesions. In this study, we characterized LAP2α as an essential regulator for RPA loading to ssDNA and this process requires physical contacts between LAP2α and RPA1. Importantly, we characterized a separation-of-function mutant of LAP2α and revealed that LAP2α-promoted RPA loading onto ssDNA and its role in genome stability are largely uncoupled from its pRB- or/and Lamin A/C-associated functions, but specifically attributed to its physical and functional association with RPA. Interestingly, the selectivity of LAP2α-promoted RPA deposition on damaged chromatin is controlled by PARP1.

Since RPA is a critical regulator of DNA replication, it is important to understand whether LAP2α could favor RPA binding to ssDNA in this process. Indeed, when LAP2α was knocked out, a mild loss of RPA at active forks accompanying with a detectable accumulation of DNA breaks and a global reduction in DNA synthesis, albeit not dramatically, was observed under unstressed conditions in MEF cells. These results suggest that in normal cells, LAP2α may, to a certain extent, contribute to the delivery of RPA and maintenance of genome integrity. This argument is consistent with LAP2α expression feature: it is upregulated in proliferating cells and is downregulated upon cell-cycle exit or differentiation [34]. The moderate effect could be due to a weak activity of LAP2α itself in replication-coupled RPA deposition, or due to compensation from other factors that play a role of importance in assistance of RPA loading. For example, CDC45, an essential factor for the initiation and elongation process of replication, is proposed to guarantee a seamless deposition of RPA on newly emerging ssDNA at the nascent replication forks [32]. Indeed, comparing to LAP2α loss, CDC45 depletion exhibited more severe effects on RPA loading in replicating cells.

On the contrary, replication stress may potentially result in an accrual of ssDNA formation with larger stretches, where an instant requirement for more RPA binding must be fulfilled to maintain fork integrity [12]. Here, we revealed that LAP2α is particularly important for RPA deposition in this circumstance, as the association of RPA with chromatin was significantly reduced at stalled or collapsed forks globally or at site-specific replication blockage upon LAP2α loss. In agreement with these findings, we confirmed that LAP2α-promoted RPA loading is particularly important to uphold DNA replication integrity and genome stability. Considering that CDC45 is likely the major factor for RPA deposition on active replication forks [32] and LAP2α-promoted RPA loading is particularly important under replication stress, we propose that distinct molecular machineries could be utilized to control RPA binding to ssDNA under different DNA metabolic processes. Since the DBD-A domain of RPA interacts with both CDC45 and LAP2α, it is possible that CDC45-RPA and LAP2α-RPA complexes directed by DBD-A are assembled in a context-dependent manner. As cellular LAP2α is a multimeric protein channelled by its C-terminal region [36], it is worth characterizing whether this could affect its RPA loading activity in the future.

Excessive ssDNA, in the form of ssDNA gaps or resected ends of DSBs, can be generated by appropriate DNA nucleases at broken sites [57, 58]. Consistently, we found that LAP2α is also critically required for RPA loading to DNA lesions upon irradiation. In support of this, we showed that the activity of LAP2α in loading RPA to ssDNA is critically essential for cells to repair damaged DNA via HR, implying that LAP2α is generally involved in multiple aspects of RPA-dictated DNA metabolic signalling pathways. Interestingly, we showed LAP2α is prominently localized to damaged chromatin in the occurrence of replication stress or DNA breaks, and the recruitment of LAP2α is dependent on its physical association with PARP1. This, to certain extent, explains that why LAP2α is preferentially responsible for RPA loading onto damaged chromatin. We noticed that LAP2α is engaged into damaged chromatin in a dynamic manner, but what determines its clearance remains to be investigated. Considering the rapid turnover of PARP1 and PAR modification on damaged chromatin, we speculated that the relatively longer retention of LAP2α must be controlled by other undefined factors. LAP2α has been reported to be PARylated upon DNA damage [59, 60], while we demonstrated the enzymatic activity of PARP1 is largely irrelevant to LAP2α engagement, implying that LAP2α, in particular of its PARylated form, may play additional roles in DDR. In contrast to Bártová’s study [61], we showed both tagged and endogenous LAP2α could be efficiently recruited to local DNA lesions, where it co-localized with γH2AX and RPA1. The discrepancy may arise either from different UV generator systems we took or from distinct cell types we utilized.

In view of the recently reported role of LAP2α in transcriptional regulation and chromatin conformation alterations [62, 63], it is important to decipher whether LAP2α’s activity in genome surveillance could be specifically attributed to RPA loading. Many of the established functions of LAP2α map to its unique C-terminal region. For example, residues 415-615 of LAP2α interact with pRB [64], and the extreme C-terminus from residue 616 to 693 binds to Lamin A/C [65]. LAP2α determines the distribution of Lamin A/C in the nucleoplasm in general [37] and on distinct chromatin regions in specific [62], and the LAP2α-Lamin A/C-pRB complex has been shown to influence cell-cycle progression and to control the balance between cell proliferation and differentiation [37, 64]. Meanwhile, LEM-like domain of LAP2α confers its interaction with transcription factor GLI1 [35], and LEM domain confers BANF1- or BAF-binding activity [36, 66]. In contrast to these binding themes, we revealed that the flexible liker between LEM-like domain and the LEM domain is responsible for RPA binding. Importantly, deletion of this linker region or mutation of the key residues for RPA association had marginal effect on the interaction of pRB or Lamin A/C with LAP2α, and LAP2α/2RE failed to efficiently bind and load RPA and could not compensate LAP2α depletion-induced RPA loading defects and genome instability. Meanwhile, we found Lamin A/C could not be precipitated by RPA1 and it is not involved in RPA loading at damaged chromatin. Thereby, we propose that LAP2α/2RE acts as a separation-of-function mutant that could be used to successfully differentiate LAP2α-governed biological process, and LAP2α-promoted RPA loading and genome integrity is, at least, uncoupled from pRB- or/and Lamin A/C-controlled signalling pathway. However, additional changes induced by LAP2α deficiency cannot be overlooked, exemplified by the findings that LAP2α is potentially involved in telomere maintenance [67] as well as 53BP1 accumulation [61]. The intrinsic ssDNA binding activity of RPA excludes the possibility that LAP2α functions to directly recruit RPA, whereas it remains an open question that whether LAP2α is involved in the controlling of RPA removal or retention, which may also affect RPA accumulation at damaged chromatin. It has been reported that LAP2α likely behaves as a tumor suppressor through repressing the transcriptional activity of E2F by forming complex with pRb and Lamin A/C [34]. Given the essentiality of LAP2α-promoted RPA loading in genome stability, it will be worth exploring that whether LAP2α-promoted RPA loading or its dysregulation plays a role in tumorigenesis.

## Conclusions

Our study provides mechanistic insight of RPA deposition in response to DNA damage including replication stress and DSBs. We propose that LAP2α functions as a chaperone factor for RPA loading to preserve genome stability in mammalian cells.

## Methods

For a detailed description of all methods, see the Additional file 4: Supplemental Methods.

### Immunofluorescence

Cells on glass coverslips (BD Biosciences) were fixed with 4% paraformaldehyde and permeabilized with 0.2% Triton X-100 in PBS. Samples were blocked in 5% donkey serum in the presence of 0.1% Triton X-100 and stained with the appropriate primary and secondary antibodies coupled to Alexa Fluor 488, 594, or 647 (Invitrogen). Confocal images were captured on a Zeiss LSM 900 microscope with a × 63 oil objective. To avoid bleed-through effects in double-staining experiments, each dye was scanned independently in a multi-tracking mode. When inspection of nuclear wide dispersed RPA1, RPA2, or RPA2 pS33 foci, cells were pre-treated with 0.5% Triton X-100 for 5 min on ice to extract non-chromatin fractions and fixed with 3% paraformaldehyde and 2% sucrose for 15 min at room temperature. Cells were then permeabilized with 0.5% Triton X-100 for 5 min on ice then incubated in blocking buffer (0.1% Triton X-100, 5% donkey serum in PBS) for 1 h at room temperature. For non-denaturing BrdU staining, cells were labelled with 10 μM BrdU for 24 h. For S phase discrimination, U2OS-LacO cells were pulsed with 10 μM EdU at 37 °C for 1 h before fixation. Incorporated EdU was click-labelled by using keyFluor 647-azide (Keygen Technologies) according to the manufacturer’s instructions.

### Isolation of proteins on nascent DNA (iPOND)

In brief, 2~3 × 10^8^ cells were labelled with 10 μM EdU (Thermo Fisher Scientific) for 10 min to detect nascent forks. For stalled forks, cells were treated with HU for 1 h in the continued presence of EdU. The harvested cells were then fixed with 1% formaldehyde in PBS solution for 20 min at room temperature followed by quenching of the crosslinking reaction with 1.25 M glycine. Cells were then harvested and incubated in a permeabilization buffer (0.25% Triton X-100/PBS) at room temperature for 30 min. Then, cells were washed at 4 °C with 0.5% BSA/PBS, then PBS alone, followed by incubation in the “click” (10 mM sodium ascorbate, 2 mM CuSO_4_, 10 mM biotin-azide, in PBS) or “no-click” (i.e., no biotin-azide) reaction cocktail for 2 h at room temperature. After the reaction, cells were re-suspended in lysis buffer (1% SDS, 50 mM Tris-HCl, pH 8.0) containing protease inhibitor (Roche) and cell lysates were sonicated four times by using a Bioruptor (Diagenode) at 4 °C (30 s on and 30 s off) to generate 200–400 bp DNA fragments. After centrifugation, EdU-labelled DNA was immunoprecipitated from supernatants by incubation with streptavidin-MyOne T1 beads (Thermo Fisher Scientific; pre-washed three times with PBS) for 4 h at 4 °C. The streptavidin-agarose beads containing the captured DNA-protein complexes were then centrifuged for 3 min at 1800*g*. After washing for 5 min each with 1 mL cold lysis buffer, 1 mL of 1 M NaCl, and twice more with 1 mL lysis buffer, the beads were supplemented 1:1 (v/v of packed beads) with 2 × SDS-PAGE loading buffer and incubated at 95 °C for 25 min to liberate the proteins. SDS-PAGE fractionation and immunoblotting were then performed.

### ssDNA-pull down

A total of 30 pmol biotin-labelled 70-nt ssDNA (TGCAGCTGGCACGACAGGTTTTAATGAATCGGCCAACGCGCGGGGAGAGGCGGTTTGCGTATTGGGCGCT) and 30 μL streptavidin Sepharose beads were preincubated in PBS with rotation at 4 °C overnight. Then, one third of the biotin-labelled ssDNA was incubated with nuclear extracts in NETN buffer at 4 °C for 8 h or at room temperature for 1 h followed by washing and immunoblotting. Oligonucleotide titrations were performed with 1-, 5-, and 20-fold molar excesses of dT10 or dT30. The ssDNA was added to the immobilized LAP2α/RPA complexes and incubated for 1 h at room temperature under gentle rotation. After washing, bound RPA was analyzed by immunoblotting.

### DNA fiber

To check fork symmetry, new origin firing, and fork speed, cells were first labelled with IdU (25 μM) for 20 min, washed twice with media, and labelled with CldU (200 μM) for 20 min. To check fork degradation, cells were first labelled with IdU (25 μM) for 20 min, washed twice with media, and labelled with CldU (200 μM) for 20 min. After washing, cells were incubated with 4 mM HU ± 100 μM mirin for 4 h. Cells were then trypsinized and re-suspended in PBS to 7 × 10^5^ cells/ml. Then, 2 μl cells were mixed with 10 μl lysis buffer (200 mM Tris-HCl, pH 7.4, 50 mM EDTA, and 0.5% SDS) on a clean glass slide. After 2 min incubation, the slides were tilted at 15° to horizontal, allowing the lysate to slowly flow down along the slide. The slides were then air-dried, fixed in 3:1 methanol/acetic acid, and treated with 2.5 M HCl for 80 min. The slides were then blocked (5% BSA in PBS) for 30 min and incubated with anti-BrdU antibodies (BD Bioscience, 347580 against IdU, and AbD Serotec, MCA2060GA against CldU) in blocking buffer overnight. After washing, secondary antibodies coupled to Alexa Fluor 488 and 594 were diluted in PBS containing 5% BSA and incubated with cells at room temperature for 1 h. The slides were then washed 3 times with PBS. After washing, cells were mounted with an anti-fade solution and visualized under a Zeiss LSM 900 Fluorescence microscope. The length of all discrete fibers was measured by using ImageJ software. Fork symmetry was analyzed by measuring the length of CldU fiber (red) on each side. Fork speed was analyzed by measuring the length of CldU fiber based on an average fork elongation rate (1 μm roughly corresponds to 2 kb for each fiber). New origin firings were measured by counting only red fibers and compared to total fiber numbers.

### Statistics

Data from biological triplicate or duplicate experiments are presented as mean ± SDs. All statistical analyses involved were performed with SPSS 19. Two-tailed unpaired Student’s *t* test was used for comparing two groups of data. ANOVA with Bonferroni’s correction was used to compare multiple groups of data. For values not normally distributed, Mann-Whitney *U* test was used. *P* < 0.05 was considered statistically significant. Before statistical analysis, variation within each group of data and the assumptions of the tests were checked.

## Supplementary Information

**Additional file 1: Table S1.** SILAC-based quantitative mass spectrometry analysis of RPA1-containing protein complexes.

**Additional file 2: Figure S1.** (Fig. 3 continued). LAP2α promotes the loading of RPA onto damaged chromatin. **Figure S2.** (Fig. 5 continued). LAP2α-promoted RPA loading is required for ATR activation and homologous recombination. **Figure S3.** (Fig. 6 continued). LAP2 is engaged into damaged chromatin in a PARP1-dependent manner.

**Additional file 3: Table S2.** Mass spectrometry analysis of LAP2α-containing protein complexes.

**Additional file 4.** Supplemental Methods.

**Additional file 5.** Uncropped versions of all blots.

**Additional file 6.** Review history.

## Data Availability

All data generated or analyzed during this study are included in this published article and its supplementary information files. The mass spectrometry proteomics data have been deposited to the ProteomeXchange Consortium (http://proteomecentral.proteomexchange.org) via the iProX partner repository [68] with the dataset identifier PXD028105. All microscopy images are available at Figshare and can be accessed at (high content screening replicate 1: doi.org/10.6084/m9.figshare.19070321 [69], high content screening replicate 2: doi.org/10.6084/m9.figshare.19074440 [69], and other images: doi.org/10.6084/m9.figshare.c.5814131) [70].
