# Supplementary material for: LAP2α preserves genome integrity through assisting RPA deposition on damaged chromatin
Source: Genome Biol. 2022 Feb 28;23:64. doi: 10.1186/s13059-022-02638-6 (PMC8883701; doi:10.1186/s13059-022-02638-6)
Supplement: Supplementary file 2 — Additional file 2: Figure S1. (Fig. 3 continued). LAP2α promotes the loading of RPA onto damaged chromatin. Figure S2. (Fig. 5 continued). LAP2α-promoted RPA loading is required for ATR activation and homologous recombination. Figure S3. (Fig. 6 continued). LAP2 is engaged into damaged chromatin in a PARP1-dependent manner. [file 13059_2022_2638_MOESM2_ESM.pdf]

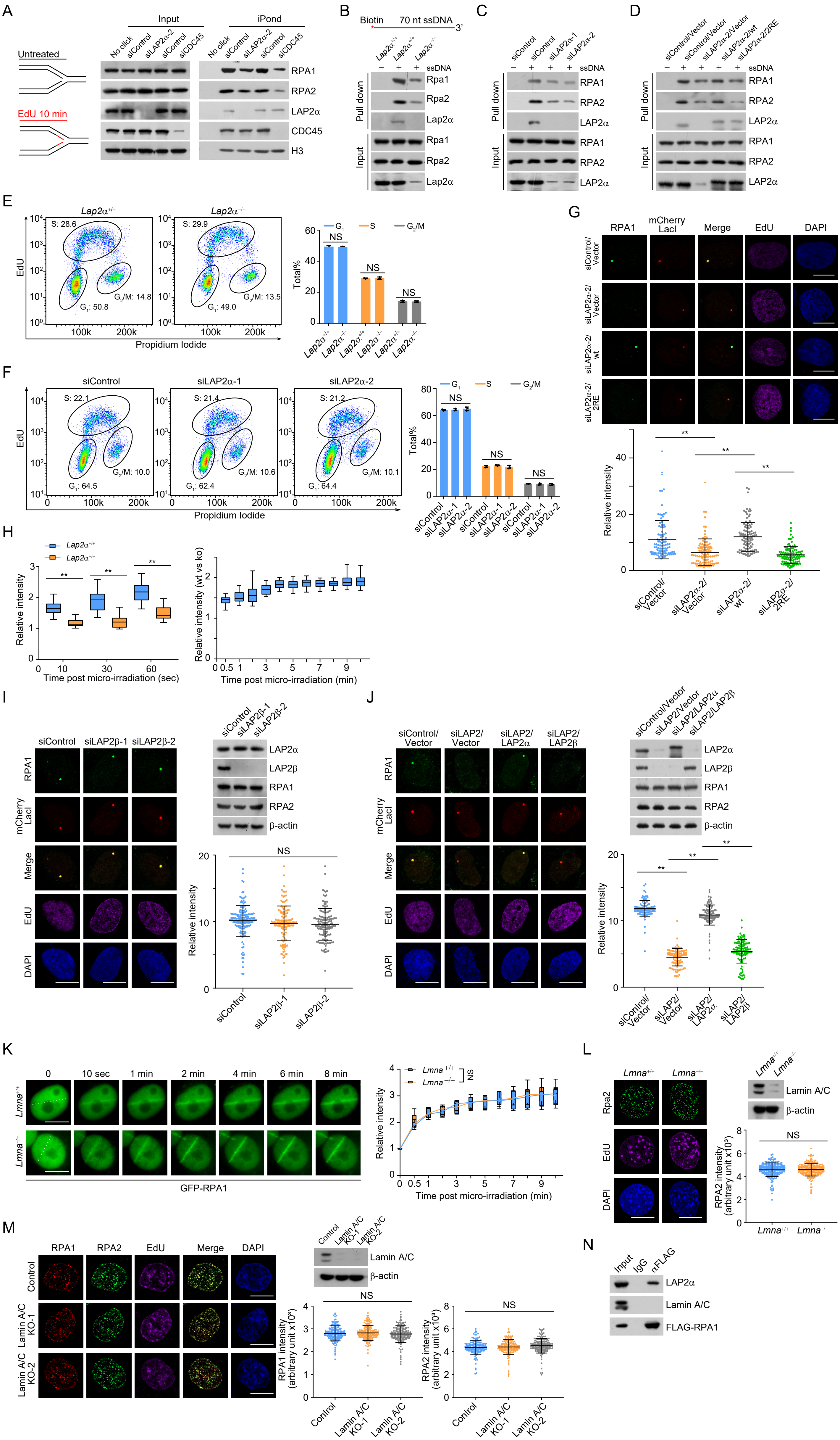

A
Untreated
EdU 10 min
Input
iPond
No click
siControl
siLAP2α-2
siCDC45
RPA1
RPA2
LAP2α
CDC45
H3
B
Biotin
70 nt ssDNA
3'
Lap2α+/+
Lap2α−/−
ssDNA
Pull down
Input
Rpa1
Rpa2
Lap2α
C
siControl
siLAP2α-1
siLAP2α-2
RPA1
RPA2
LAP2α
D
siControl/Vector
siLAP2α-2/Vector
siLAP2α-2/wt
siLAP2α-2/2RE
E
S: 28.6
G2/M: 14.8
G1: 50.8
S: 29.9
G2/M: 13.5
G1: 49.0
EdU
Propidium Iodide
Total%
NS
F
S: 22.1
G2/M: 10.0
G1: 64.5
S: 21.4
G2/M: 10.6
G1: 62.4
S: 21.2
G2/M: 10.1
G1: 64.4
G
RPA1
mCherry LacI
Merge
EdU
DAPI
Relative intensity
H
Time post micro-irradiation (sec)
Relative intensity (wt vs ko)
Time post micro-irradiation (min)
I
siControl
siLAP2β-1
siLAP2β-2
LAP2β
β-actin
J
siControl/Vector
siLAP2/Vector
siLAP2/LAP2α
siLAP2/LAP2β
K
0
10 sec
1 min
2 min
4 min
6 min
8 min
Lmna+/+
Lmna−/−
GFP-RPA1
L
Rpa2
Lamin A/C
RPA2 intensity (arbitrary unit x10³)
M
Control
Lamin A/C KO-1
Lamin A/C KO-2
RPA1 intensity (arbitrary unit x10³)
N
Input
IgG
αFLAG
FLAG-RPA1

Figure S1 (Figure 3 continued). LAP2α promotes the loading of RPA onto damaged chromatin. (A) Proteins associated with replication forks were isolated by iPOND and detected by immunoblotting. LAP2α-knockdown or CDC45-knockdown U2OS cells were EdU-labelled for 10 min and harvested immediately for iPOND analysis. (B) Nuclear extracts from *Lap2α*$^{+/+}$ or *Lap2α*$^{-/-}$ MEFs were incubated with 5' biotin-labelled 70-nt ssDNA for 1 hr followed by pull-down and immunoblotting analysis. (C) Nuclear extracts from control or LAP2α-knockdown HeLa cells were incubated with 5' biotin-labelled 70-nt ssDNA for 1 hr followed by pull-down and immunoblotting analysis. (D) Experiments analogous to (C) with nuclear extracts from U2OS cells. pLenti-vector, LAP2α/wt or LAP2α/2RE stably integrated U2OS cells were individually transfected with control siRNA or LAP2α 3'UTR siRNA (siLAP2α-2) before collection. The exogenous LAP2α is fused to a 3 X FLAG tag (3-4 kDa) so that it migrates slower. (E) *Lap2α*$^{+/+}$ or *Lap2α*$^{-/-}$ MEFs were treated with 10 μM EdU for 1 hr and processed for cell cycle analysis by flow cytometry with propidium iodide and EdU stainings. (F) Experiments analogous to (E) with control or LAP2α-knockdown U2OS cells. (G) U2OS cells stably integrated with LacO arrays (U2OS-LacO cells) were co-transfected with mCherry-LacI, LAP2α variants, and LAP2α 3'UTR siRNA (siLAP2α-2), and cells were labelled with EdU for 1 hr followed by immunostaining and confocal microscopy analysis. The intensity of RPA1 foci in mCherry-LacI and EdU positive cells was quantified and normalized to the nuclear background (n > 100). (H) Fluorescence intensities of GFP-RPA1 at early time points in micro-irradiated areas were normalized to the nuclear background in *Lap2α*$^{+/+}$ or *Lap2α*$^{-/-}$ MEFs (upper, related to Figure 3E). The difference between the intensity of GFP-RPA1 laser stripe and the nuclear background in *Lap2α*$^{+/+}$ (wt) MEFs was normalized to that in *Lap2α*$^{-/-}$ (ko) MEFs (lower, related to Figure 3E). (I) Quantitative analysis of RPA1 foci formation at site-specific blocked replication fork in LAP2β-knockdown U2OS-LacO cells. U2OS-LacO cells were co-transfected with mCherry-LacI and LAP2β siRNAs, and cells were labelled with EdU for 1 hr followed by immunostaining and confocal microscopy analysis. The intensity of RPA1 foci in mCherry-LacI and EdU positive cells was quantified and normalized to the nuclear background (n > 100). The knockdown effect was examined by immunoblotting. (J) Quantitative analysis of RPA1 foci formation at site-specific blocked replication fork in U2OS-LacO cells expressing the indicated siRNAs and genes. U2OS-LacO cells were co-transfected with mCherry-LacI, LAP2 variants, and LAP2 5'UTR siRNA that is able to knockdown all LAP2 isoforms, and cells were labelled with EdU for 1 hr followed by immunostaining and confocal microscopy analysis. The intensity of RPA1 foci in mCherry-LacI and EdU positive cells was quantified and normalized to the nuclear background (n > 100). The knockdown and overexpression effects were examined by immunoblotting. (K) Laser micro-irradiation (IR) (50% laser energy) followed by live-cell imaging analysis of GFP-RPA1 recruitment kinetics in GFP-RPA1 expressing *Lmna*$^{+/+}$ or *Lmna*$^{-/-}$ MEFs. Fluorescence intensities in micro-irradiated areas relative to the nuclear background were quantified (n > 20). (L) Analysis of the foci formation of Rpa2 in *Lmna*$^{+/+}$ and *Lmna*$^{-/-}$ MEFs. Cells were treated with 2 mM HU for 3 hr and labelled with EdU for 1 additional hr followed by pre-extraction, fixation, immunostaining, and confocal microscopy analysis. The foci intensity in each cell was quantified and shown (n > 150). The Lamin A/C knockout effect was examined by immunoblotting. (M) Analysis of the foci formation of RPA1 and RPA2 in Lamin A/C knockout U2OS cells that were generated by CRISPR/Cas9 technology. Cells were treated with 2 mM HU for 3 hr and labelled with EdU for 1 additional hr followed by pre-extraction, fixation, immunostaining, and confocal microscopy analysis. The foci intensity in each cell was quantified and shown (n > 150). The Lamin A/C knockout effect was examined by immunoblotting. (N) Co-immunoprecipitation analysis of the interaction between RPA1 and Lamin A/C or LAP2α. Whole-cell lysates from U2OS cells expressing FLAG-RPA1 were pre-treated with DNase followed by immunoprecipitation and immunoblotting with antibodies against the indicated proteins. Data are mean ± SDs for (E), (F), (G), (H), (I), (J), (K), (L), and (M) from biological triplicate experiments. **$P < 0.01$; NS, not significant; one-way ANOVA for (E) and (F); Mann-Whitney test for (G), (I), (J), (L), and (M); two-way ANOVA for (H) and (K). Scale bar, 10 μm.

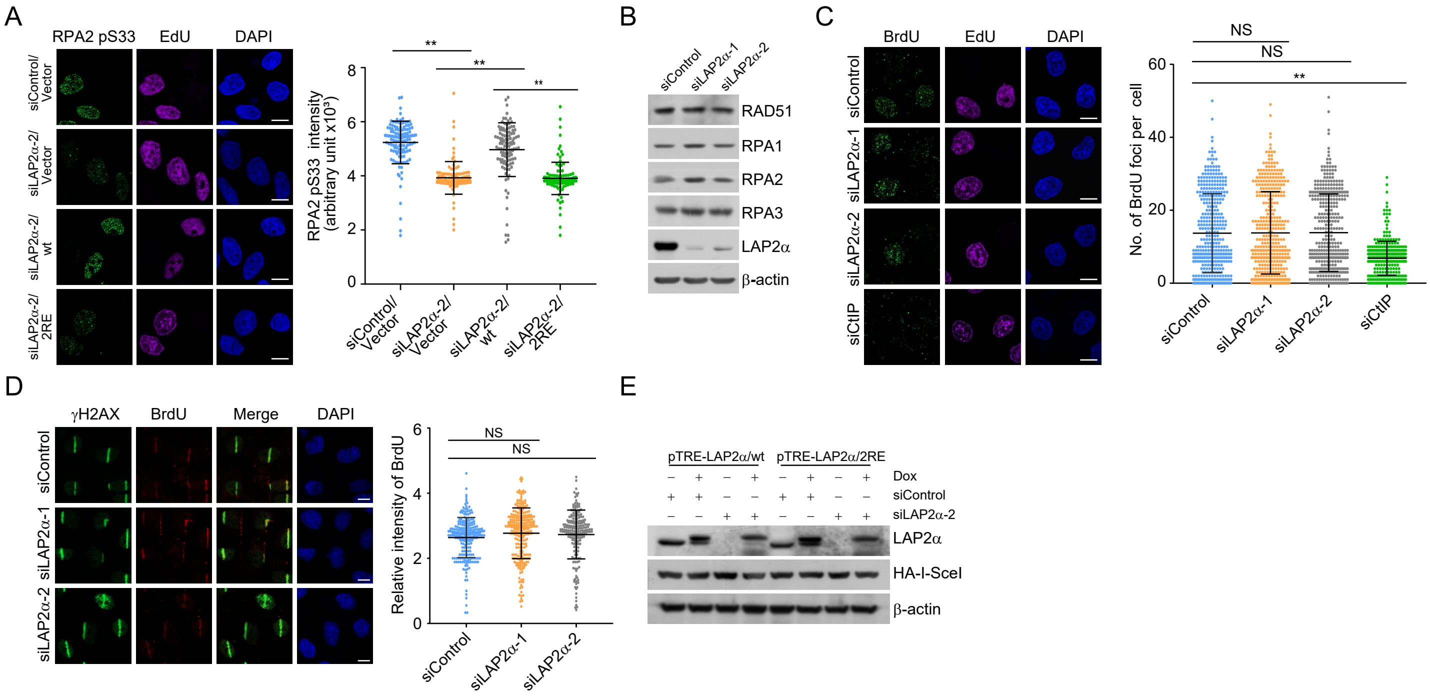

Figure S2 (Figure 5 continued). LAP2α-promoted RPA loading is required for ATR activation and homologous recombination. (A) RPA2 pS33 foci formation in U2OS cells expressing the indicated siRNAs and genes. pLenti-vector, LAP2α/wt or LAP2α/2RE stably integrated U2OS cells were transfected with control siRNA or LAP2α 3'UTR siRNA (siLAP2α-2) and treated with 2 mM HU for 3 hr and subsequently labelled with EdU for 1 hr followed by pre-extraction, fixation, immunostaining, and confocal microscopy analysis. The intensity of RPA2 pS33 foci in each cell was quantified and shown (n > 100). Data are mean ± SDs from biological triplicate experiments. ***P* < 0.01; Mann-Whitney test. Scale bar, 10 μm. (B) Immunoblotting analysis of the expression of the indicated proteins in control and LAP2α-knockdown U2OS cells. (C) Non-denaturing staining of BrdU in LAP2α-knockdown U2OS cells. Cells labelled with BrdU (10 μM, 24 hr) were exposed to IR (4 Gy) and cultured for 3 hr followed by 1 hr EdU labelling. The fixed cells were then immunostained and analyzed by confocal microscopy. The foci number of BrdU per cell in each treatment was quantified (n > 150). DNA end resection-promoting factor CtIP was taken as a positive control. Data are mean ± SDs from biological triplicate experiments. ***P* < 0.01; NS, not significant; Mann-Whitney test. Scale bar, 10 μm. (D) Non-denaturing staining of BrdU in LAP2α-knockdown U2OS cells. Cells were labelled with BrdU for 24 hr followed by laser micro-dissection (355 nm UV) and collected 0.5 hr later for immunostaining with antibodies against BrdU and γH2AX. The intensity of BrdU laser stripes inspected by confocal microscopy were quantified and normalized against the nuclear background (n > 150). Data are mean ± SDs from biological triplicate experiments. NS, not significant; Mann-Whitney test. Scale bar, 10 μm. (E) Immunoblotting analysis of the expression of the indicated proteins in DR-GFP U2OS cells (related to Figure 5D). DR-GFP U2OS cells that allow for Dox-inducible expression of stably integrated pTRE-LAP2α/wt or pTRE-LAP2α/2RE were transfected with HA-tagged I-SceI and indicated siRNAs. Before transfection, cells were treated with vehicle or Dox (1 ng/μl) for 48 hr to induce the expression of LAP2α/wt or LAP2α/2RE.

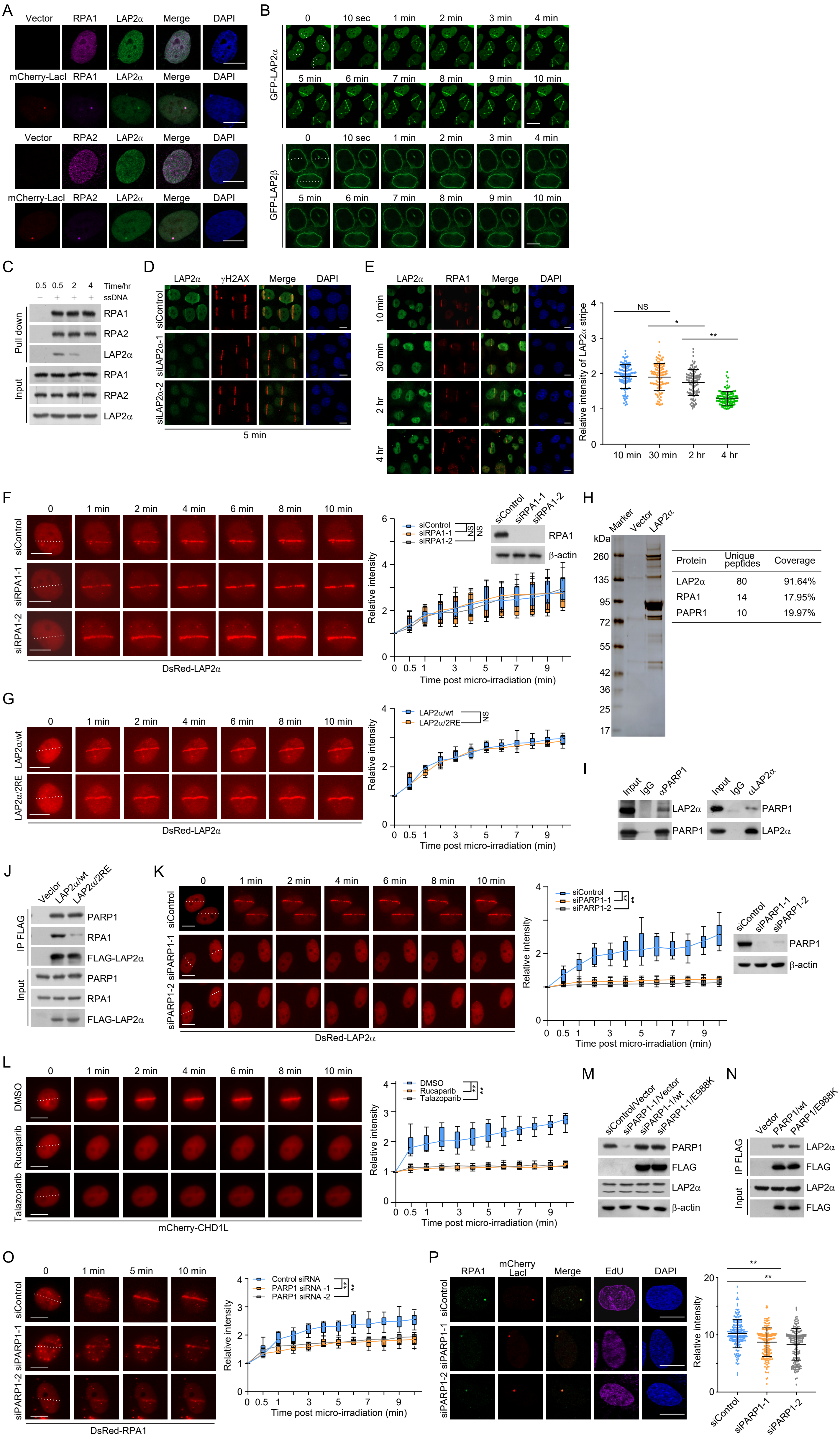
A
Vector
RPA1
LAP2α
Merge
DAPI
mCherry-LacI
RPA2
B
GFP-LAP2α
GFP-LAP2β
0
10 sec
1 min
2 min
3 min
4 min
5 min
6 min
7 min
8 min
9 min
10 min
C
Time/hr
ssDNA
Pull down
Input
D
γH2AX
siControl
siLAP2α-1
siLAP2α-2
5 min
E
10 min
30 min
2 hr
4 hr
Relative intensity of LAP2α stripe
NS
F
siRPA1-1
siRPA1-2
DsRed-LAP2α
Relative intensity
Time post micro-irradiation (min)
β-actin
G
LAP2α/wt
LAP2α/2RE
H
Marker
kDa
Protein
Unique peptides
Coverage
LAP2α 80 91.64%
RPA1 14 17.95%
PAPR1 10 19.97%
I
Input
IgG
αPARP1
αLAP2α
PARP1
J
IP FLAG
FLAG-LAP2α
K
siPARP1-1
siPARP1-2
L
DMSO
Rucaparib
Talazoparib
mCherry-CHD1L
M
siControl/Vector
siPARP1-1/Vector
siPARP1-1/wt
siPARP1-1/E988K
FLAG
N
PARP1/wt
PARP1/E988K
O
Control siRNA
PARP1 siRNA -1
PARP1 siRNA -2
DsRed-RPA1
P
mCherry LacI
EdU

Figure S3 (Figure 6 continued). LAP2α is engaged into damaged chromatin in a PARP1-dependent manner. (A) U2OS-LacO cells were transfected with control vector or mCherry-LacI for 24 hr followed by immunostaining with antibodies against LAP2α and RPA1 or RPA2. (B) Laser micro-IR (50% laser energy) followed by live-cell imaging analysis of GFP-LAP2α and GFP-LAP2β recruitment kinetics in GFP-LAP2α or GFP-LAP2β stably expressing U2OS cells. (C) Nuclear extracts from U2OS cells were incubated with 5' biotin-labelled 70-nt ssDNA for the indicated times followed by pull-down and immunoblotting analysis. (D) Control or LAP2α-knockdown U2OS cells were subjected to laser micro-dissection (355 nm UV) and collected at the indicated time points followed by immunostaining with antibodies against LAP2α and γH2AX and confocal microscopy analysis. (E) U2OS cells were subjected to laser micro-dissection (355 nm UV) and collected at the indicated time points followed by immunostaining with antibodies against LAP2α and RPA1. The intensity of LAP2α laser stripes inspected by confocal microscopy was quantified and normalized against the nuclear background ($n > 100$). (F) Laser micro-IR (50% laser energy) followed by live-cell imaging analysis of DsRed-LAP2α recruitment kinetics in RPA1-knockdwon U2OS cells. Fluorescence intensities in micro-irradiated areas relative to the background signal of the undamaged regions were quantified ($n > 20$). The knockdown effect was examined by immunoblotting. (G) Laser micro-IR (50% laser energy) followed by live-cell imaging analysis of the recruitment kinetics of DsRed-LAP2α/wt or DsRed-LAP2α/2RE in U2OS cells. Fluorescence intensities in micro-irradiated areas relative to the background signal of the undamaged regions were quantified ($n > 20$). (H) Immunopurification and mass spectrometry analysis of LAP2α-containing protein complex. Cellular extracts from HeLa cells stably expressing FLAG-LAP2α were immunopurified with anti-FLAG affinity beads and eluted with FLAG peptide. The eluates were resolved on SDS/PAGE and silver stained, followed by mass spectrometry analysis. The unique peptide numbers and percentage of peptide coverage of the indicated proteins are shown. (I) Whole-cell lysates from HeLa cells were immunoprecipitated and immunoblotted with antibodies against the indicated proteins. (J) Co-immunoprecipitation analysis of the interaction between LAP2α and PARP1 with cellular extracts from U2OS cells stably expressing LAP2α/wt or LAP2α/2RE. (K) Laser micro-IR (50% laser energy) followed by live-cell imaging analysis of DsRed-LAP2α recruitment kinetics in PARP1-knockdwon U2OS cells. Fluorescence intensities in micro-irradiated areas relative to the background signal of the undamaged regions were quantified ($n > 20$). The knockdown effect was examined by immunoblotting. (L) Laser micro-IR (50% laser energy) followed by live-cell imaging analysis of mCherry-CHD1L recruitment kinetics in U2OS cells pre-treated with rucaparib (10 μM, 4 hr) or talazoparib (10 μM, 4 hr). Fluorescence intensities in micro-irradiated areas relative to the background signal of the undamaged regions were quantified ($n > 20$). (M) pLenti-vector, PARP1/wt or PARP1/E988K stably integrated U2OS cells were co-transfected with control siRNA or PARP1 3'UTR siRNA (siPARP1-1) and DsRed-LAP2α, and cellular extracts were collected for immunoblotting analysis with antibodies against the indicated proteins (related to Figure 6B). For LAP2α bands, the upper is the DsRed-LAP2α and the lower is the endogenous LAP2α. (N) Co-immunoprecipitation analysis of the interaction between LAP2α and PARP1 with cellular extracts from U2OS cells stably expressing PARP1/wt or PARP1/E988K. (O) Laser micro-IR (50% laser energy) followed by live-cell imaging analysis of DsRed-RPA1 recruitment kinetics in PARP1-knockdwon U2OS cells. Fluorescence intensities in micro-irradiated areas relative to the background signal of the undamaged regions were quantified ($n > 20$). (P) U2OS-LacO cells expressing mCherry-LacI and PARP1 siRNAs were labelled with EdU for 1 hr before immunostaining and confocal microscopy analysis. The intensity of RPA1 foci in mCherry-LacI and EdU positive cells was quantified and normalized to the nuclear background ($n > 150$). Data are mean ± SDs for (E), (F), (G), (K), (L), (O) and (P) from biological triplicate experiments. $^{**}P < 0.01$; $^{*}P < 0.05$; NS, not significant; Mann-Whitney test for (E) and (P); two-way ANOVA for (F), (G), (K), (L), and (O). Scale bar, 10 μm.
