# Supplementary material for: LAP2α preserves genome integrity through assisting RPA deposition on damaged chromatin
Source: Genome Biol. 2022 Feb 28;23:64. doi: 10.1186/s13059-022-02638-6 (PMC8883701; doi:10.1186/s13059-022-02638-6)
Supplement: Supplementary file 4 — Additional file 4. Supplemental Methods. [file 13059_2022_2638_MOESM4_ESM.pdf]

SUPPLEMENTAL METHODS

Antibodies and reagents

The sources of antibodies against the following proteins or post-translational modifications were as follows: RPA1 (2198S, for IF), γH2AX (9718S, for IF), CDC45 (3673S, for WB), and CHK1 pS345 (2348S, for WB) from Cell Signaling Technology; FLAG (F3165, for IF, WB, and IP) from Sigma; γH2AX (05-636, for IF and WB) from Millipore; LAP2α (ab5162, for WB, IP, and IF), RAD51 (ab133534, for WB), RAD51 (ab63801, for IF), MRE11 (ab214, for WB and IF), RPA2 (ab2175, for IF), and RPA1 (ab176467, for WB and IF) from Abcam; PARP1 (sc-7150, for WB and IP) and HA (sc-805, for WB) from Santa Cruz; RPA2 pS4/8 (A300-245A, for WB), RPA2 pS33 (A300-246A, for WB and IF) from Bethyl Lab; β-actin (AC004, for WB), RPA3 (A6721, for WB) from Abclonal; RPA2 (10412-1-AP, for WB), PCNA (60097-1-Ig, for WB), CHK1 (10362-1-AP, for WB), BRCA1 (22362-1-AP, for WB and IF) and RB1 (10048-2-Ig, for WB) from Proteintech; CHD1L (RLT5027, for WB) and Lamin A/C (RLT2521, for WB) from RuiYingBio; BrdU (RPN202, for IF) from GE healthcare; IdU/BrdU (347580, for DNA fiber IF) from BD Bioscience; CldU/BrdU (MCA2060GA, for DNA fiber IF) from AbD Serotec; H2AX (YT2155, for WB) and H3 (YM3038, for WB) from Immunoway; and LAP2α (IQ175, for WB) from ImmuQuest. Glutathione Sepharose 4B (17-0756-01) was purchased from GE healthcare. Protein G Magnetic Beads (10004D) and Dynabead MyOne Streptavidin T1

(65601) were purchased from Invitrogen. Anti-FLAG M2 affinity gel (A2220), 3 × FLAG peptide (F4799), His-Select Nickel Affinity Gel (P6611), CPT (C9911), HU (H8627), IdU (I7125), CldU (C6891), DNase (D5025), RNase (R6513), DAPI (F6057), blasticidin (15205), puromycin (P8833), neomycin (N1142), 4-OHT (H6278), and doxycycline (D9891) were purchased from Sigma. Velaparib (ABT-888), rucaparib (AZD2281), talazoparib (BMN 673), and cisplatin (S1166) were purchased from Selleck.

Plasmids

The DsRed-LAP2α, GFP-LAP2α, and FLAG-LAP2α were amplified from LAP2α cDNA (Origene) and cloned into the pLenti-Puro-3XFLAG, or pLVX-Tight-Puro-3XFLAG vector, while the FLAG-tagged LAP2α/2RA (R86A, R88A), LAP2α/2RE (R86E, R88E), LAP2α/2RQ (R86Q, R88Q), LAP2α/RK>AA (90-109), LAP2α/PQ>AA (76-109), LAP2α/ST>AA (76-109), and LAP2α/ED>AA (76-109) were chemically synthesized and cloned into the pLenti-Puro-3XFLAG vector. The FLAG tagged LAP2α/wt, LAP2α/N, LAP2α/M, LAP2α/C, LAP2α/ΔC1, LAP2α/ΔC2, LAP2α/ΔC3, LAP2α/ΔN50, LAP2α/ΔN62, LAP2α/ΔN75, LAP2α/ΔN89, LAP2α/ΔN109, LAP2α/ΔN153, LAP2α/Δ76-89, LAP2α/Δ81-91, and LAP2α/Δ90-109 carried by the pLenti-Puro-GFP-3XFLAG vector were generated by standard cloning procedures. The GFP-LAP2β was amplified from LAP2β cDNA (Origene) and cloned into the pLVX-Tight-Puro vector. His tagged LAP2α/wt and LAP2α/2RE were generated into pFastBac HTA vector and used to purify recombinant proteins from insect cells. The DsRed-RPA1, GFP-RPA1, and FLAG-

RPA1 were amplified from RPA1 cDNA (Origene) and cloned into the pLenti-Puro or pLVX-Tight-Puro vector. His tagged RPA2 and RPA3 were carried by the pET-28a vector. The GST tagged RPA DBD-A domain was carried by the pGEX-4T-3 vector. The His-MBP-RPA1 and SFB-tagged wild type and deletion mutants of RPA1 were gifts from Dr. Jun Huang (Life Sciences Institute and Innovation Center for Cell Biology, Zhejiang University, China). The FLAG tagged PARP1/wt was amplified from PARP1 cDNA (Origene) and cloned into the pLenti-Puro-3XFLAG vector, and PARP1/E988K was generated by quick-change point mutation assays. The mCherry-CHD1L was a gift from Dr. Sébastien Huet (Institut Génétique et Développement de Rennes, France gyula). p11d-tRPA(1-2-3) was a gift from Dr. Marc Wold (Addgene plasmid # 102613). CRISPR/Cas9 constructs lentiCas9-Blast (Addgene plasmid # 52962) and lentiGuide-Puro (Addgene plasmid # 52963) were gifts from Dr. Feng Zhang (Broad Institute).

Cell culture

U2OS, HEK 293T, and HeLa cells were got from the American Type Culture Collection (Manassas, VA) and cultured under the manufacturer's instructions. The LacO-LacI U2OS cells were kindly provided by Dr. Roger Greenberg (University of Pennsylvania, Pennsylvania). Cells that allow protein expression under doxycycline treatment were created in two steps. First, cells were infected with lentivirus carrying rtTA and subjected to neomycin or blasticidin selection. Subsequently, the established rtTA cells were infected with a virus carrying pLenti-Tight-Puro vector that encodes RPA1, LAP2α/wt,

LAP2α/2RE or LAP2β followed by puromycin selection. Cells integrated with rtTA were cultured in Tet Approved FBS and medium from Clontech. All of the cells were authenticated by examination of morphology and growth characteristics and confirmed to be mycoplasma free.

Generation of mouse embryonic fibroblast cells (MEFs)

Lap2α conditional knockout mice were established by Dr. Roland Foisner's lab at Medical University of Vienna and University of Vienna, Austria. Neo selection marker was removed in our hands. Embryos were harvested under sterile conditions from a pregnant mouse on day E13.5 post-fertilization. After removing the liver and heads of each embryo, the remaining embryonic tissue was dissected into small pieces using a scalpel in PBS and homogenized into small pieces of approximate 1 mm^3 by additionally mincing. Then the tissue pellet was placed into a 15 mL tube containing 1 × trypsin-EDTA (0.25% trypsin/2.21 mM EDTA in Hank's Balanced Salt Solution without sodium bicarbonate, calcium, and magnesium; Cellgro) inside a sterile tissue culture hood and incubated for 1 hr at 37 °C incubator. Then, excess trypsin was drained from each 15 mL tube before adding 2 mL DNase I (2000 U/mL, New England Biolabs) to the embryo and incubating at 37 °C for 5 min. Following the incubation, primary MEF media DMEM (Gibco) supplemented with 10% fetal bovine serum, 100 μg/mL penicillin/streptomycin, 2 mM L-glutamine, 1 × nonessential amino acids (Gibco), 1.25 μg/mL Plasmocin (InvivoGen), and 0.1 mM 2-mercaptoethanol was used to inactivate the trypsin and break the embryos into a single cell

suspension. Finally, the cells were cultured on a 10 cm plate coated with 0.2% gelatin.

Generation of Lamin A/C knockout U2OS cells

Lamin A/C knockout cells were generated by co-transfection of plasmid encoding FLAG-Cas9 (lentiCas9-Blast) and sgRNA plasmid (lentiGuide-Puro) targeting *LMNA* (sgRNA-1: TTCCGCCAGCAGCCGCCGGC, sgRNA-2: AGCGGGAGATGGCCGAGATG). 48 hr after transfection, cells were selected by blasticidin (5 μg/ml) and puromycin (1 μg/ml) for 2 days. Pooled clones were used in the following experiments.

Recombinant protein purification and pull-down assays

Recombinant baculovirus carrying His-MBP-RPA1, His-LAP2α/wt, and His-LAP2α/2RE (R86E, R88E) were generated with the Bac-to-Bac System (Thermo Fisher). Infected Sf9 cells were grown in spinner culture for 48 to 96 hr at 27 ºC and lysed by ultrasonicator in Equilibration buffer (50 mM sodium phosphate, 0.3 M sodium chloride, 10 mM imidazole, and 10 mM Tris-HCl, pH 8.0). His-RPA2, His-RPA3, GST, and GST-DBD-A were expressed in Escherichia coli cells. His tagged proteins were purified using Ni-NTA agarose (Thermo Fisher) according to standard procedures. GST-fusion proteins were purified by glutathione-Sepharose 4B beads (GE Health Care) and then washed with high salt buffer (20 mM Tris-HCl (pH 7.4), 0.1 mM EDTA, and 300 mM NaCl). For GST pull-down assay, GST-fusion proteins were incubated with His-tagged proteins at 4°C overnight. The beads were washed 4 times, then boiled in SDS loading buffer and subjected to SDS-

PAGE followed by CBB staining or immunoblotting.

RPA purification

p11d-tRPA encoding RPA1, RPA2 and RPA3 was used to transform BL21(DE3) cells. One liter cultures were then inoculated with a single colony of freshly transformed cells and incubated at 37 ºC overnight without aeration or shaking. The next morning, an OD at 600 nm was taken, and then the cells were shaken (200-250 rpm) at 37 ºC until they reached an OD 600 of 0.5-0.8. Protein expression was induced by the addition of isopropyl-β-D-thiogalactopyranoside (IPTG) to a final concentration of 0.3 mM, and incubated with shaking for 2-3 hr. The cells were pelleted by centrifugation at 4000 rpm at 4 ºC, and were re-suspended in 150 mL T-buffer (25 mM Tris-HCl pH 7.5, 0.5 mM EDTA, 0.25% myo-inositol, 1mM DTT, 0.01 % NP40) with 1 mM PMSF, 100 mM KCl. Then cells were processed 3 times with a pressure limit of 1100 psi by French press. The lysed solution was cleared by centrifugation (16,000 g for 1 hr) and loaded on Affi-gel blue resin (5 mL, BioRad) with T-buffer containing 100 mM KCl. Affi-gel blue resin was washed by 20 mL of T-buffer containing 100 mM KCl, followed by 20 mL of T-buffer containing 800 mM KCl. Protein was eluted from resin by a NaSCN gradient from 0.5 M to 1.5 M (20 mL). Eluted fractions containing RPA were pooled together and dialyzed against T-buffer with 50 mM KCl. After dialysis, RPA fractions were loaded onto an Mono Q column (5/50 GL, GE Healthcare). The column was washed with 10 mL of T-buffer with 50 mM KCl and eluted with a KCl gradient from 50 to 500 mM. Fractions containing recombinant RPA

were collected and stored at -80 °C.

Immunoprecipitation

Cell lysates were prepared by incubating the cells in NETN buffer (50 mM Tris-HCl, pH 8.0, 150 mM NaCl, 0.2% Nonidet P-40, 2 mM EDTA) in the presence of protease inhibitor Cocktails (Roche) for 20 min at 4 ºC. This was followed by centrifugation at 14,000 g for 15 min at 4 ºC. For immunoprecipitation, about 500 μg of protein was incubated with control or specific antibodies (1-2 μg) for 12 hr at 4 ºC with constant rotation; 50 μl of 50% protein G magnetic beads (Invitrogen) was then added and the incubation was continued for an additional 2 hr. Beads were then washed five times using the lysis buffer. Between washes, the beads were collected by a magnetic stand (Invitrogen) at 4 ºC. The precipitated proteins were eluted from the beads by re-suspending the beads in 2 × SDS-PAGE loading buffer and boiling for 5 min. The boiled immune complexes were subjected to SDS-PAGE followed by immunoblotting with appropriate antibodies.

Biolayer interferometry (BLI) assay

The dose-dependent binding affinities of LAP2α for RPA were determined by a biolayer interferometry assay using Octet RED96 (ForteBio). AR2G (Amine Reactive 2G) biosensor tips (ForteBio, Menlo Park, CA) were used to immobilize RPA after prewetting with kinetic buffer (PBS, 0.05%; bovine serum albumin, 0.01% Tween 20). The equilibrated biosensors were loaded with RPA (10 μg/ml) for 5 min. Background binding

controls used a duplicate set of sensors that incubated in buffer without proteins. Assays were performed by a standard protocol in 96-well black plates with a total volume of 200 μl per well at 28°C. The signals were analyzed by a double reference subtraction protocol to deduce nonspecific and background signals and signal drifts caused by biosensor variability. A 1:1 binding model was used to fit the association and dissociation rates. Equilibrium dissociation constant (K_D) values were calculated from the ratio of K_{off} to K_{on}.

Stable isotope labeling with amino acids in cell culture (SILAC)

The cells were grown to 80% confluence in high glucose (4.5 g/liter) Dulbecco's modified Eagle's medium (with glutamine and sodium pyruvate) containing 10% fetal bovine serum and 1% penicillin-streptomycin at 37 °C with 95% air and 5% CO_2. Control cells were labeled with "heavy isotopic lysine and arginine" (K6R10), and the cells of doxycycline (Dox) treatment were labeled with "light isotopic lysine and arginine" (K0R0) using a SILAC Protein Quantitation Kit (Pierce, Thermo) according to the manufacturer's instructions. After labelling, cells grown in each SILAC medium were harvested separately by scraping in PBS and the cell pellets were lysed in NETN buffer in the presence of protease inhibitor Cocktails (Roche) for 30 min at 4 ºC. Cellular extracts from an equal amount of cells (about 1×10^8 cells) under each treatment were applied to an equilibrated FLAG column of 1 mL bed volume to allow for adsorption of the protein complex to the column resin. After binding, the column was washed with cold PBS plus 0.2% Nonidet P-40. FLAG peptide was applied to the column to elute the FLAG protein complex as

described by the vendor. The eluents were subjected to NuPAGE 4-12% Bis-Tris gel (Invitrogen) until all proteins were fully loaded onto the gel, and samples from different labelling were collected from the gel. The gel pieces were mixed and digested with trypsin followed by standard LC-MS/MS Analysis. The resulting MS/MS data were processed using Maxquant (v1.5.2.8) with the following parameters: trypsin, 2 max missed cleavages, minimum peptide length 7, maximum number of modifications per peptide 5. Tandem mass spectra were searched against the SwissProt human database. The mass error of first search was set to 20 ppm for precursor ions. The mass error of main search was set to 5 ppm for precursor ions and 0.02 Da for fragment ions. Carbamidomethyl on Cys was specified as a fixed modification, oxidation on Met, and acetylation on protein N-terminal were specified as variable modification. Peptide confidence was set at high, and peptide ion score was set > 20. The fold change of protein expression was calculated from the ratio of protein intensity between L and H. And protein intensity was the sum of corresponding unique peptide intensity. Two biological replicate experiments were performed. The PSMs and peptides were filtered to 1% PSM FDR, and the protein groups were filtered to 1% protein FDR.

Immunopurification and silver staining

Lysates from HeLa cells stably expressing FLAG-LAP2α were prepared by incubating the cells in lysis buffer containing protease inhibitor cocktail (Roche). Anti-FLAG immunoaffinity columns were prepared using anti-FLAG M2 affinity gel (Sigma)

following the manufacturer's suggestions. Cell lysates were obtained from about 5×10^7 cells and applied to an equilibrated FLAG column of 1 ml bed volume to allow for adsorption of the protein complex to the column resin. After binding, the column was washed with cold PBS plus 0.2% Nonidet P-40. FLAG peptide (Sigma) was applied to the column to elute the FLAG protein complex as described by the vendor. The elutes were collected and visualized on NuPAGE 4-12% Bis-Tris gel (Invitrogen) followed by silver staining with silver staining kit (Pierce). The distinct protein bands were retrieved and analyzed by LC-MS/MS.

Nano-HPLC-MS/MS analysis

The proteins were separated by SDS-PAGE and visualized by silver staining. Then, the corresponding bands were excised and subject to in-gel digestion. The resulting peptides were redissolved in HPLC buffer A (0.1% formic acid in water) following desalting and injected into a Nano-LC system (EASY-nLC 1000, Thermo Fisher Scientific). Peptides were separated using a reversed-phase analytical column and electrosprayed directly into an Orbitrap Q-Exactive mass spectrometer. The mass spectrometric analysis was carried out in a data dependent mode with an automatic switch between a full MS scan and an MS/MS scan in the orbitrap. For full MS survey scan, automatic gain control (AGC) target was 1e6, scan range was from 350 to 1750 with the resolution of 70, 000. The 10 most intense peaks with charge state 2 and above were selected for fragmentation by higher-energy collision dissociation (HCD) with normalized collision energy of 27%. The MS2

spectra were acquired with 17,500 resolution. The exclusion duration for the data-dependent scan was 10 sec, and the exclusion window was set at 2.2 Da. The resulting MS/MS data were searched using Proteome Discoverer software (v1.4) with an overall false discovery rate (FDR) for peptides of less than 1%. Proteins demonstrating the score < 2 and single-peptide identifications were removed from identification list. Peptide sequences were searched against Uniprot human (20,386 entries, download at 2021.06.23) database using trypsin specificity and allowing a maximum of two missed cleavages. Carbamidomethylation on Cysteine was specified as fixed modification. Oxidation of methionine and acetylation on peptide N-terminal were set as variable modifications. Mass tolerances for precursor ions were set at ±10 ppm for precursor ions and ±0.02 Da for MS/MS.

RNA interference

All siRNA transfections were performed using Lipofectamine RNAi MAX (Invitrogen) following the manufacturer's recommendations. The final concentration of the siRNA molecules is 10 nM and cells were harvested 72 or 96 hr later according to the purposes of the experiments. Control siRNA (ON-TARGETplus Non-Targeting Pool, D-001810-10) and CDC45 siRNA (ON-TARGETplus, L-003232) were got from Dharmacon in a smart pool manner, while siRNAs of LAP2α (siRNA-1: GUCUAGAAGUGGCUAAGCA, siRNA-2 targeting 3’ UTR: GCUUUCUAGAUCACAUAUU), LAP2β (siRNA-1: GGGAUAUUCUUAAGGAAAU, siRNA-2: GGUGGAAACUUCAGAACAU), LAP2

(5'UTR siRNA: CUGCUCGCCUCCUGCCUGU), RPA1 (siRNA-1: GAGAAUCAGUGGGUGACUU, siRNA-2: GUGAACAAGGUGUAUUAUU), CtIP (siRNA: UCCACAACAUAAUCCUAAU), and PARP1 (siRNA-1 targeting 3'UTR: GAGAGAUUCUGUUGCAUAG, siRNA-2 targeting 3'UTR: CGAAGCGCUUCUGCACCAA) were chemically synthesized by Sigma (Shanghai, China).

High-content microscopy

After siRNA transfection for 72 hr in 96-wells plates, cells were treated with HU (2 mM, 4 hr) or CPT (1 μM, 2hr) and labelled with EdU for 1 h at 37 ºC. Next, cells were washed twice with cold PBS, extracted with 0.5% Triton X-100 for 5 min on ice and fixed with 3% paraformaldehyde and 2% sucrose for 15 min at room temperature. Cells were then permeabilized with 0.5% Triton X-100 for 5 min on ice, followed by incubation in blocking buffer (0.1% Triton X-100, 5% donkey serum in PBS) for 1 hr at room temperature. Cells were then stained with anti-RPA1 and anti-RPA2 antibody at 4 °C overnight and secondary antibodies coupled to Alexa Fluor 594 and Alexa Fluor 488 (Invitrogen) for 1 hr at room temperature. Incorporated EdU was click-labelled by using keyFluor 647-azide (Keygen Technologies) according to the manufacturer's instructions. Individual wells were then mounted in PBS buffer and the plates were loaded into a PerkinElmer Operetta CLS screening microscope equipped with a 60 × water-immersion objective. Multiple fields of view with triple signal channels per field were collected per well of a 96-wells plate,

corresponding to an average of 10~20 nuclei per field. Approximately 2000 to 4000 nuclei were analyzed per condition. Image segmentation was performed with the Harmony software. For every field of view collected, RPA1 and RPA2 foci were defined and measured using image data captured by the CCD (charge-coupled device) cameras dedicated to the 594 nm and 488 nm laser light sources. Partial nuclei were discarded from the analysis. RPA1 and RPA2 foci, restricted to EdU-positive stained cell nuclear zones, were detected using a spot finding algorithm, and the intensity was autonomously quantified.

X-ray irradiation and laser micro-irradiation

IR was delivered by an X-ray generator (Radsource Corporation RS2000 PRO, 160 kV, 25 mA). Micro-irradiation was performed with a microscope (Leica) equipped with a 37 ºC heating stage and a 365-nm laser diode (Andor Technology). 35% or 50% of full power of laser setting was chosen to generate a detectable laser path under 60 × objective lens.

Laser microdissection

Cells were grown on LabTek II chamber-slides (Thermo Scientific) in the presence of phenol red-free medium (Invitrogen) before induction of DNA damage by a UV-A laser (λ=355 nm, 40% energy) using a Zeiss Observer.Z1 inverted microscope with a PALM MicroBeam laser microdissection workstation under 40 × objective lens. After irradiation, the cells were incubated at 37 ºC for a while and processed for immunostaining.

Comet assay

The Comet Assay kit (Trevigen, Gaithersburg, MD, USA) was used to monitor damaged DNA according to the manufacturer's instructions. Briefly, cells were re-suspended in ice-cold PBS to a concentration of 1×10^5 cells/ml. 5 μl of cells were mixed with 50 μl of warm low melting agarose and evenly spread onto the special comet slides. Slides were then stored in a pre-chilled lysis solution for 60 min at 4 °C. Next, the alkali unwinding solution was used to treat slides. After 60 min incubation at room temperature, slides were transferred to an electrophoresis tank, which contained a pre-chilled alkaline electrophoresis solution, and the system was set to run at 1 volt/cm, 300 mA for 30 min at 4 °C. The slides were immersed twice in deionized water for 5 min intervals and washed in 70% ethanol for 5 min. Then, cells were stained with 100 μl of propidium iodide for 20 min in the dark and analyzed under Olympus IX71 inverted fluorescence microscope. Comet tails were analyzed using Casplab software followed by statistical analysis.

Flow cytometry analysis

MEF cells were treated with 2 mM HU for 24 hr. Cells were washed and fresh medium was added. Cells were harvested every 6 hr after addition of fresh medium for 24 hr. For each sample, 10^6 cells were fixed in cold ethanol, treated with RNase A, stained with propidium iodide and analysed on a BD Biosciences FACSCalibur. To analyze the cell-cycle distribution, cells were treated with 10 μM EdU for 1 hr and labelled with a Click-iT

EdU Alexa Fluor 647 Flow Cytometry Assay kit according to the manufacturer's instructions (Keygen Technologies).

Cell survival assay

Cells were plated into 96-well plates at densities of 2000 cells/well. 24 hr later, cells were treated with various doses of genotoxic agents for 72 hr. Then, Cell Titer Aqueous One Solution Reagent (G3582, Promega) was added to each well according to the manufacturer’s instructions and cell viability was determined after 1 hr incubation by measuring the absorbance at 490 nm using a 550 BioRad plate-reader (Bio-Rad, Hertfordshire, UK).

HR reporter assay

HR efficiency was examined with DR-GFP U2OS cells, in which two incomplete copies of GFP genes are integrated into chromosomal DNA and cleavage of the I-SceI sites leads to the restoration of GFP gene through HR. Percentage of GFP positive cells was counted by flow cytometry analysis with Accuri C6 (BD Biosciences). For each treatment, a minimum of 10,000 cells were collected and analyzed with Flowjo software.
