# Supplementary material for: LAP2α preserves genome integrity through assisting RPA deposition on damaged chromatin
Source: Genome Biol. 2022 Feb 28;23:64. doi: 10.1186/s13059-022-02638-6 (PMC8883701; doi:10.1186/s13059-022-02638-6)

Full unedited gel for Figure 1C

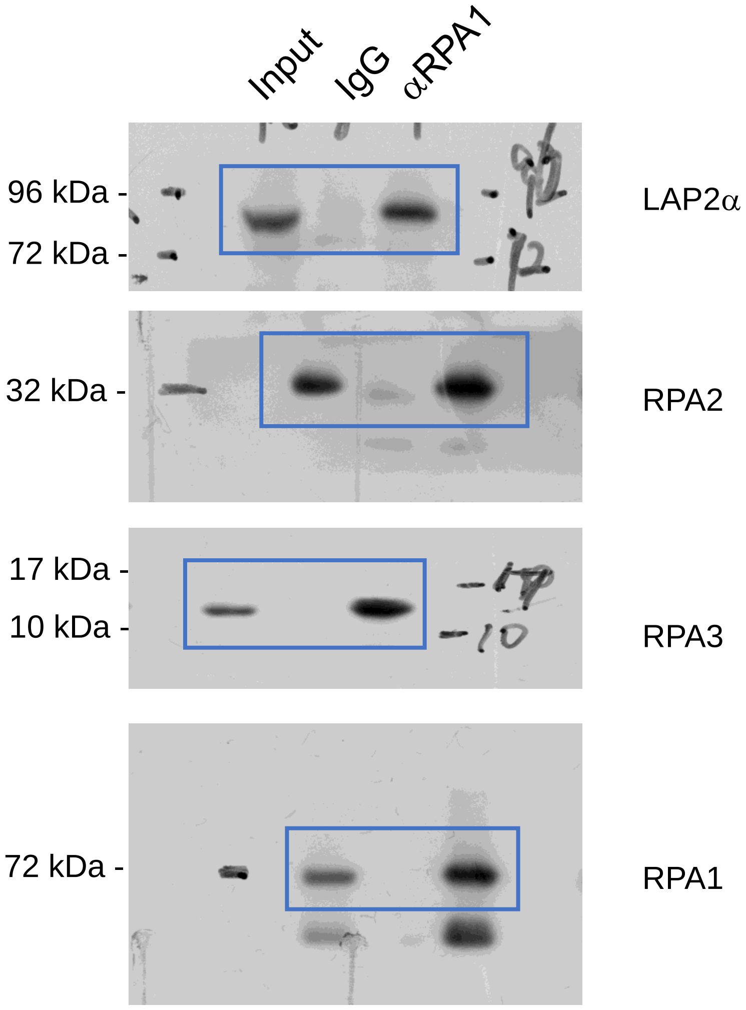

Full unedited gel for Figure 1C

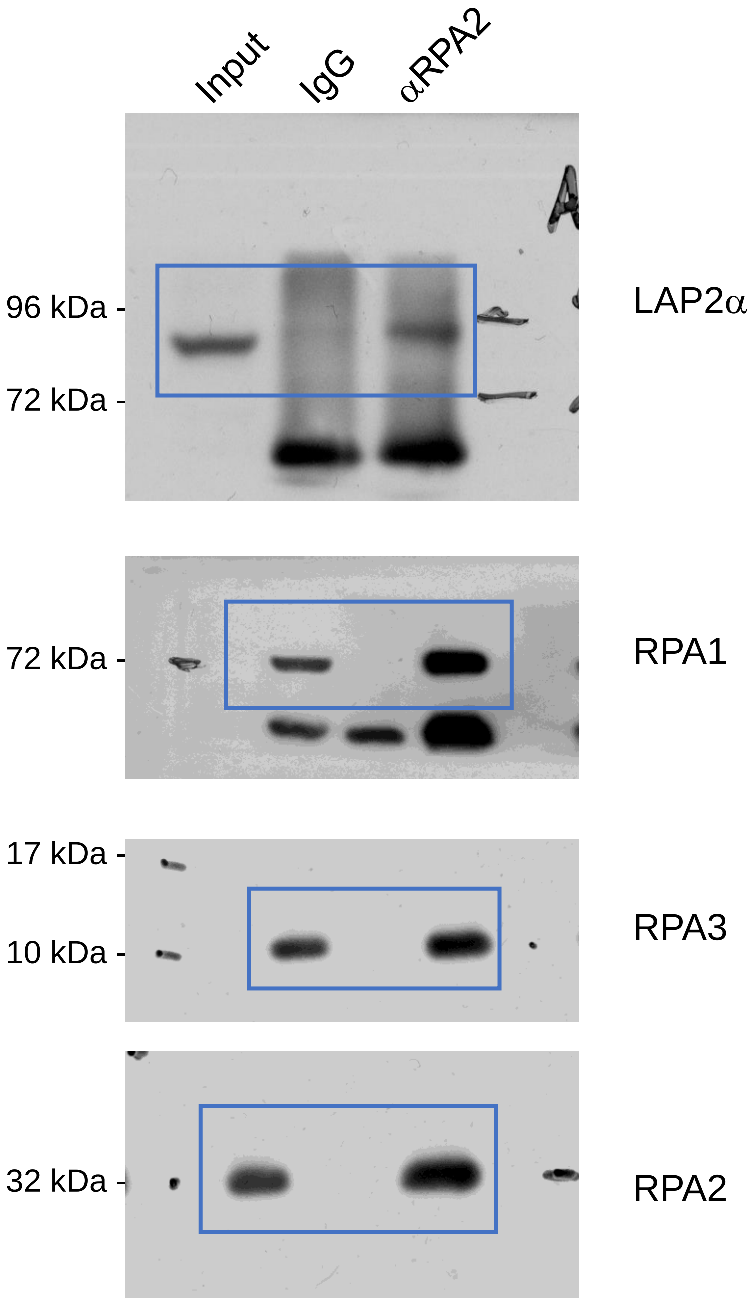

Full unedited gel for Figure 1C

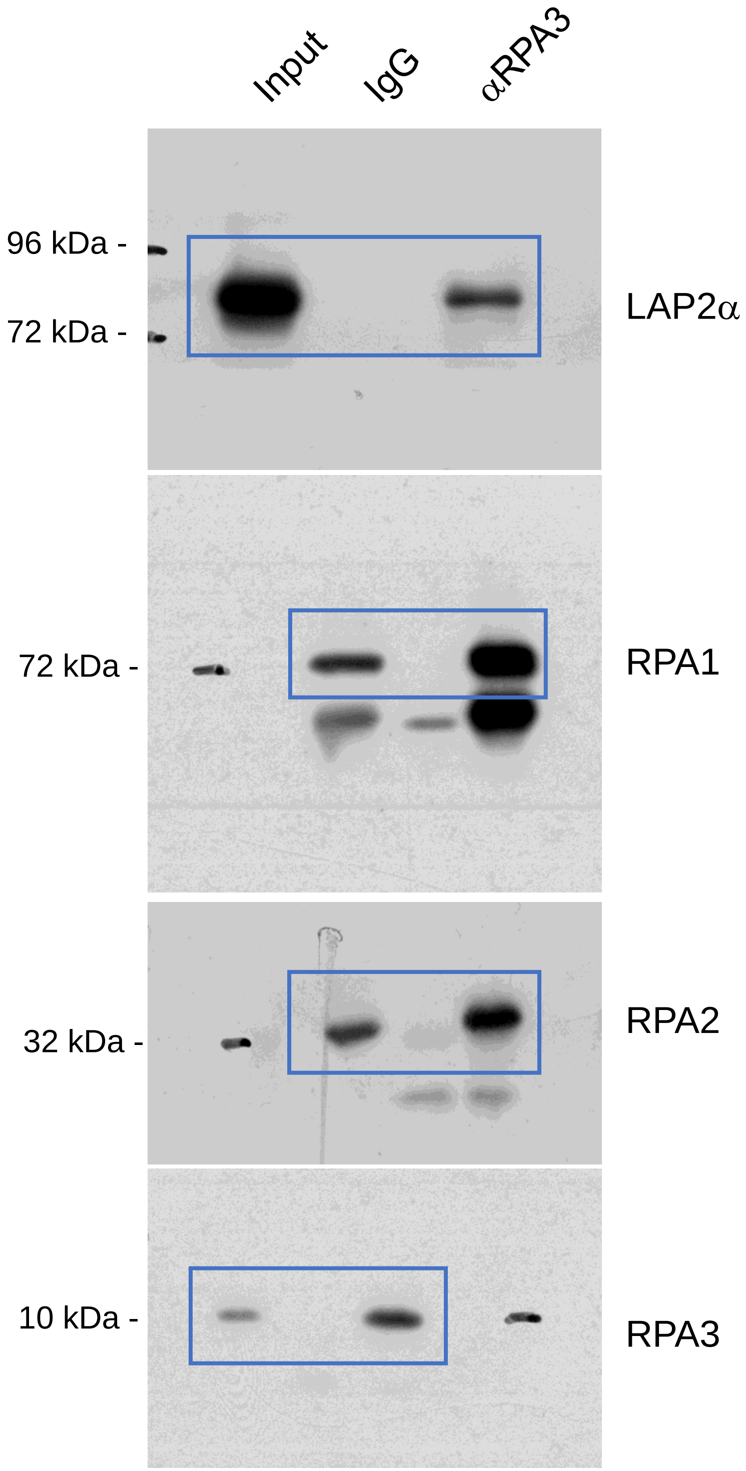

Full unedited gel for Figure 1C

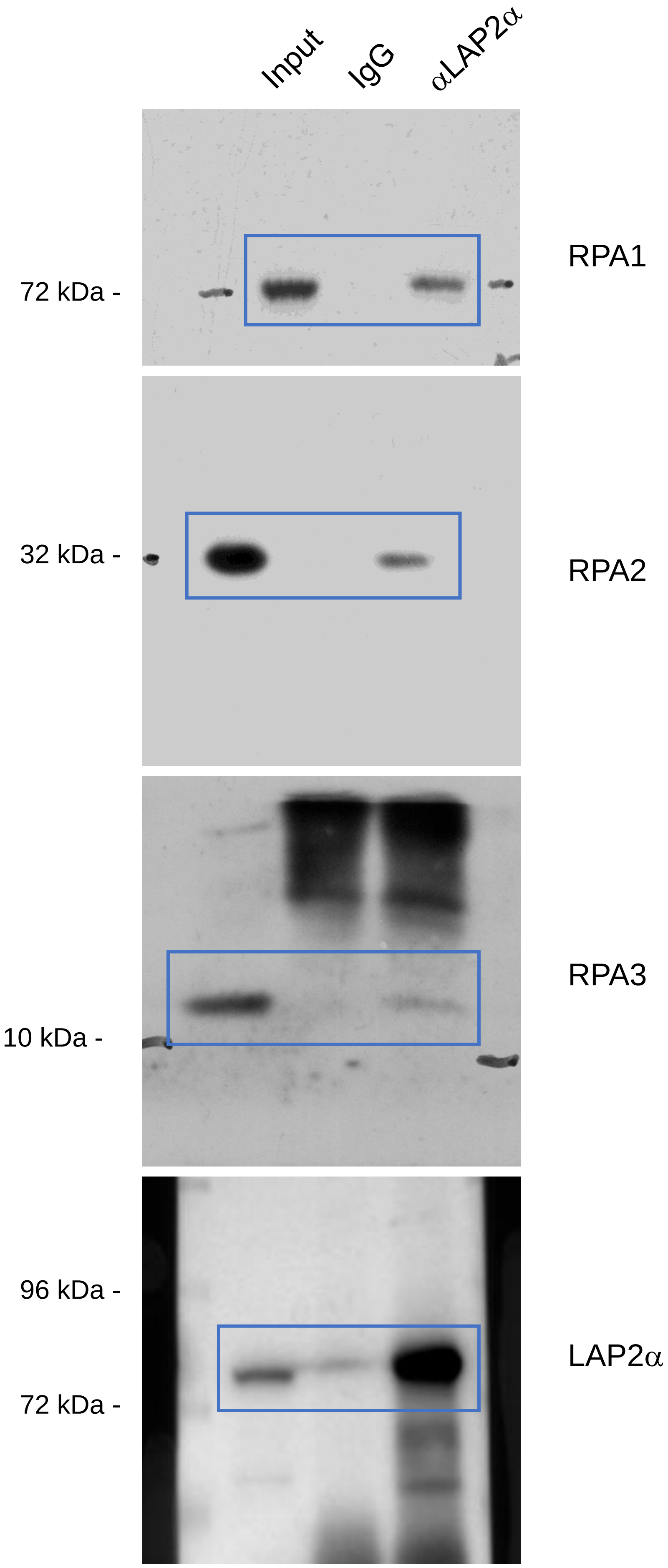

Full unedited gel for Figure 1D

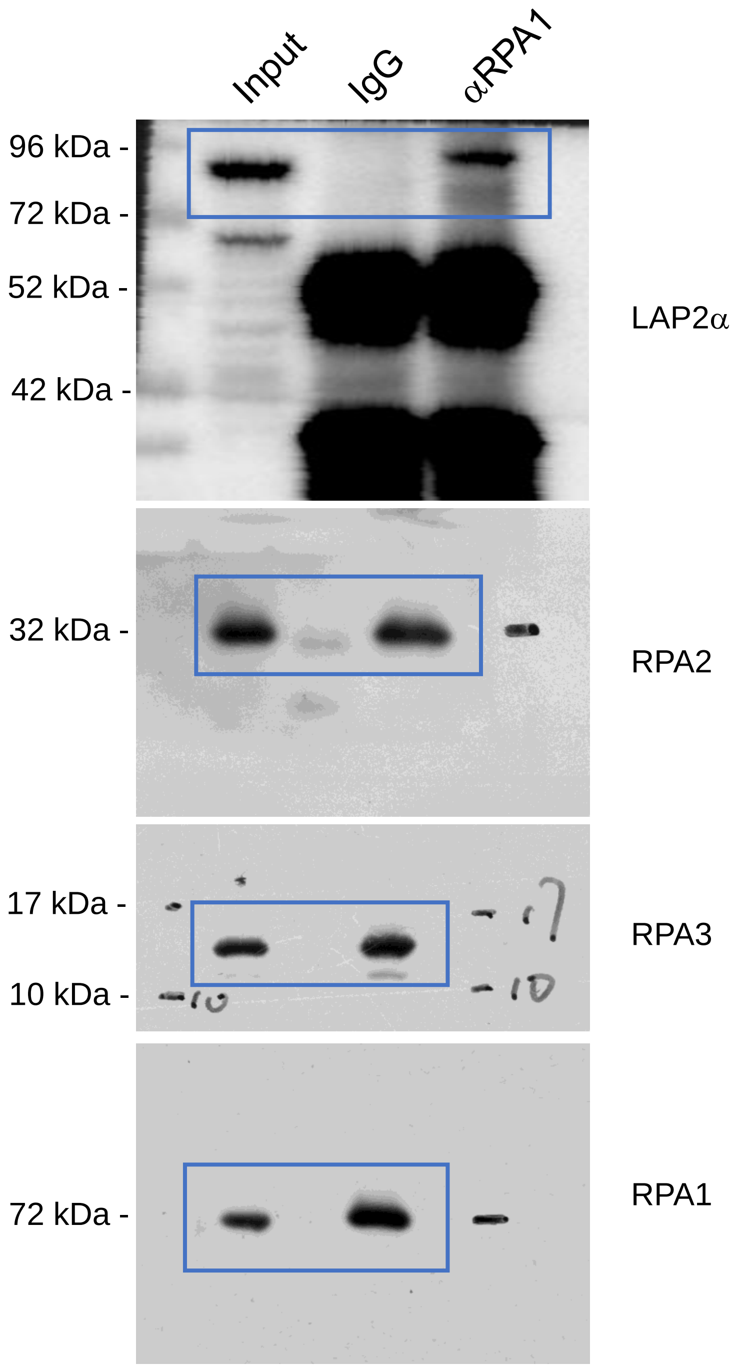

Full unedited gel for Figure 1D

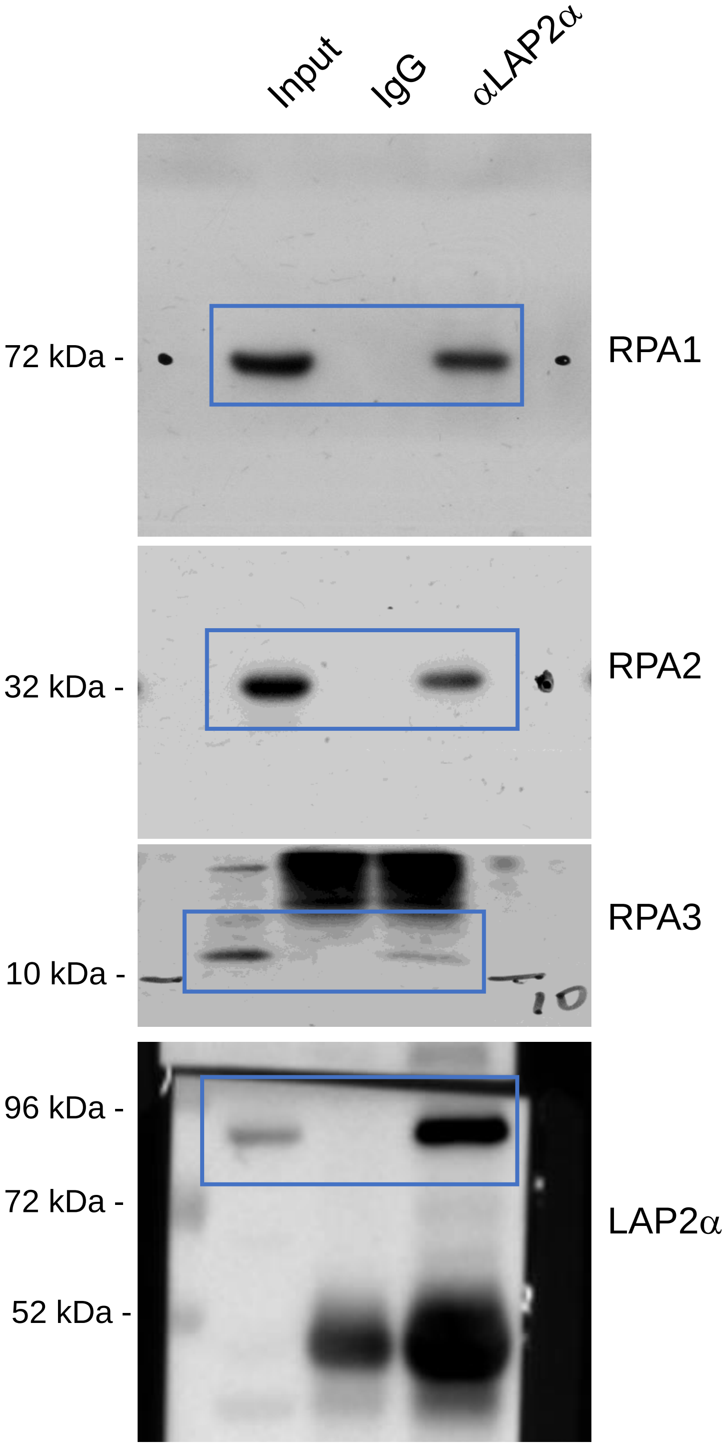

Full unedited gel for Figure 1D

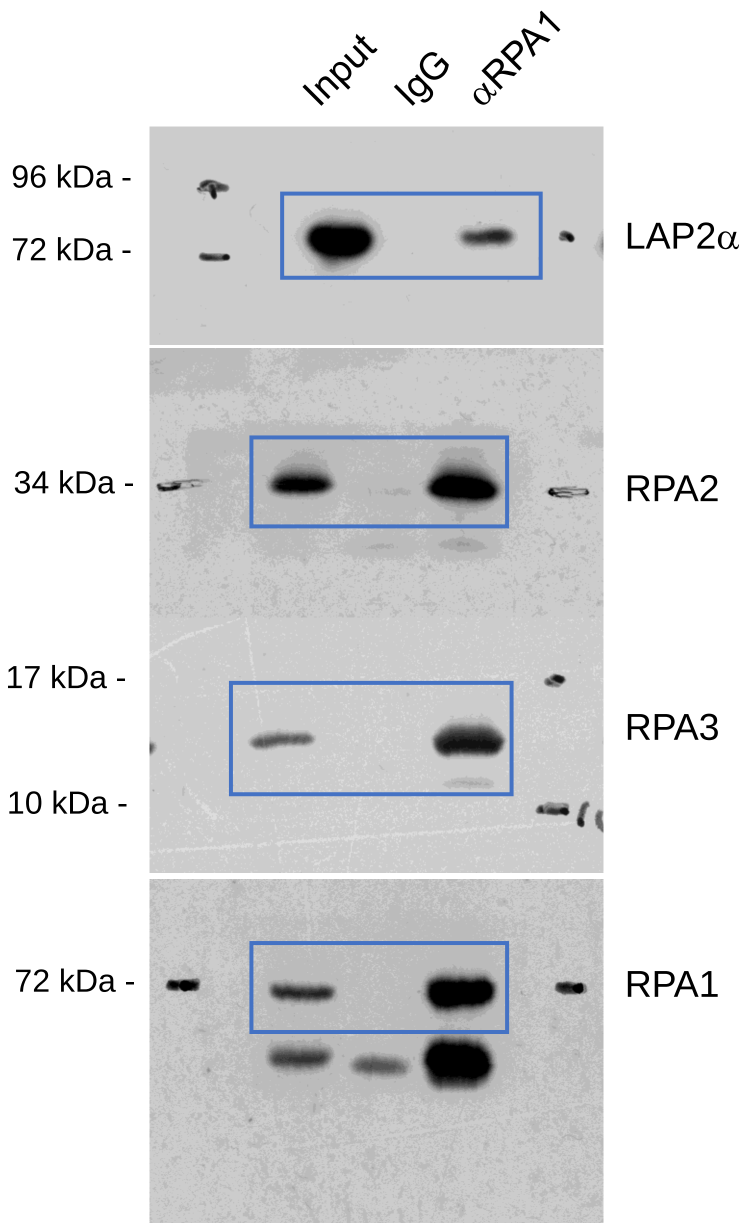

Full unedited gel for Figure 1D

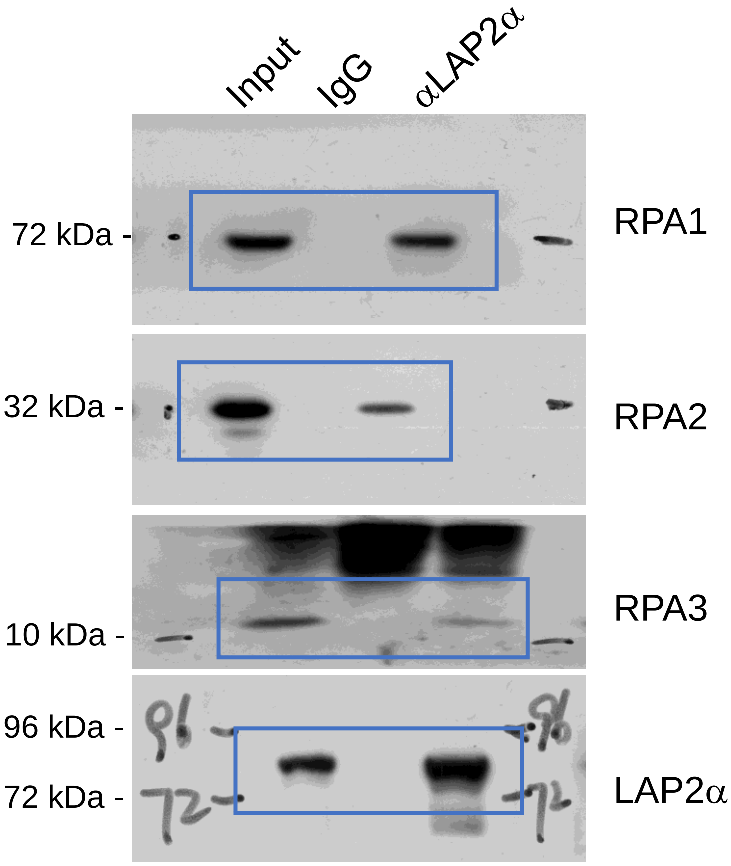

Full unedited gel for Figure 1F

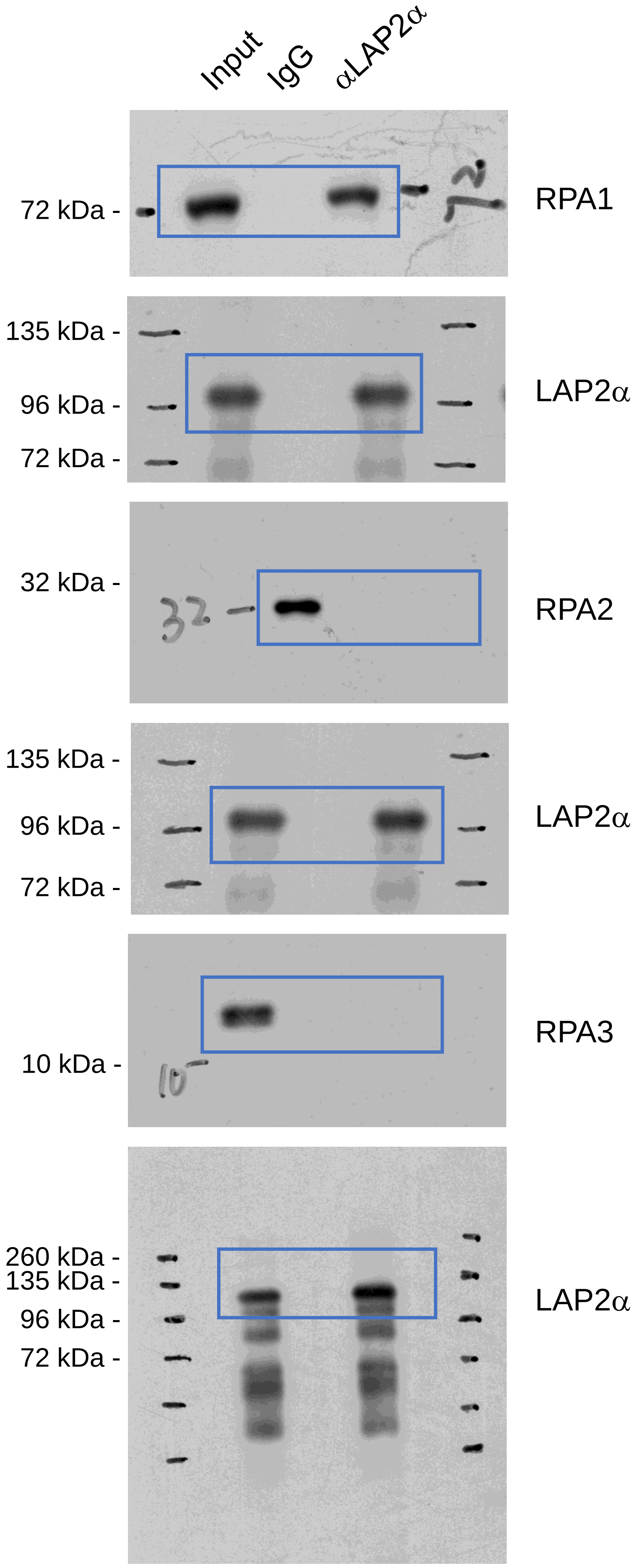

Full unedited gel for Figure 1G

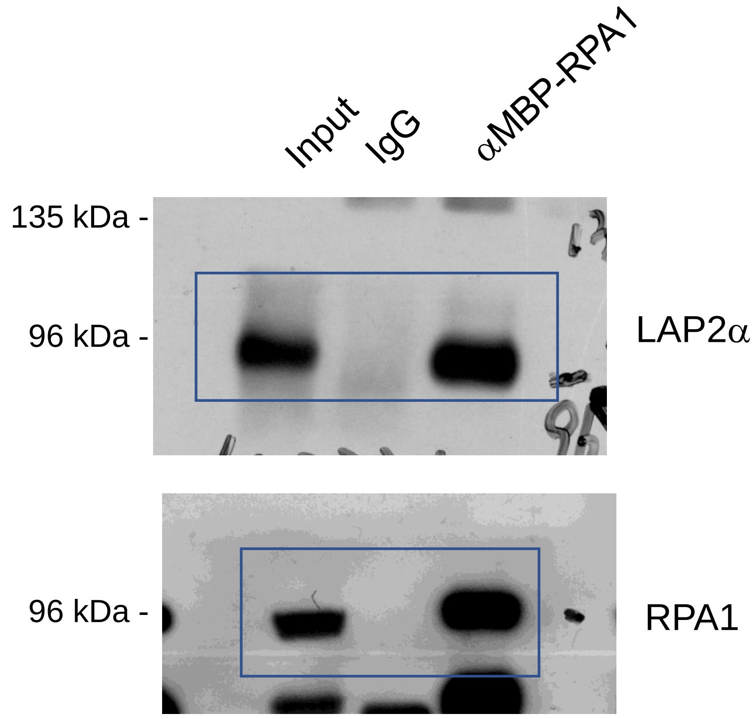

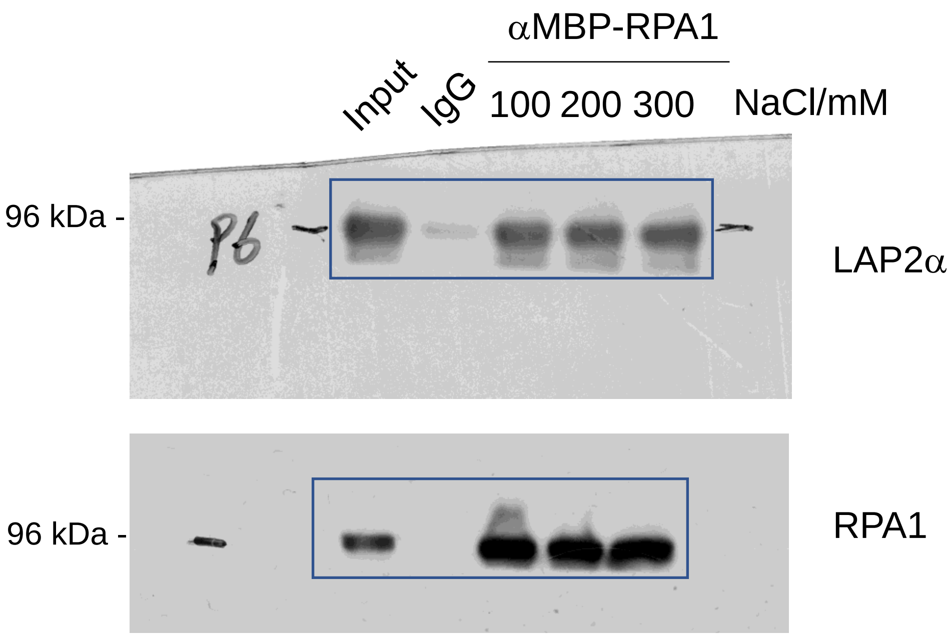

Full unedited gel for Figure 2A

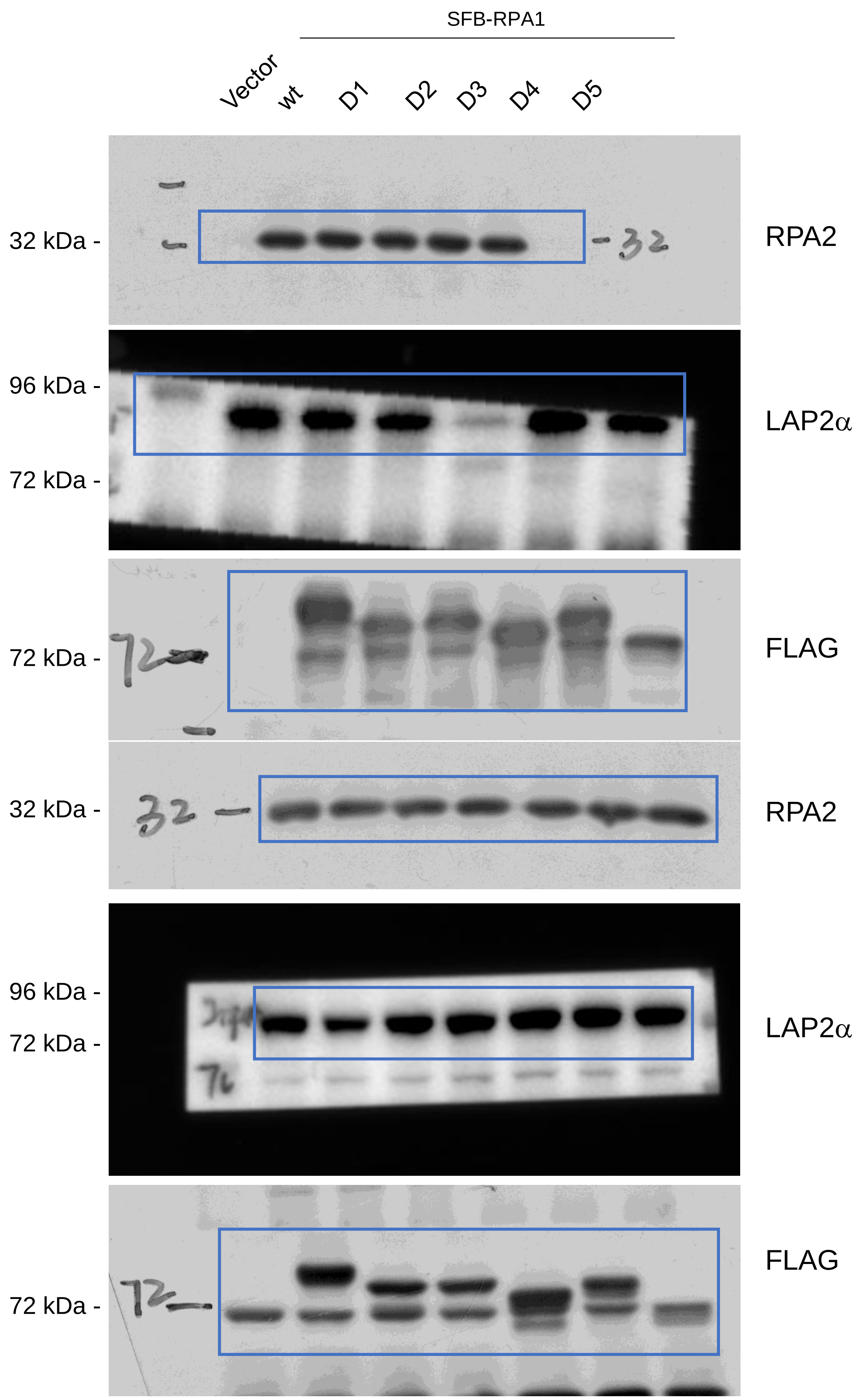

Full unedited gel for Figure 2D

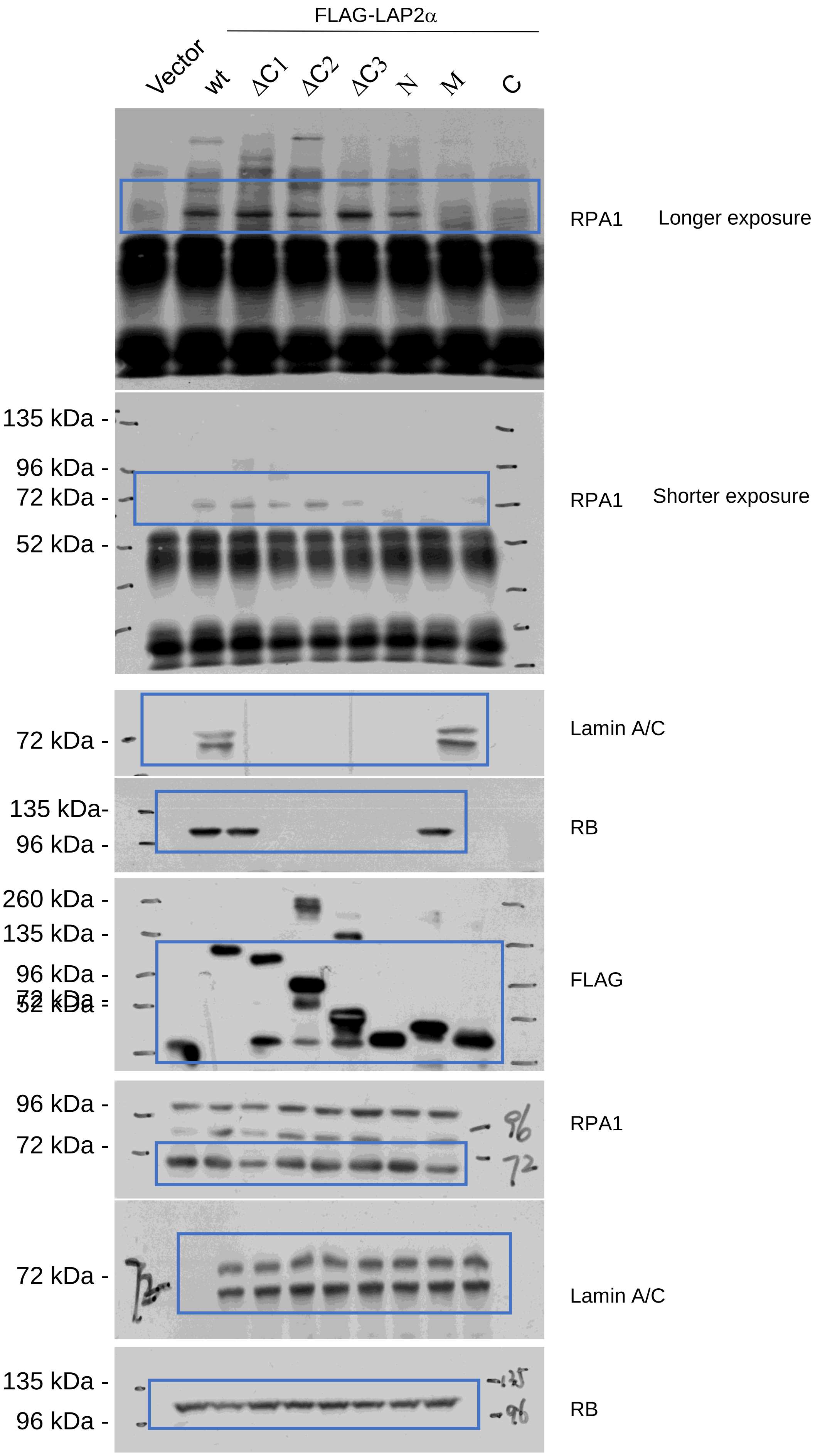

Full unedited gel for Figure 2E

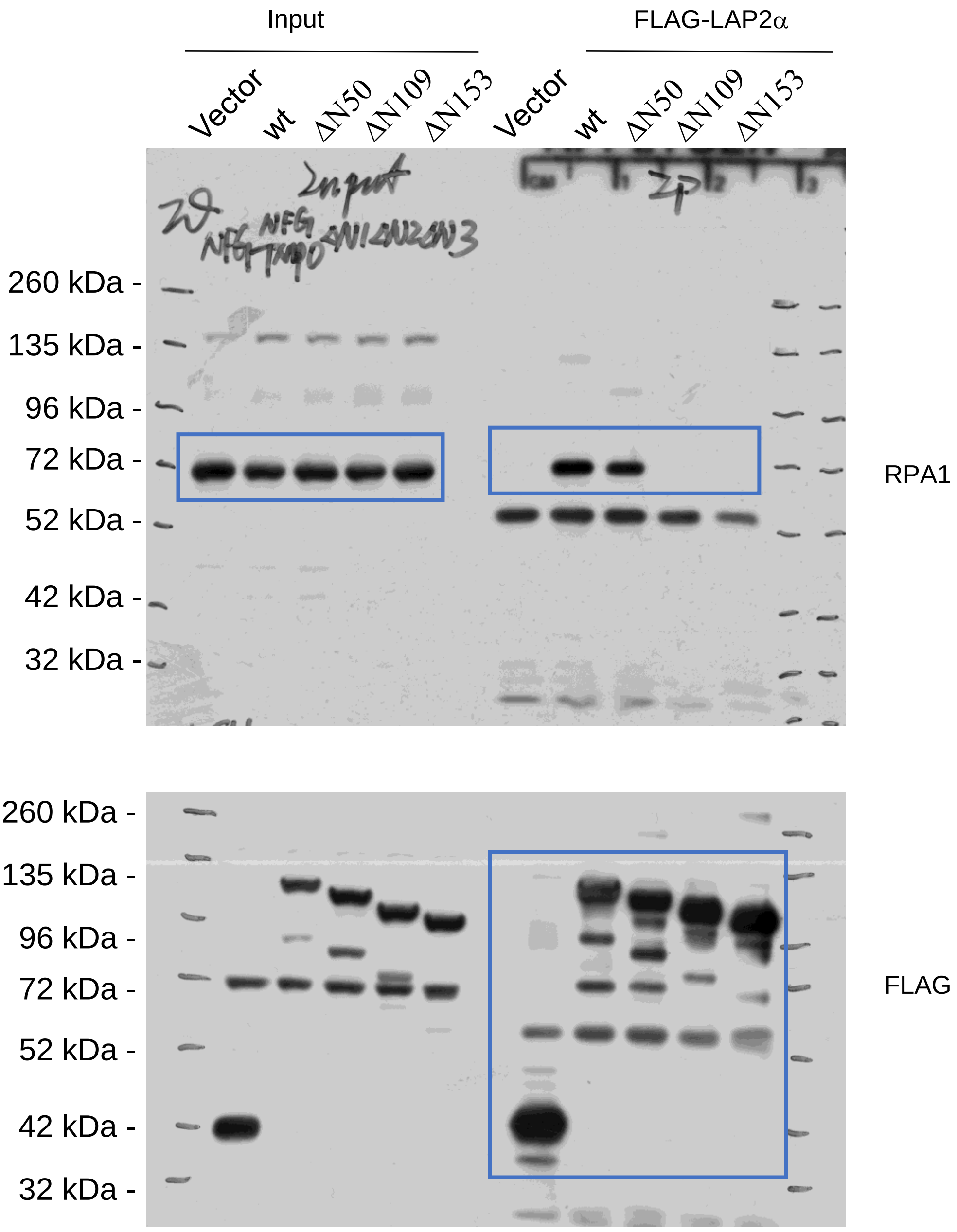

Full unedited gel for Figure 2F

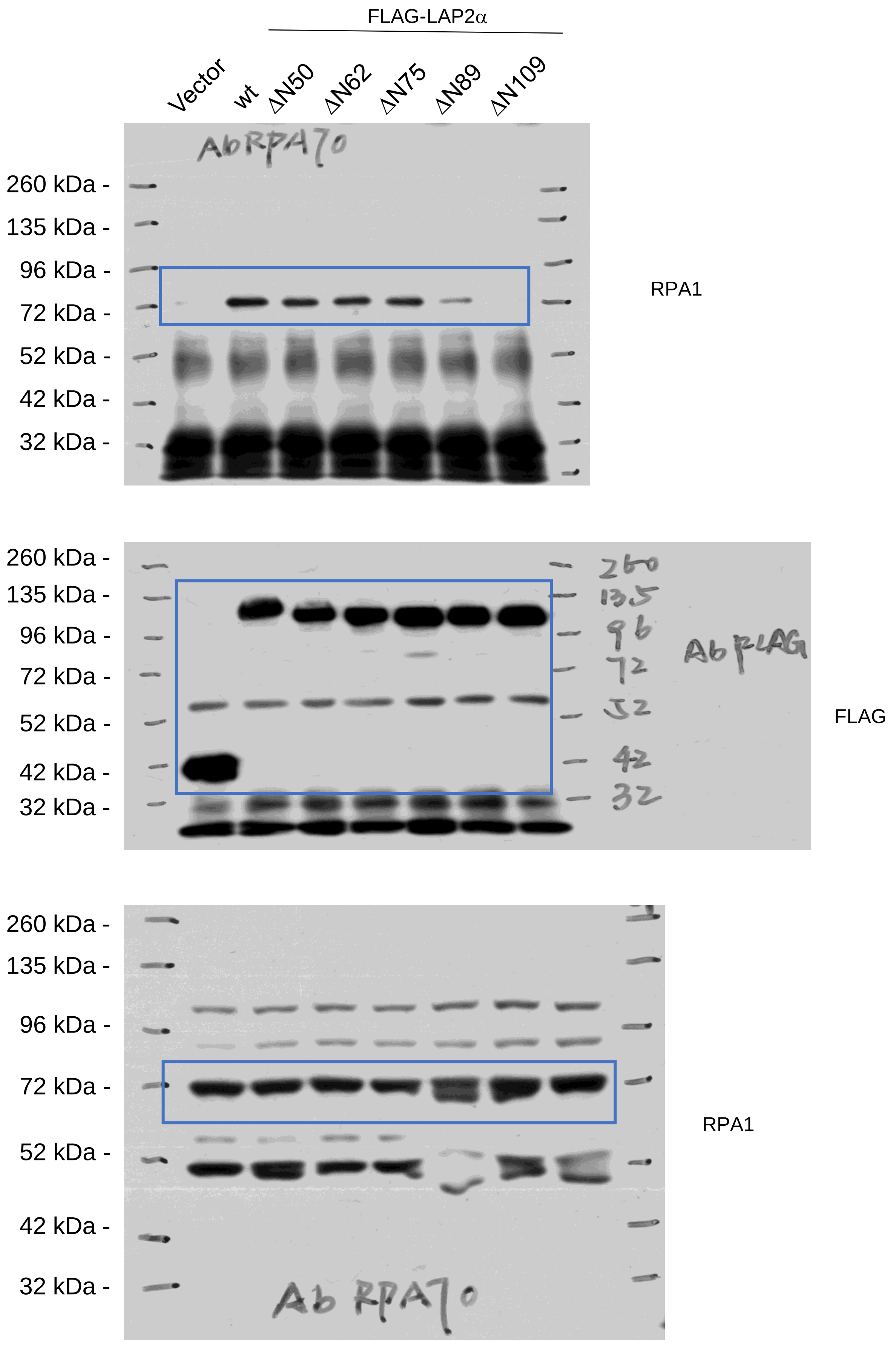

Full unedited gel for Figure 2G

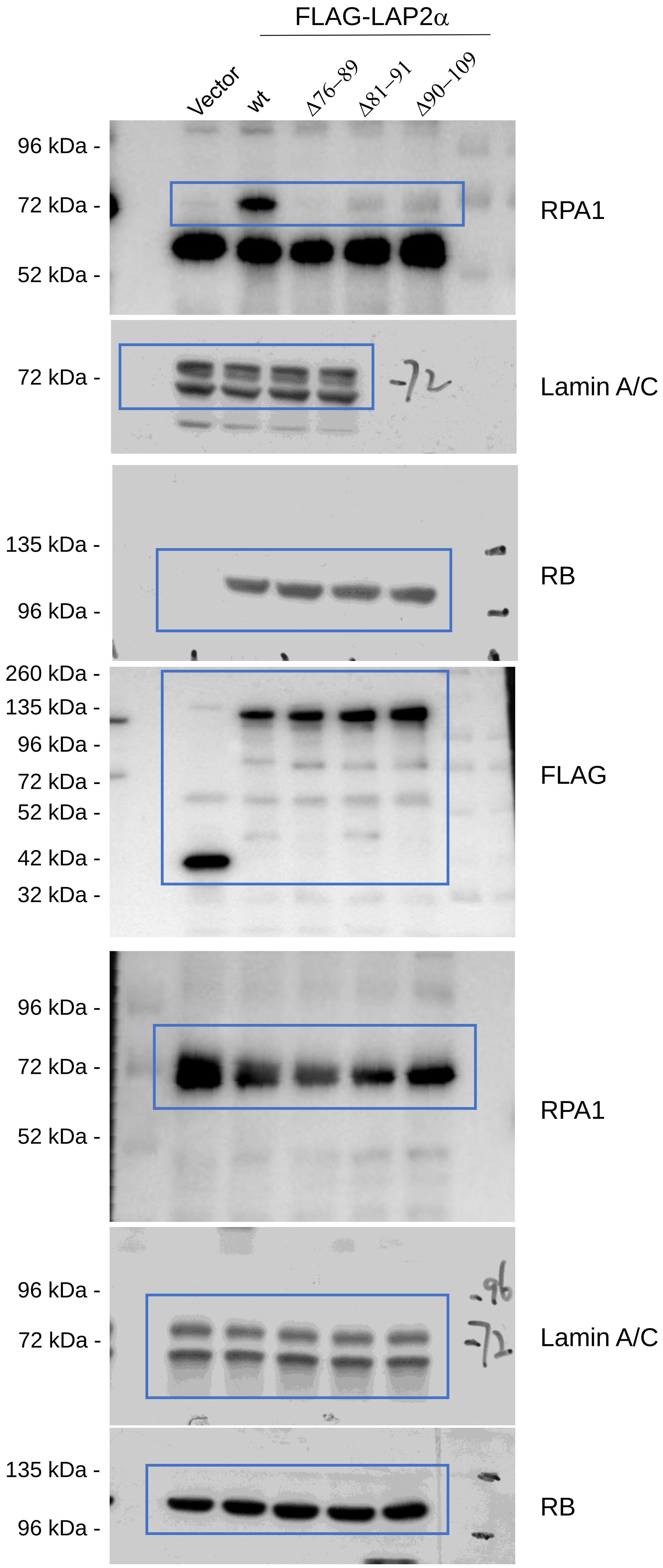

Full unedited gel for Figure 2H

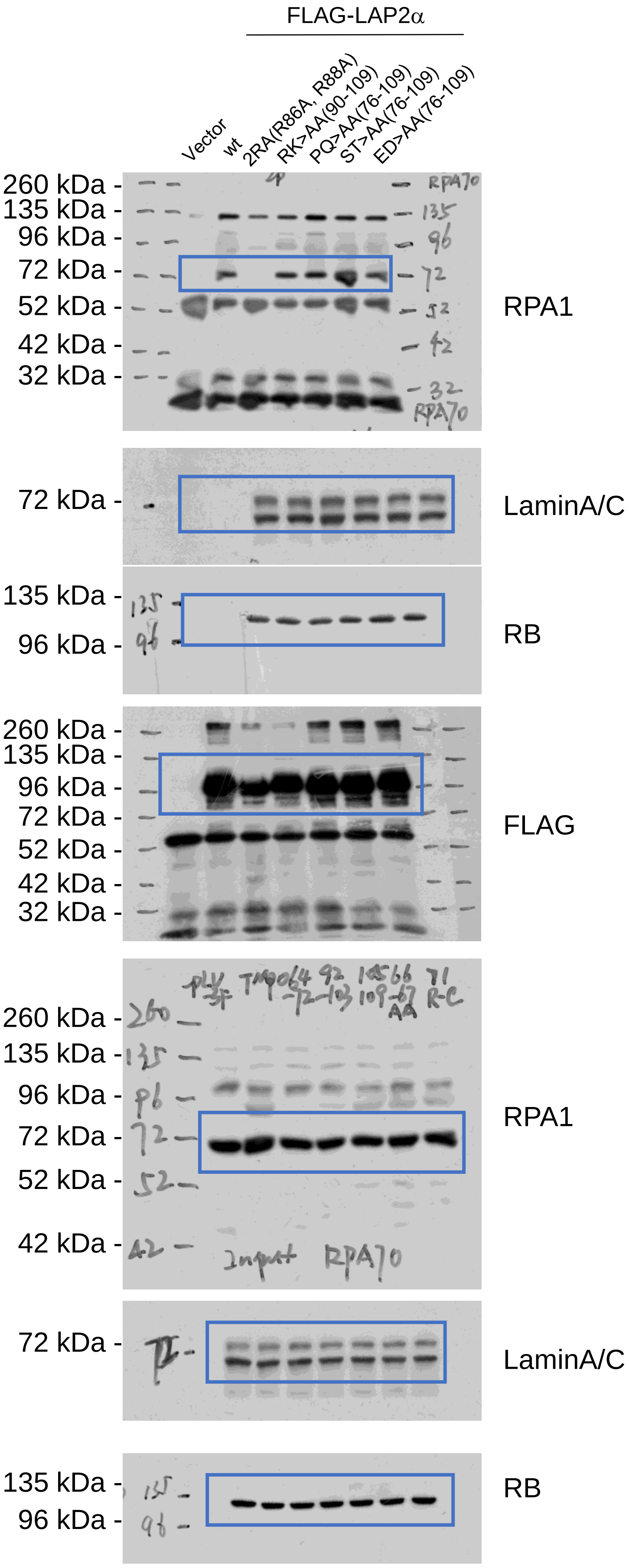

Full unedited gel for Figure 2H

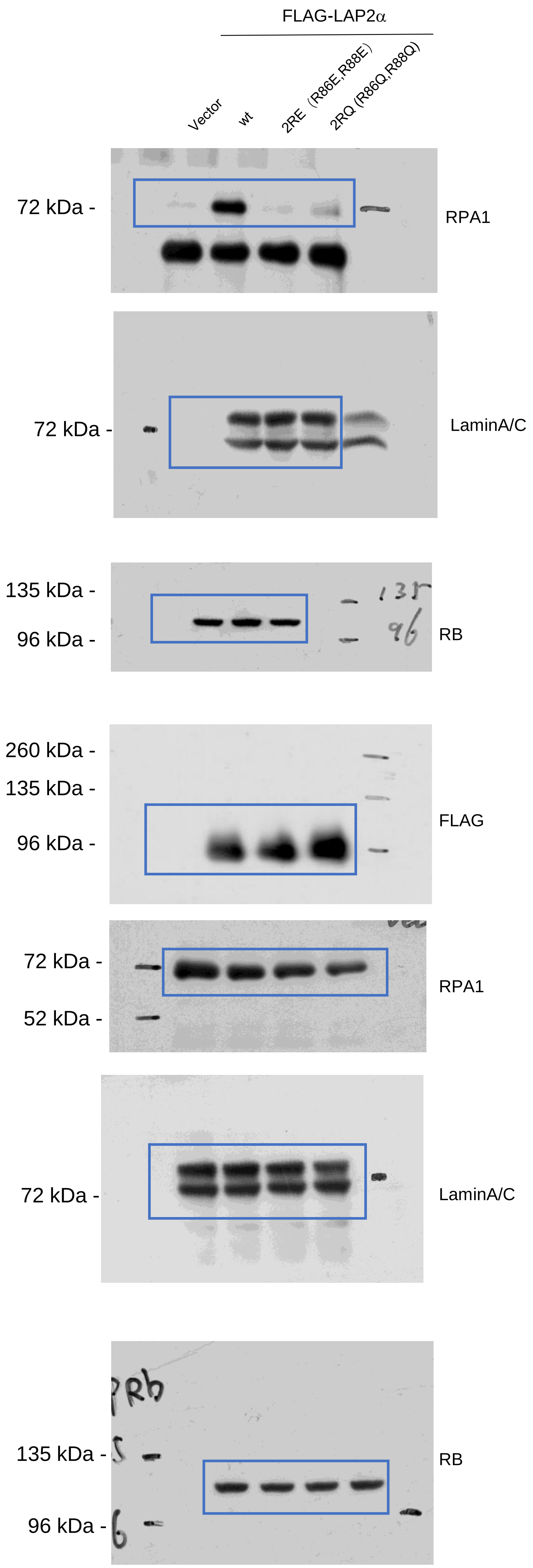

Full unedited gel for Figure 3A replicate 1

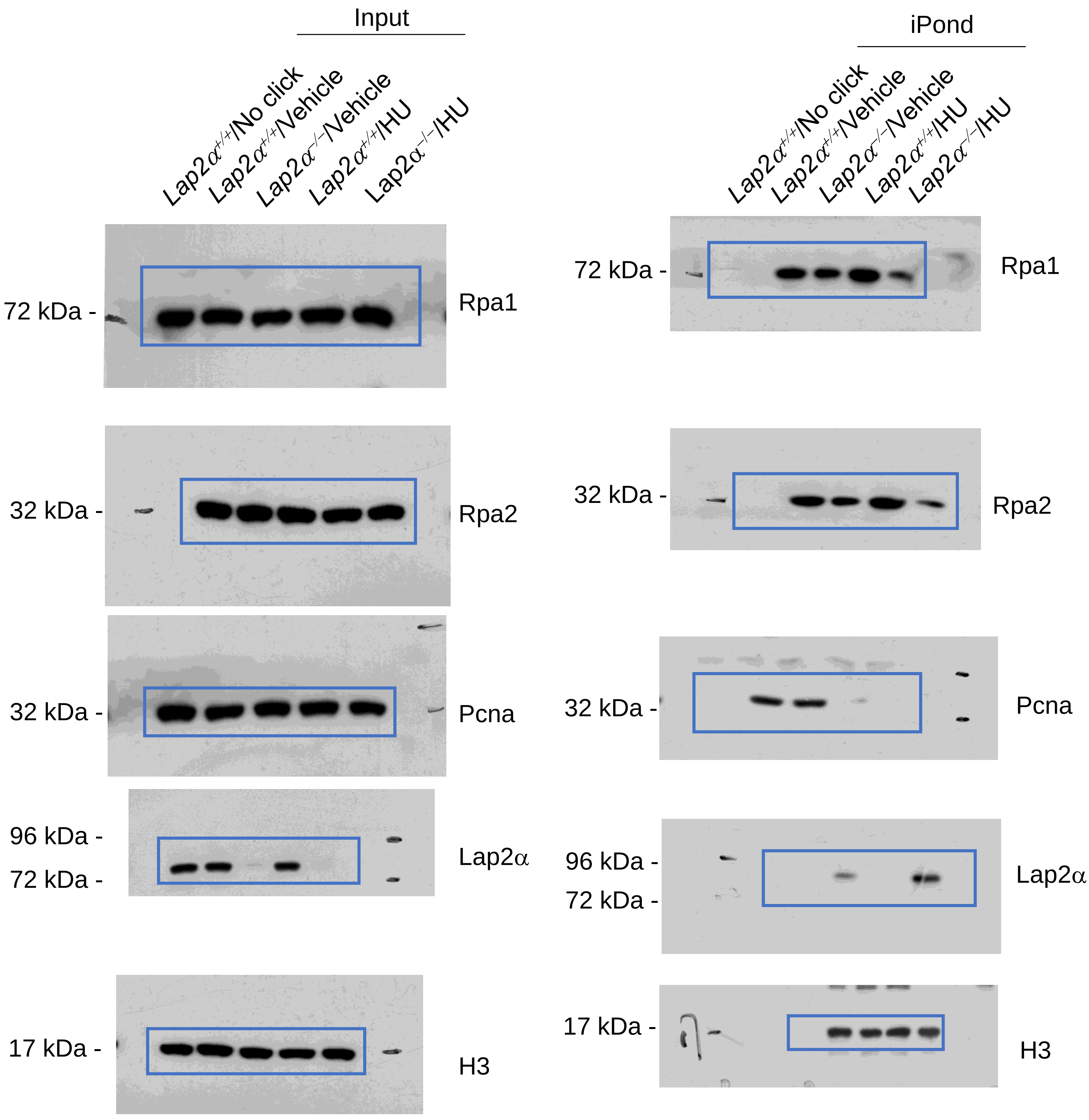

Full unedited gel for Figure 3A replicate 2

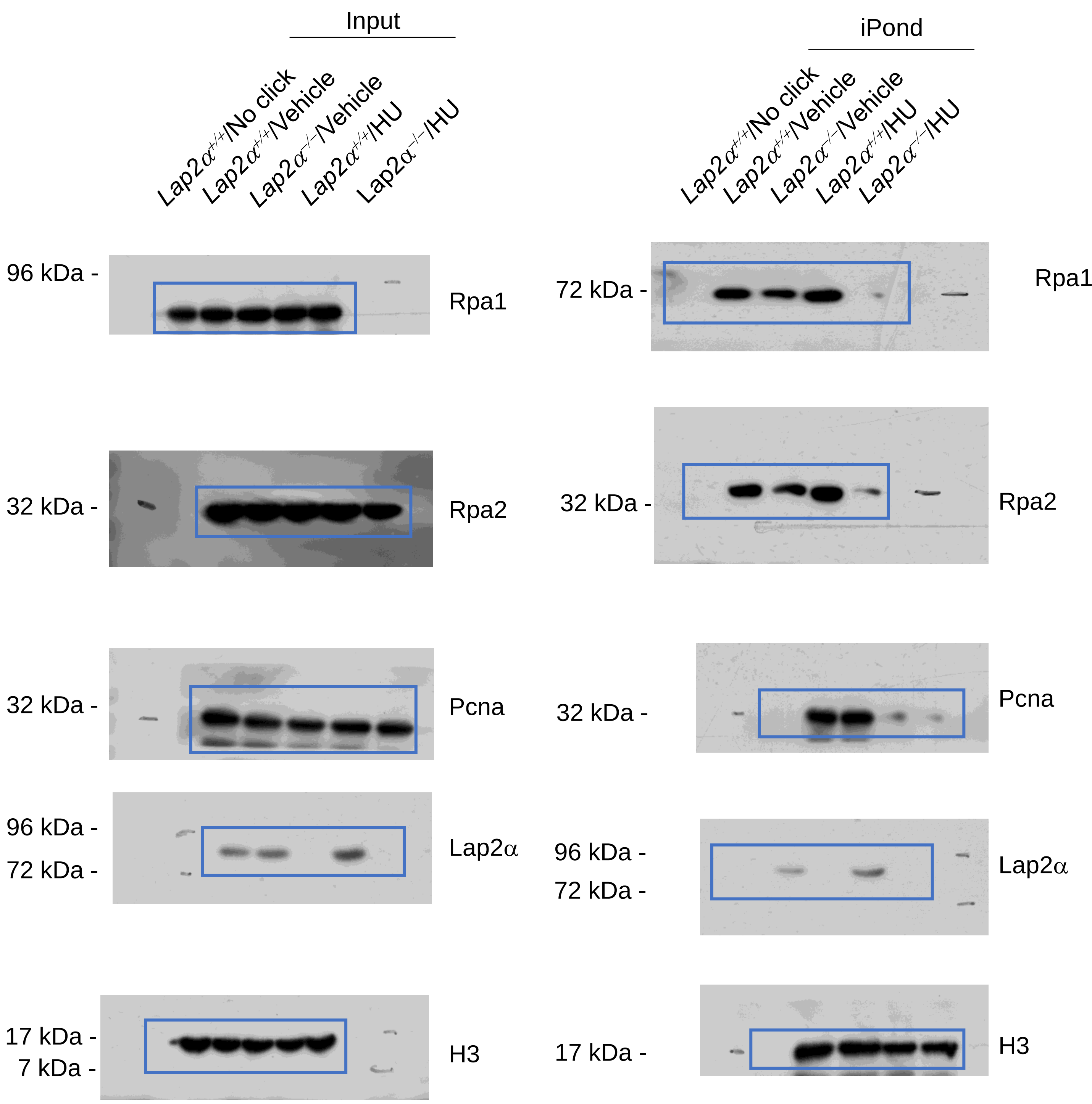

Full unedited gel for Figure 3A replicate 3

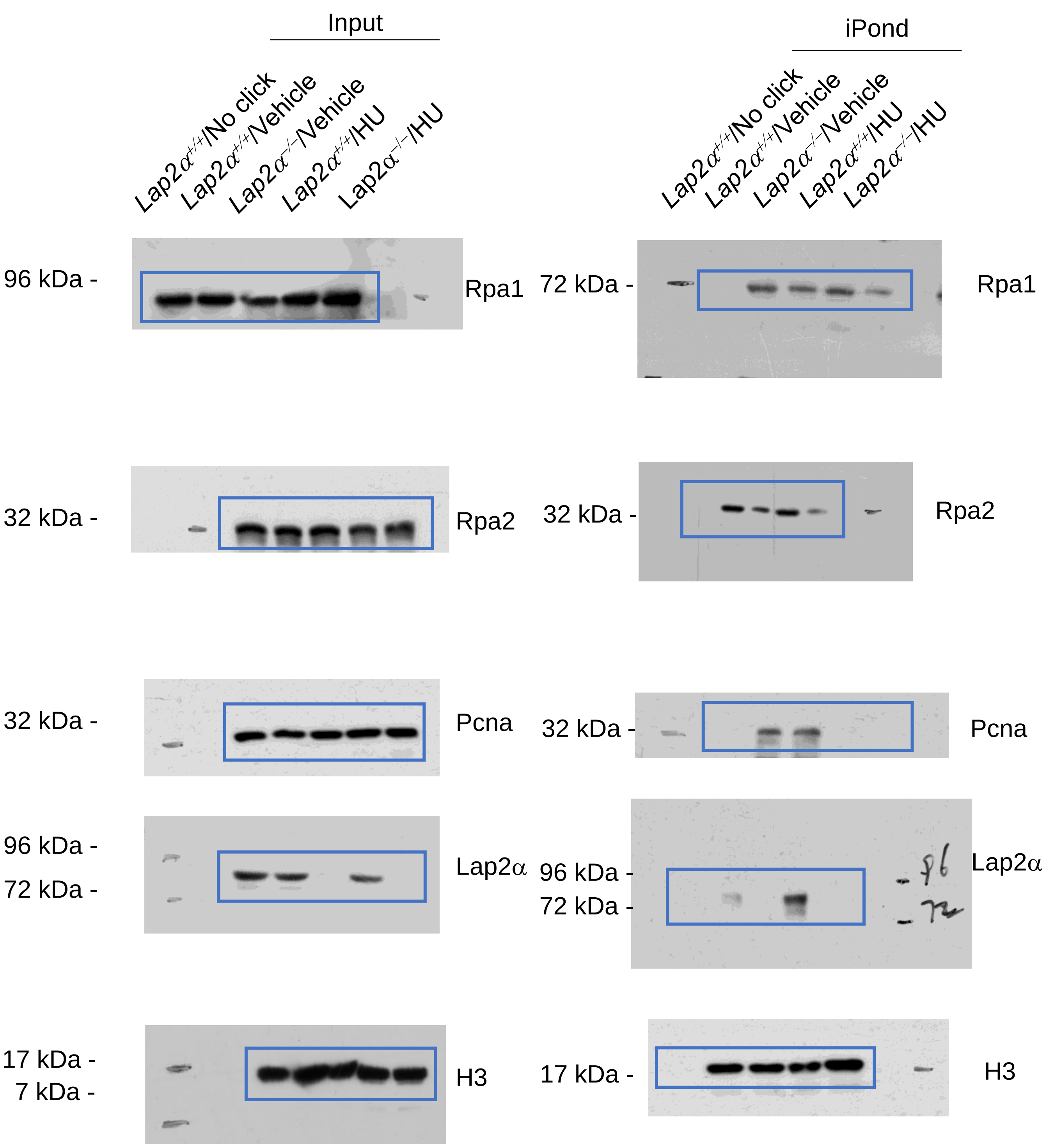

Full unedited gel for Figure 5A

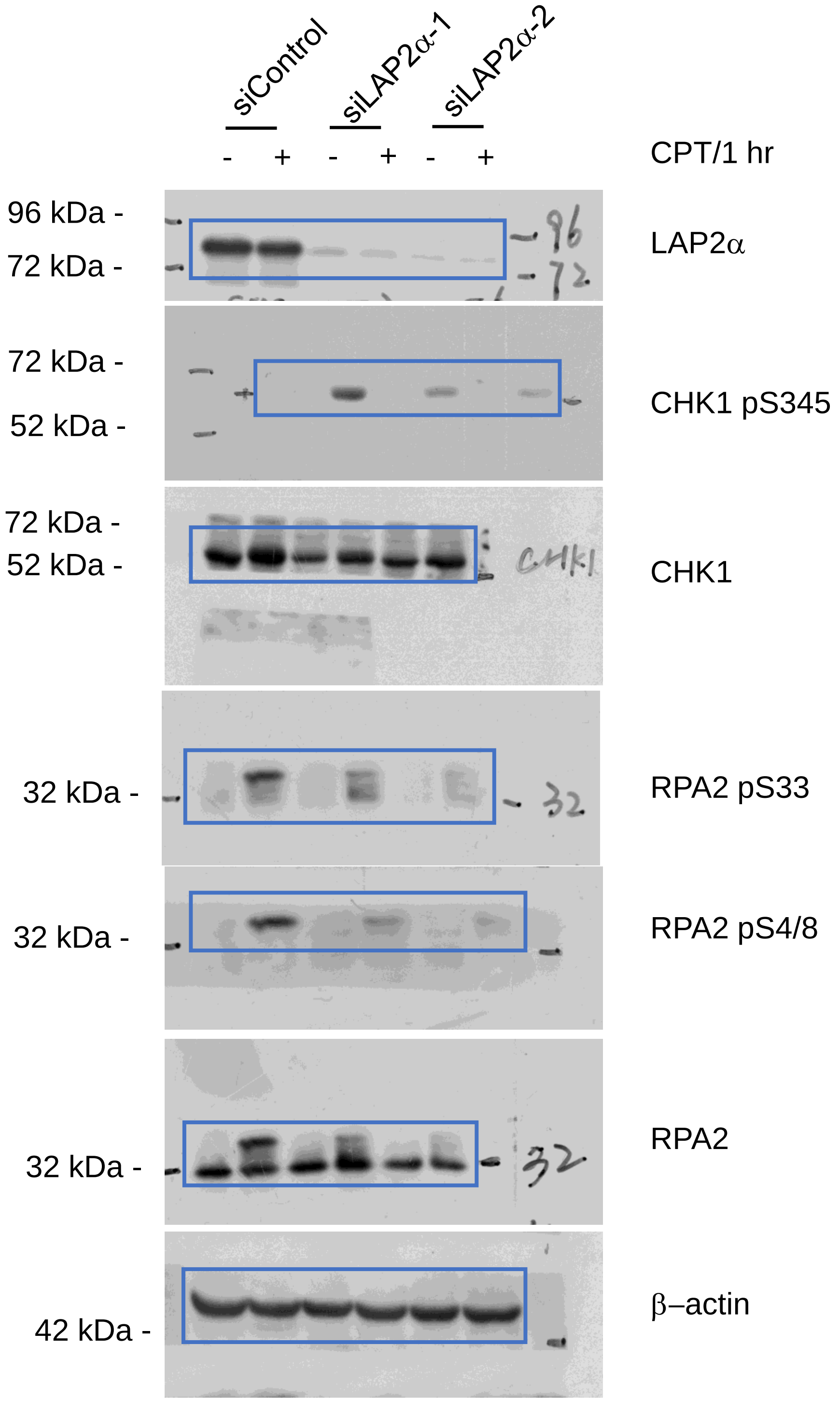

Full unedited gel for Figure 5A

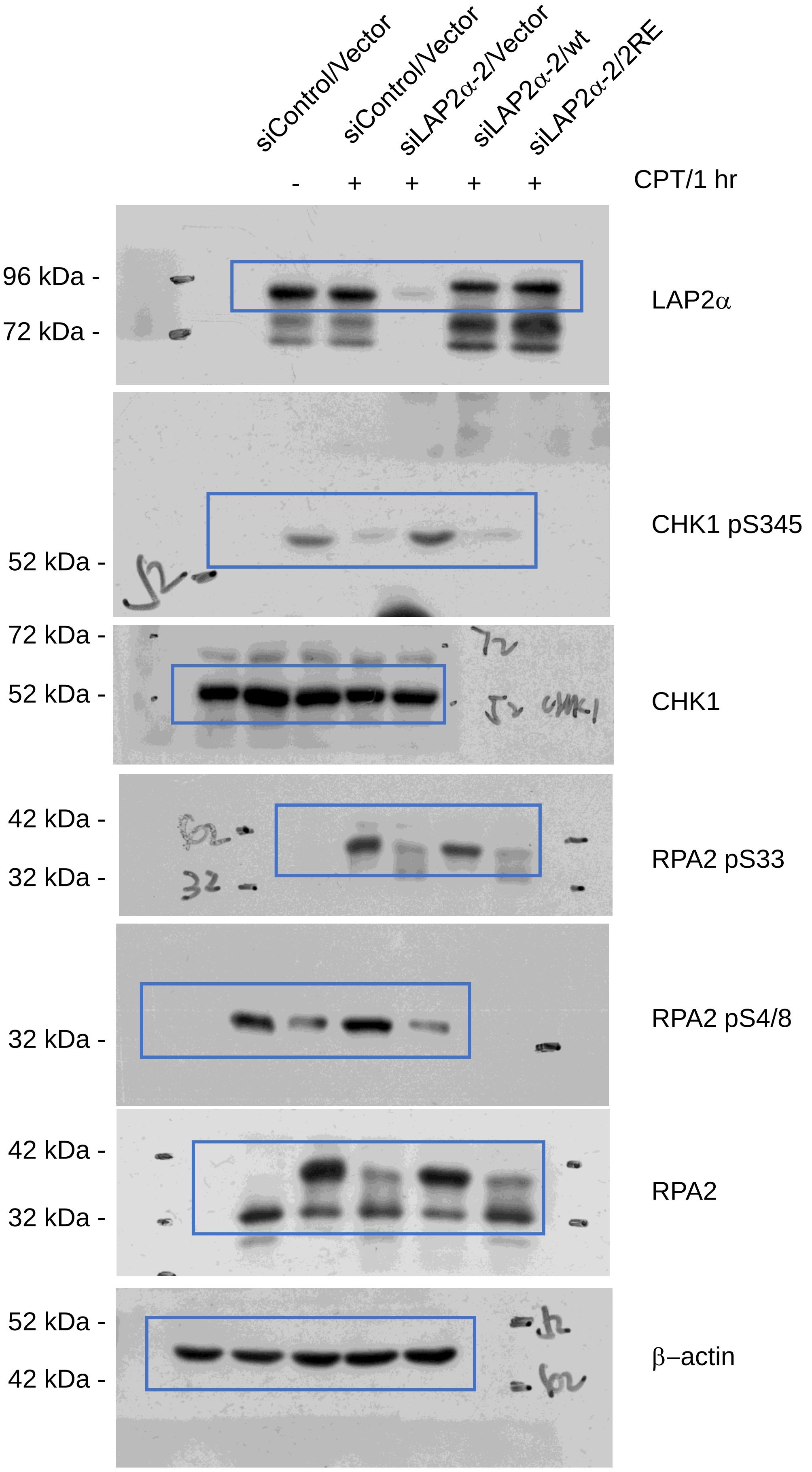

Full unedited gel for Figure 6D

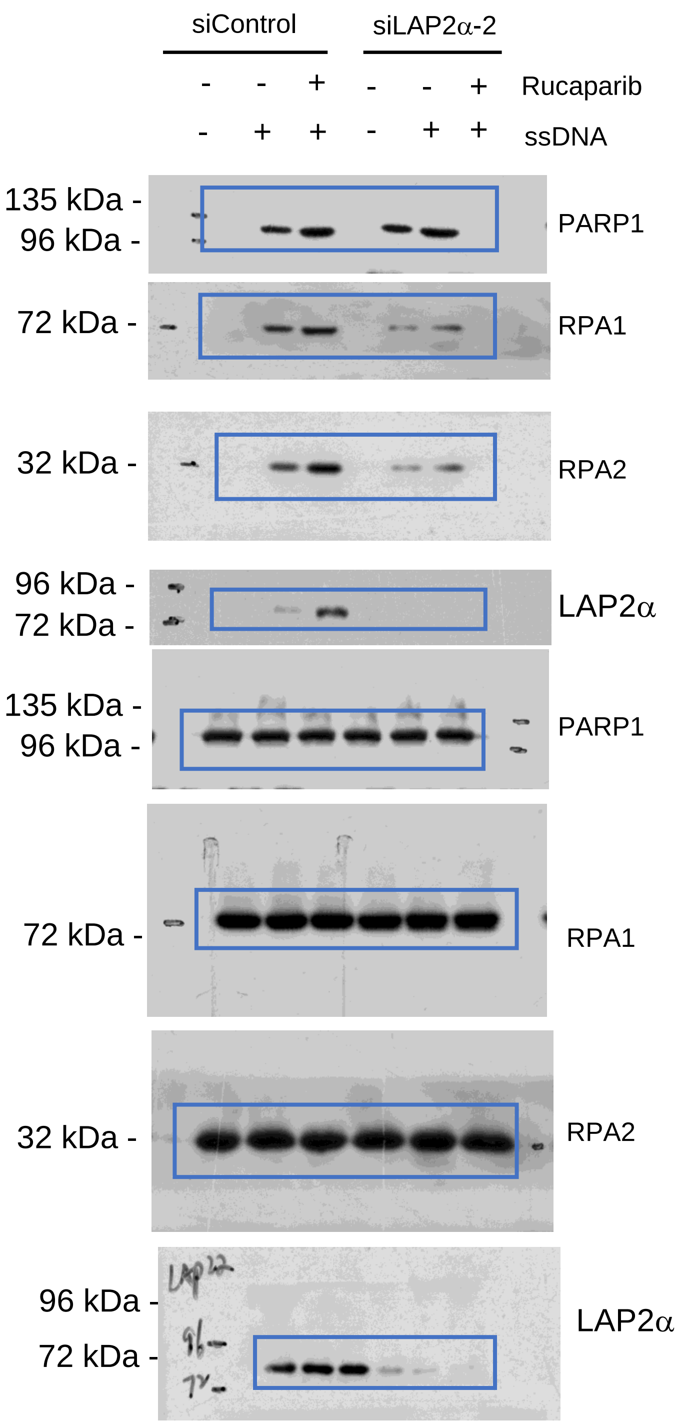

Full unedited gel for Figure 6E

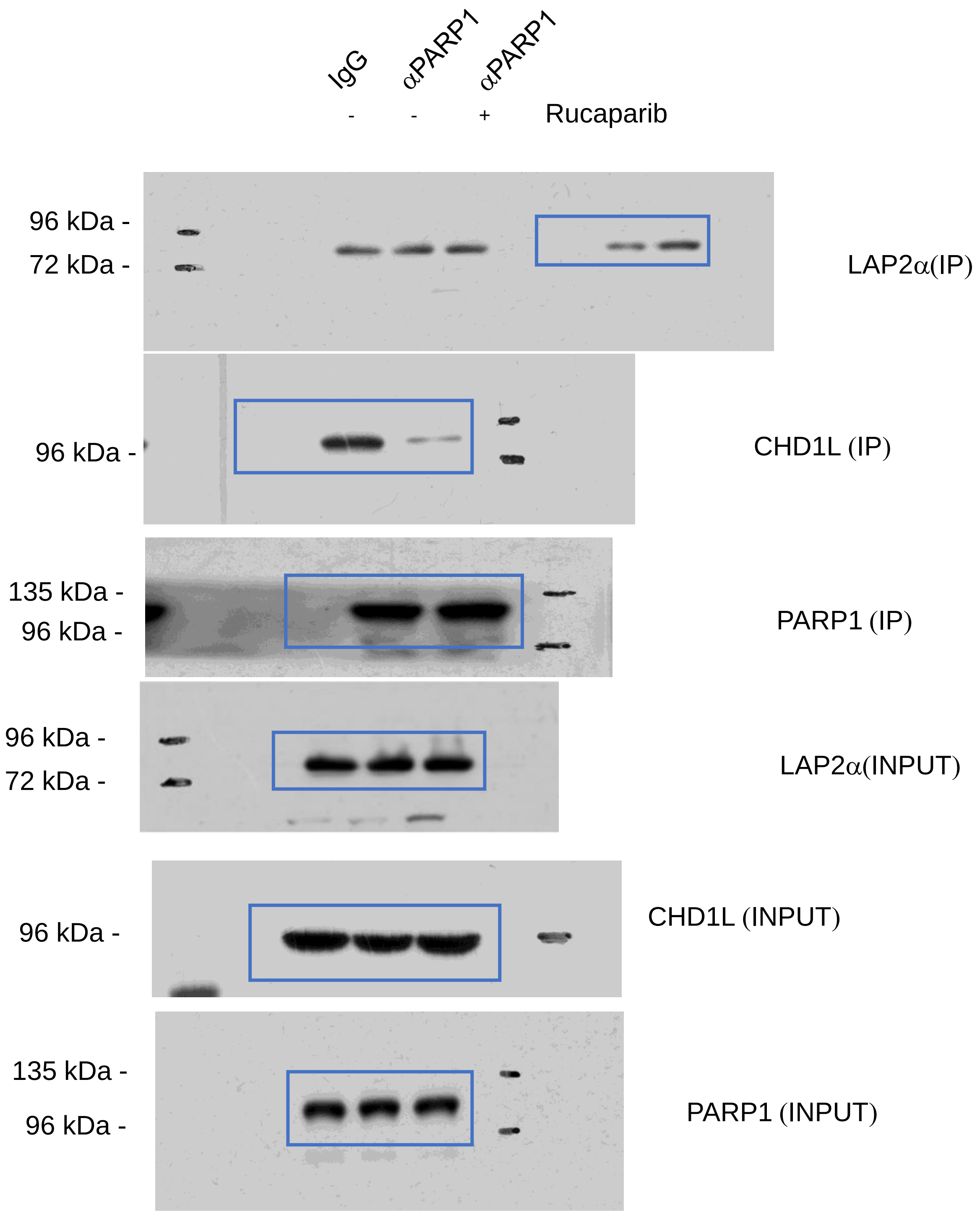

Full unedited gel for Figure S1A

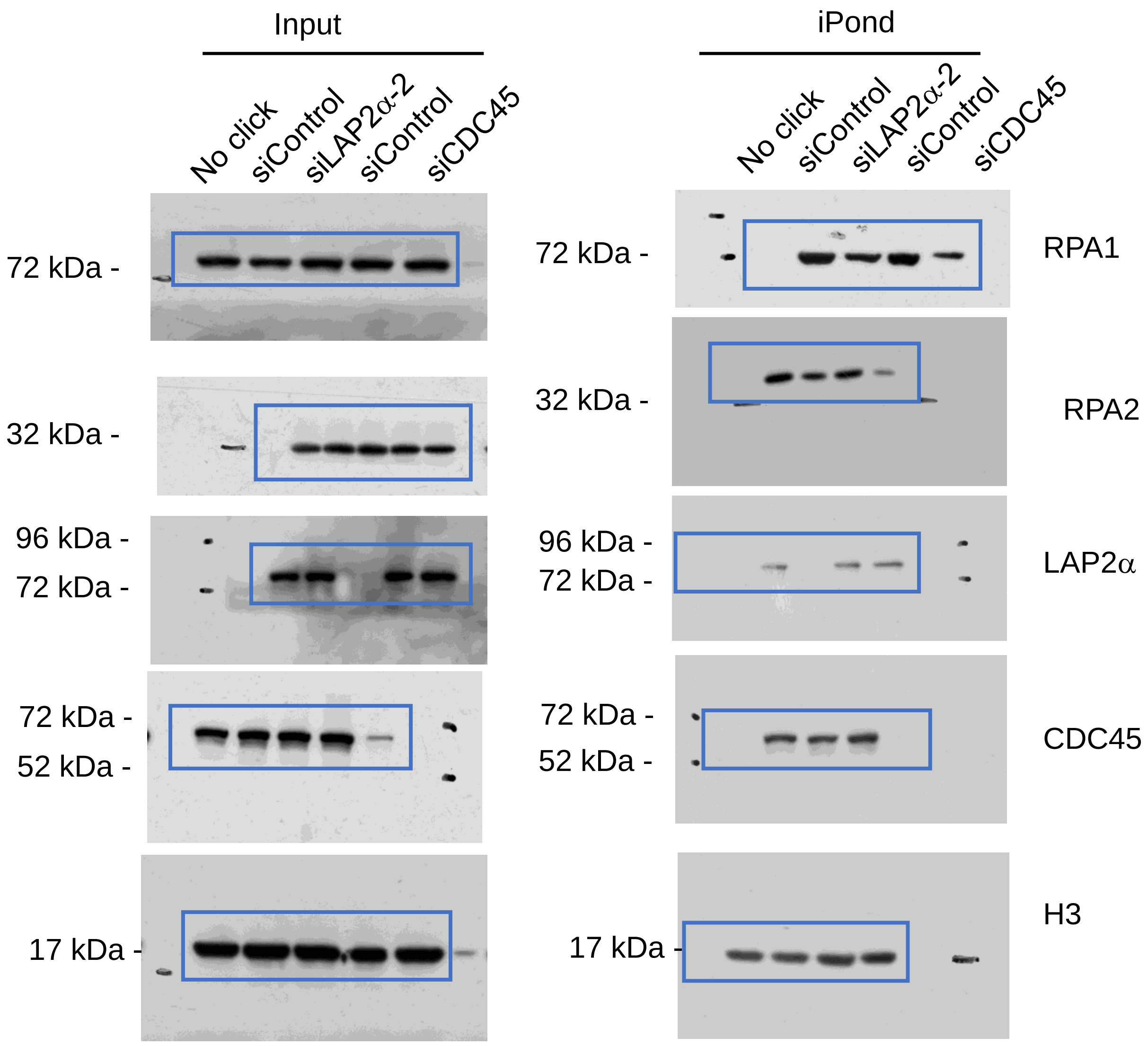

Full unedited gel for Figure S1B

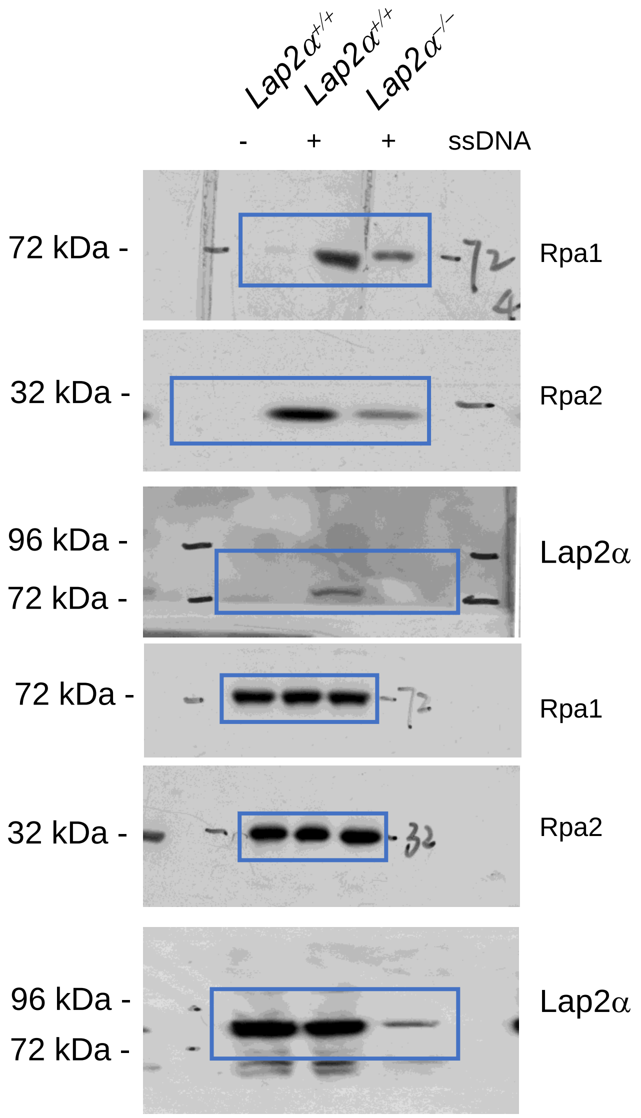

Full unedited gel for Figure S1C

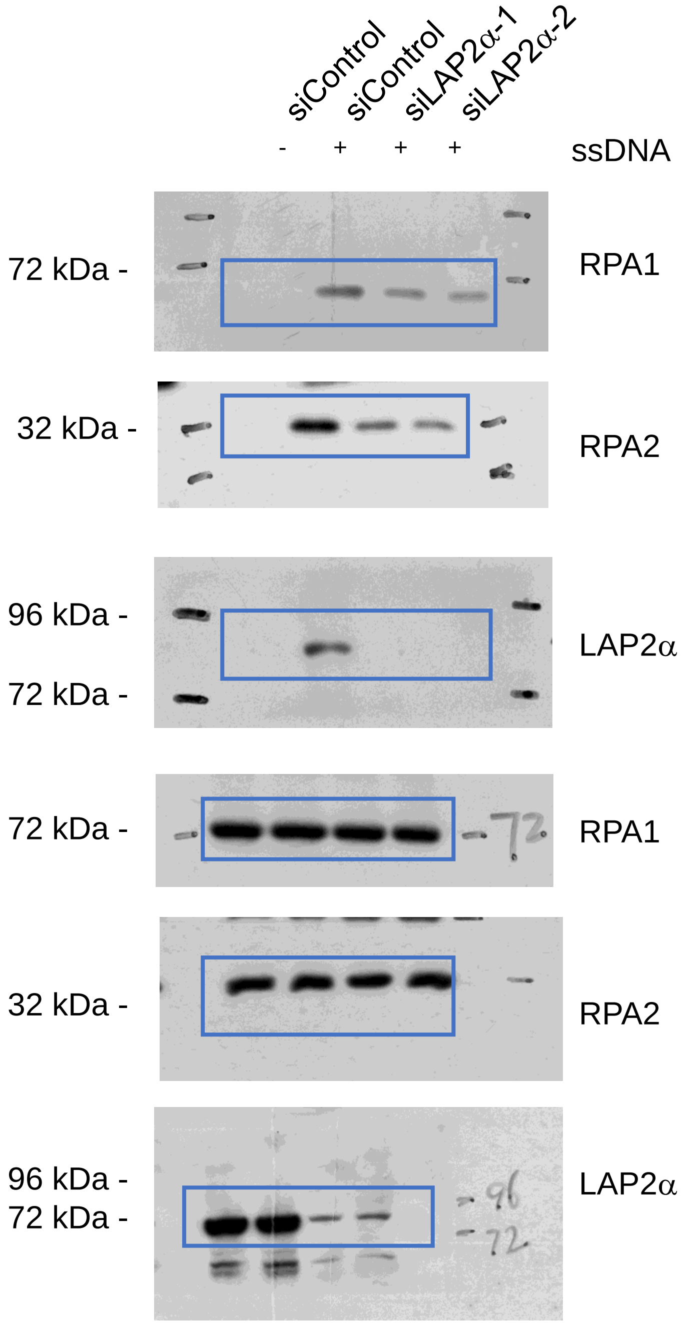

Full unedited gel for Figure S1D

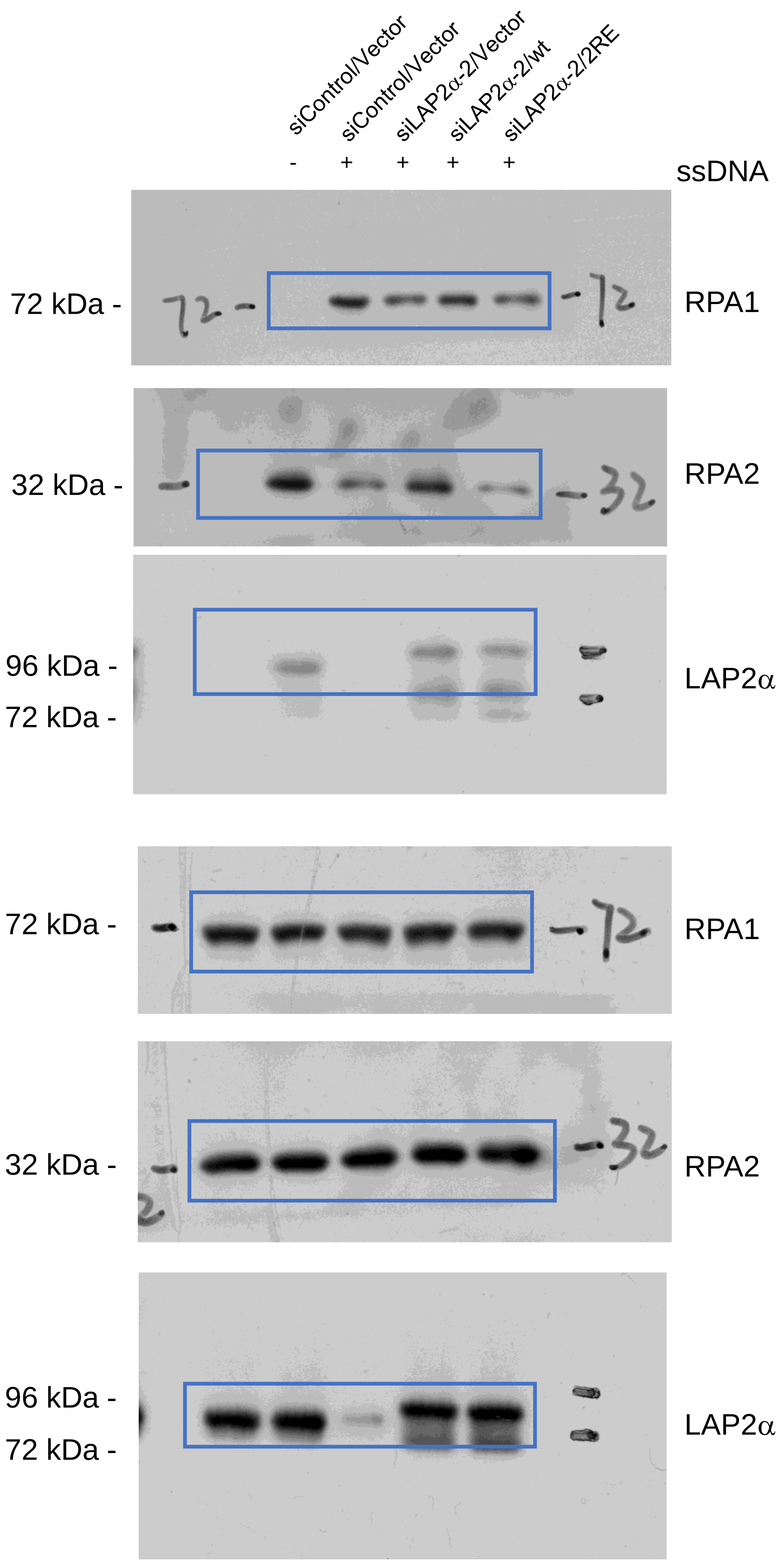

Full unedited gel for Figure S1I

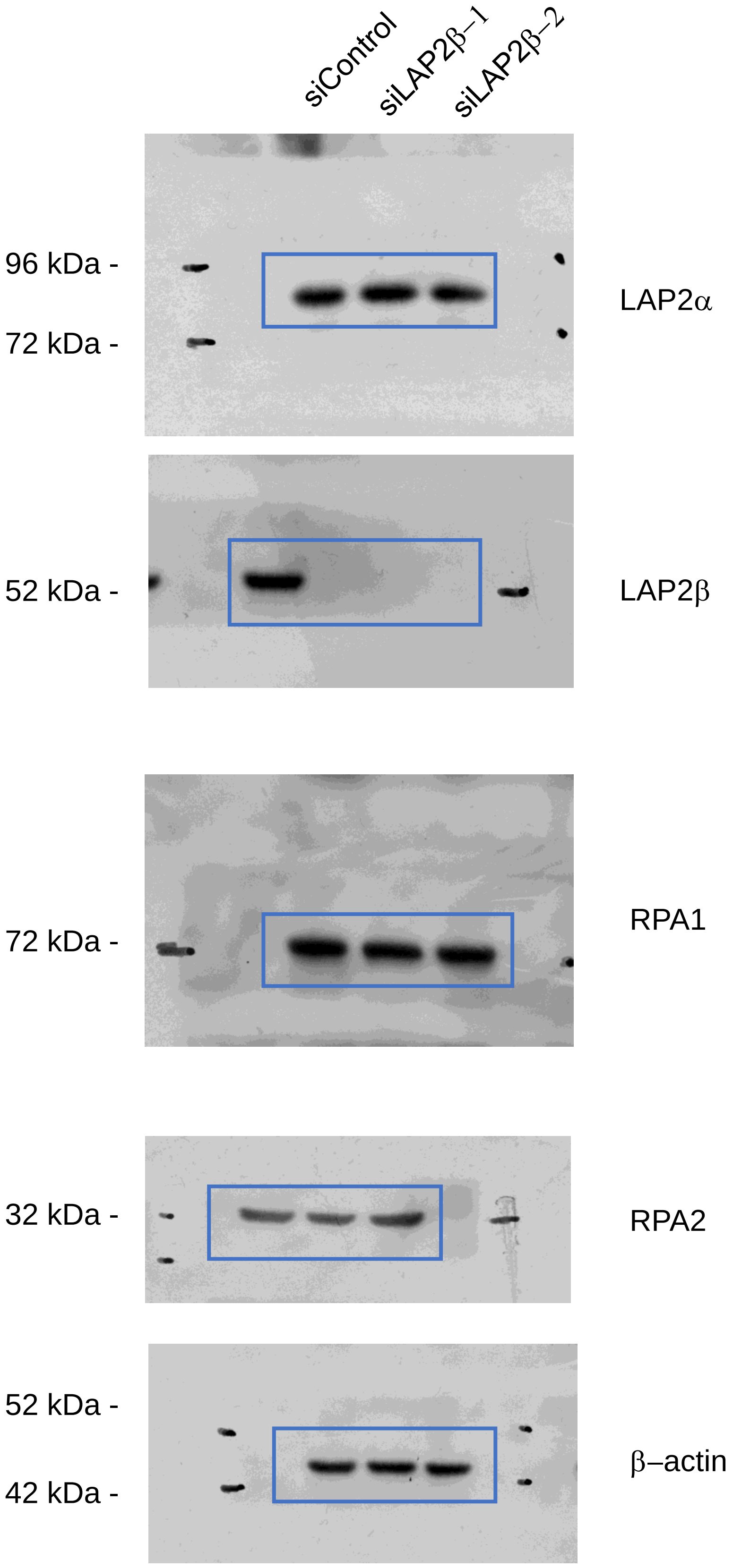

Full unedited gel for Figure S1J

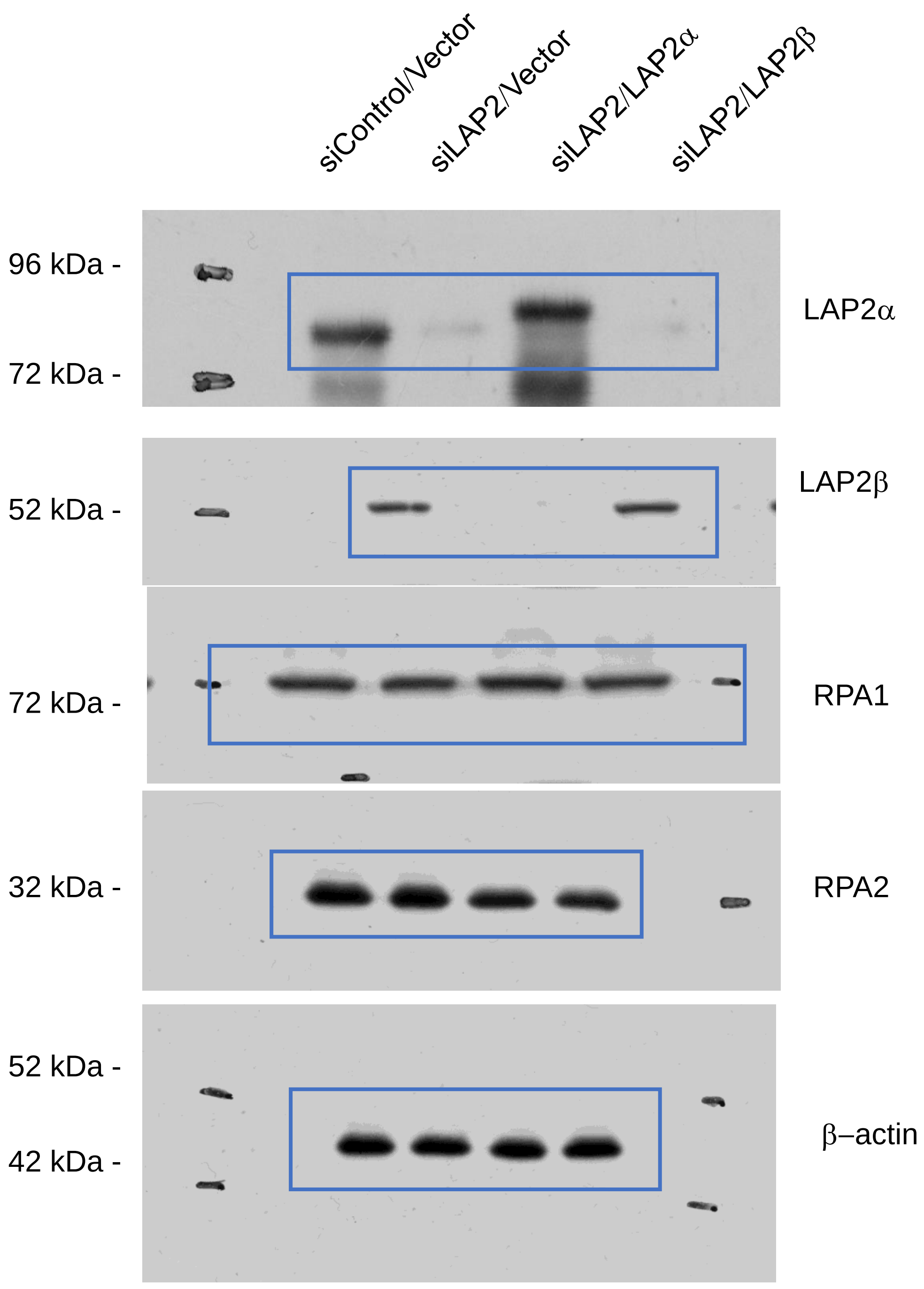

Full unedited gel for Figure S1L

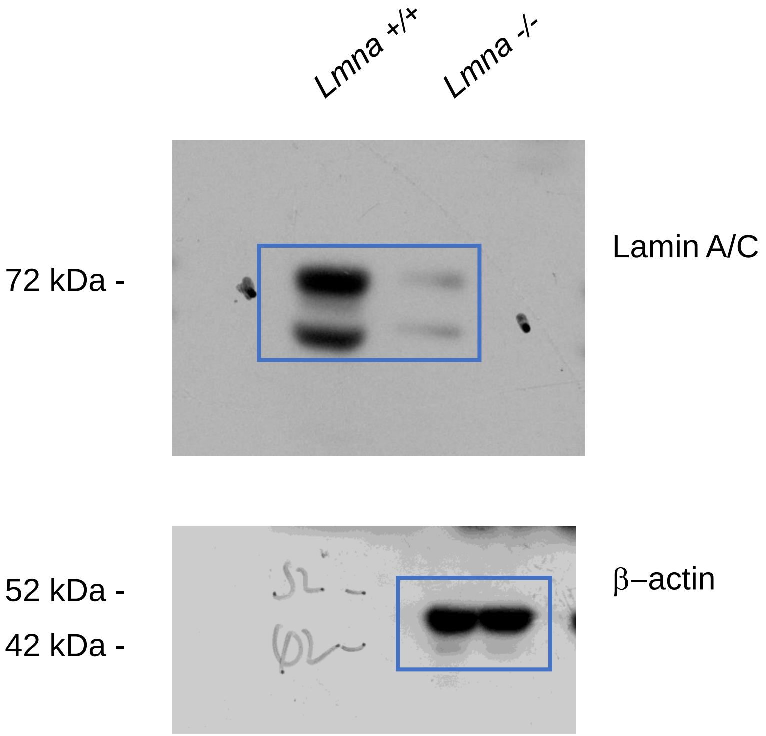

Full unedited gel for Figure S1M

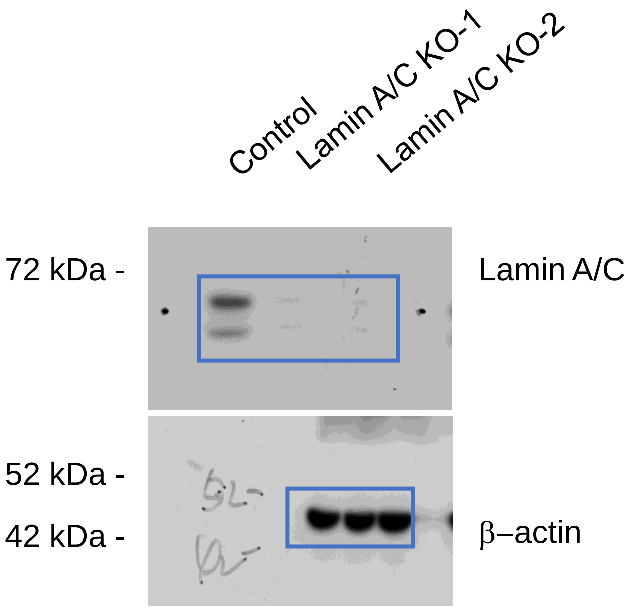

Full unedited gel for Figure S1N

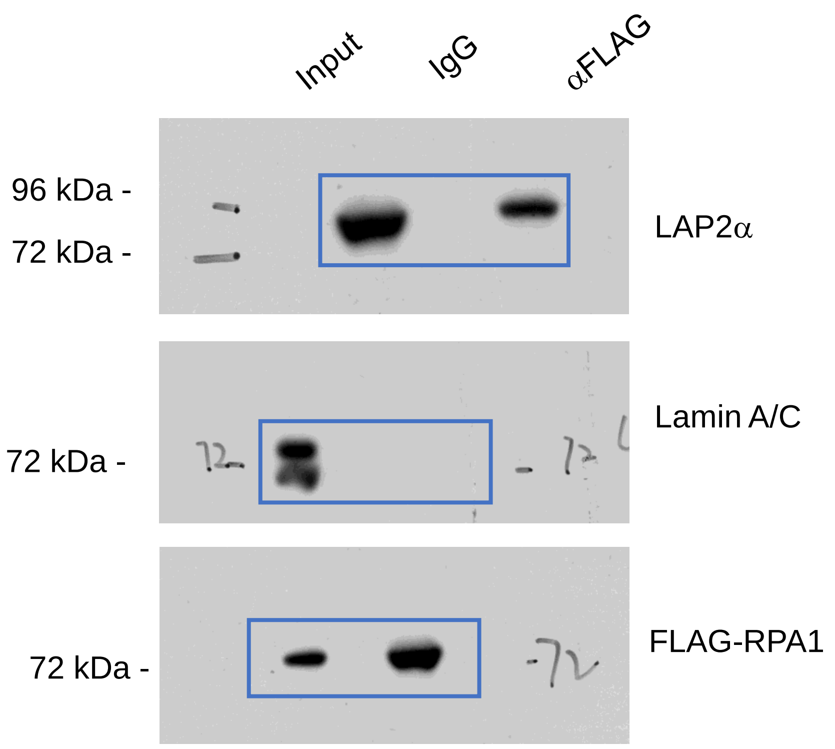

Full unedited gel for Figure S2B

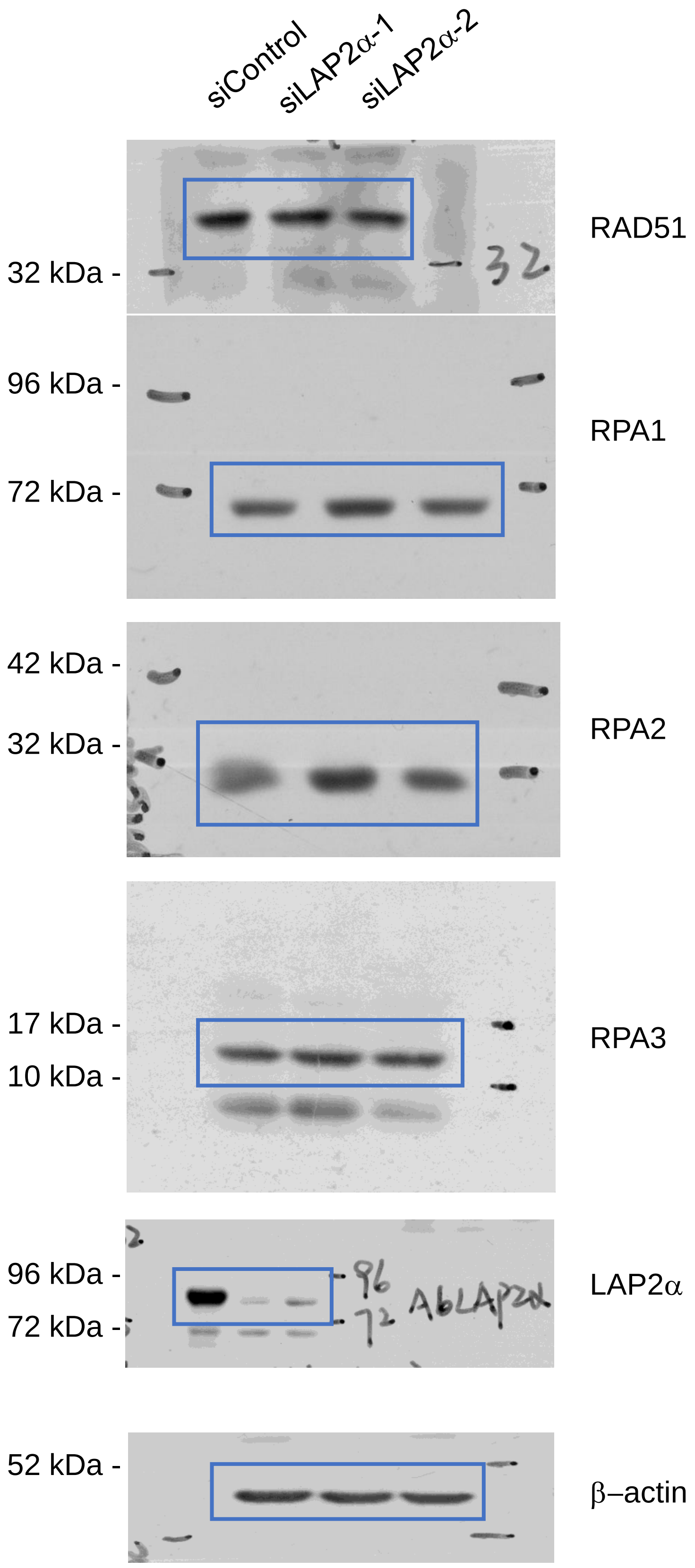

Full unedited gel for Figure S2E

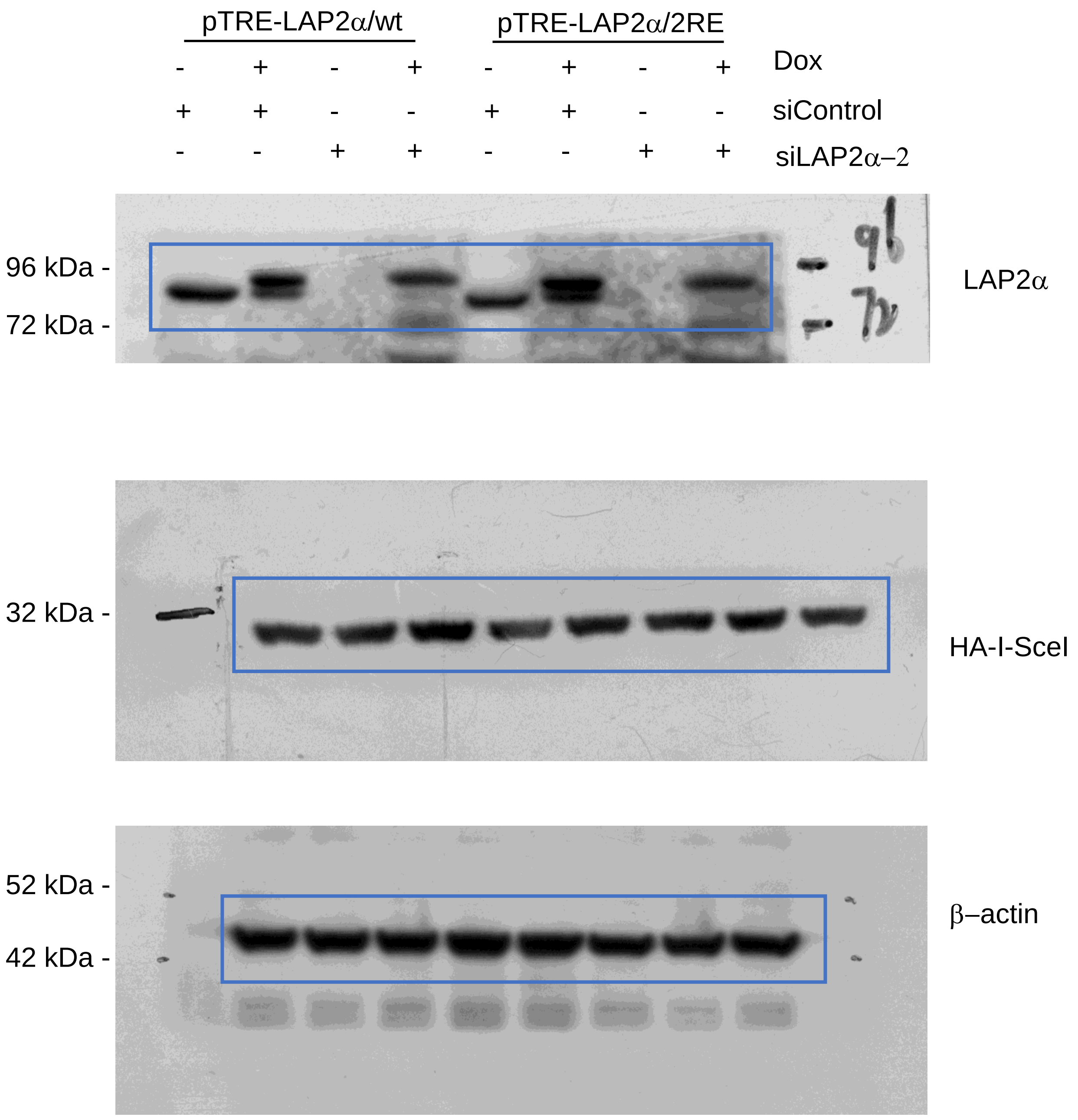

Full unedited gel for Figure S3C

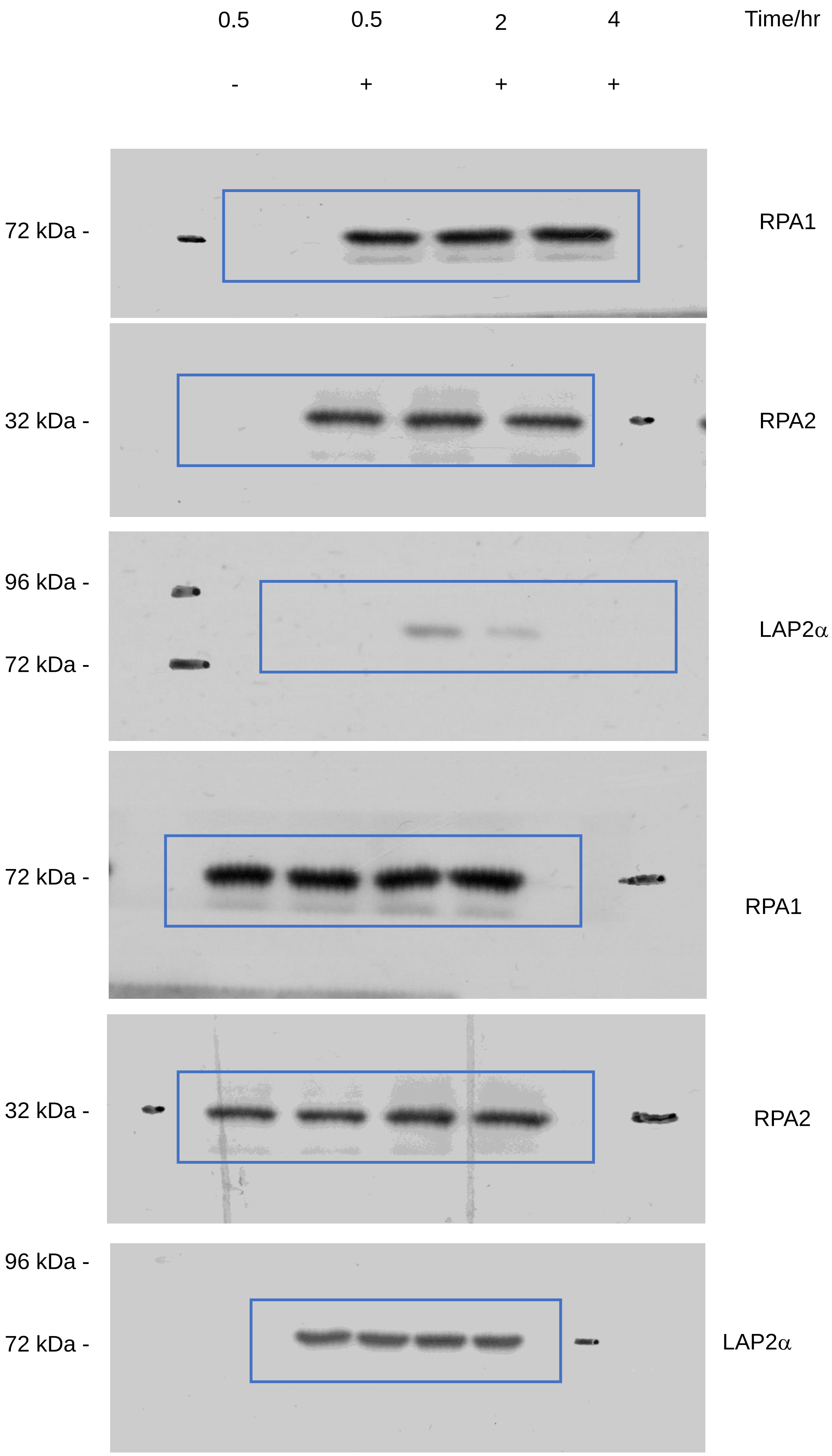

Full unedited gel for Figure S3F

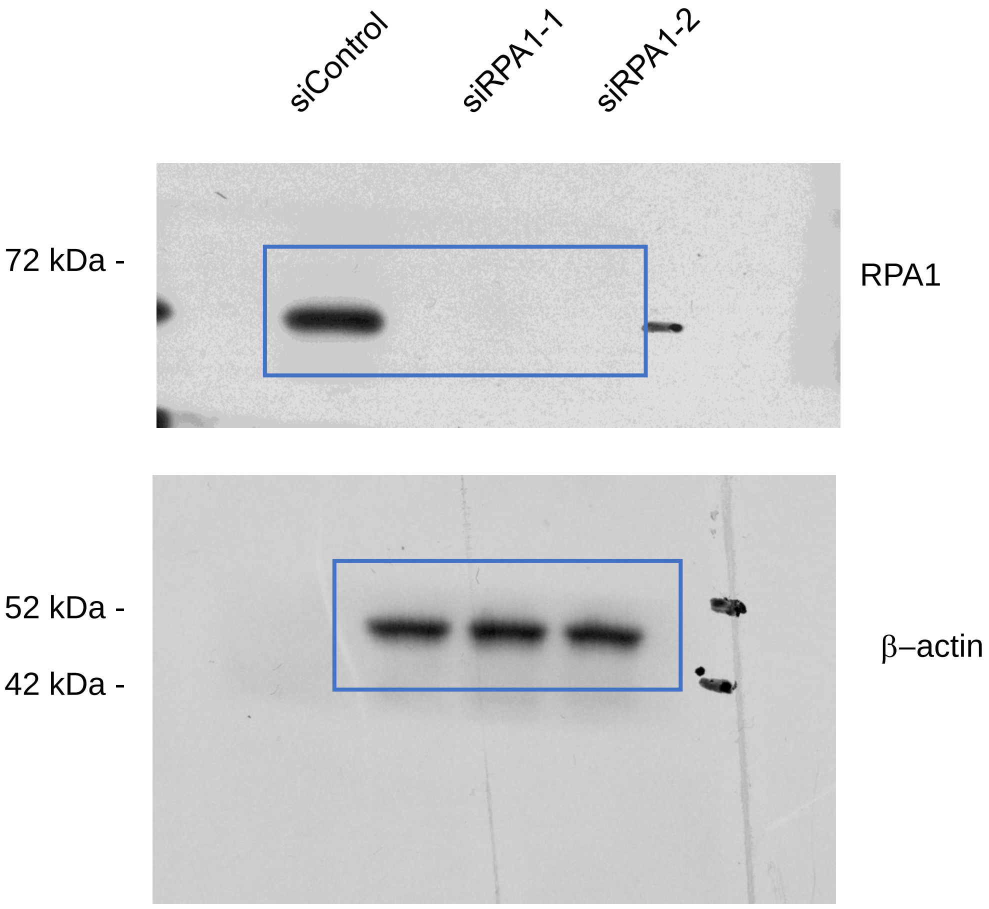

Full unedited gel for Figure S3I

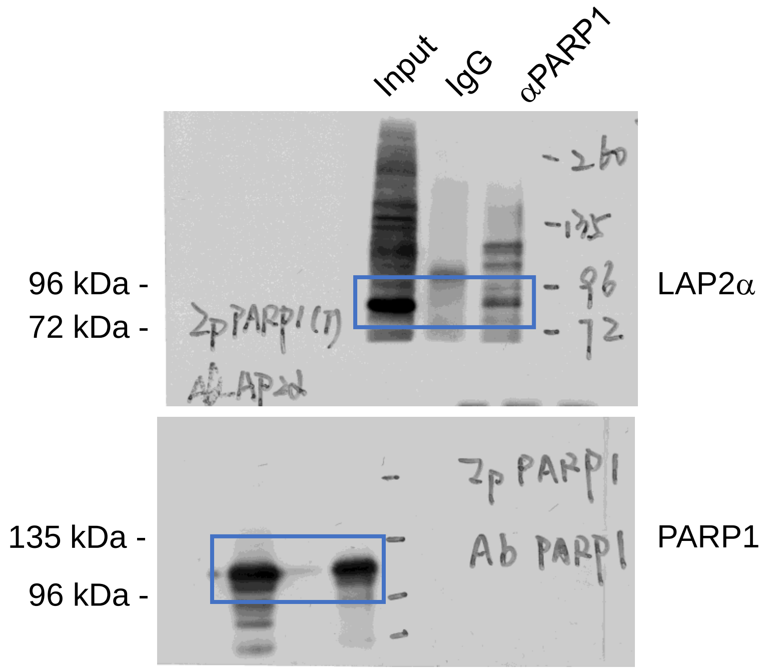

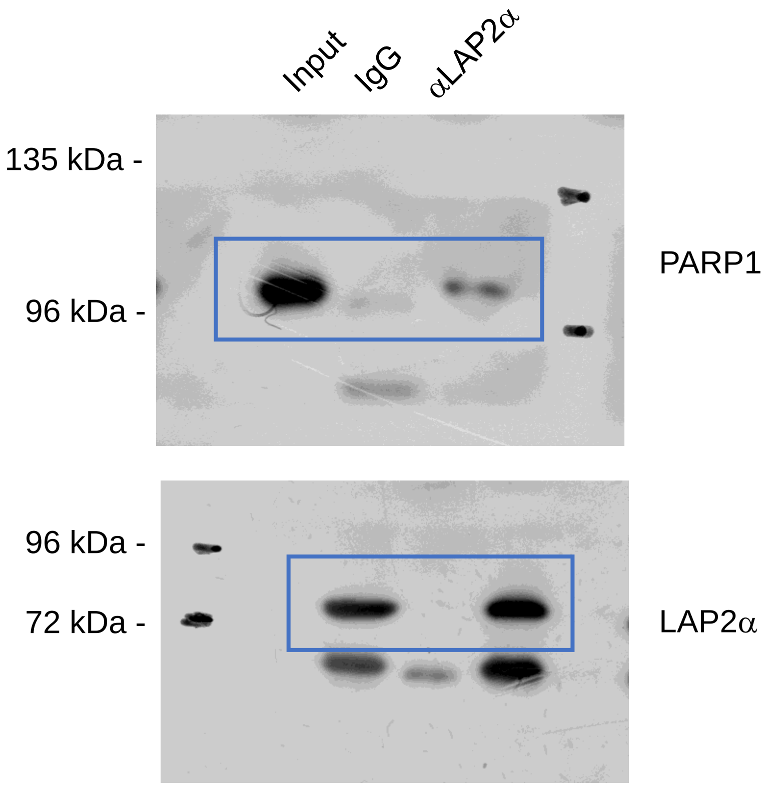

Full unedited gel for Figure S3J

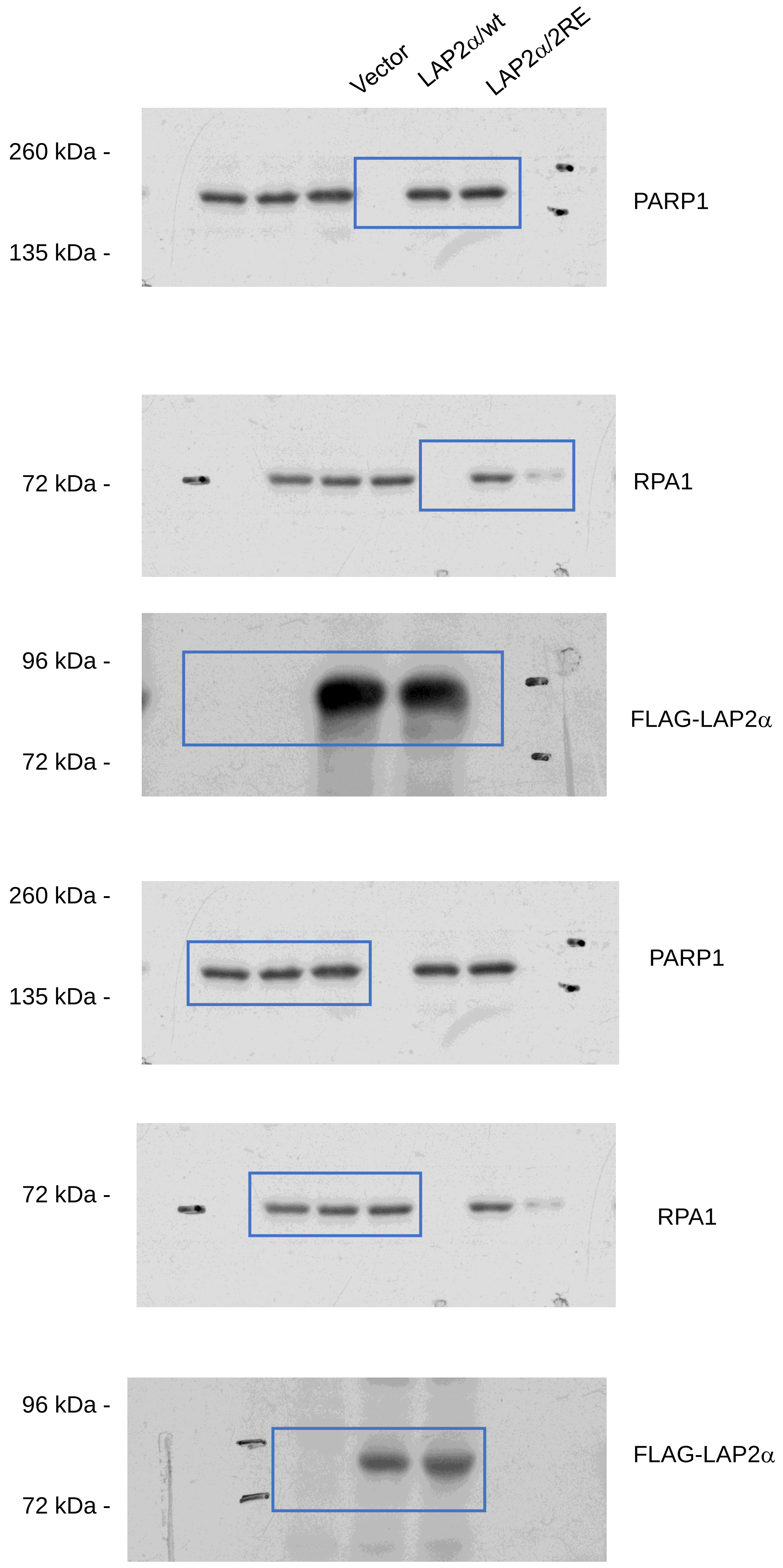

Full unedited gel for Figure S3K

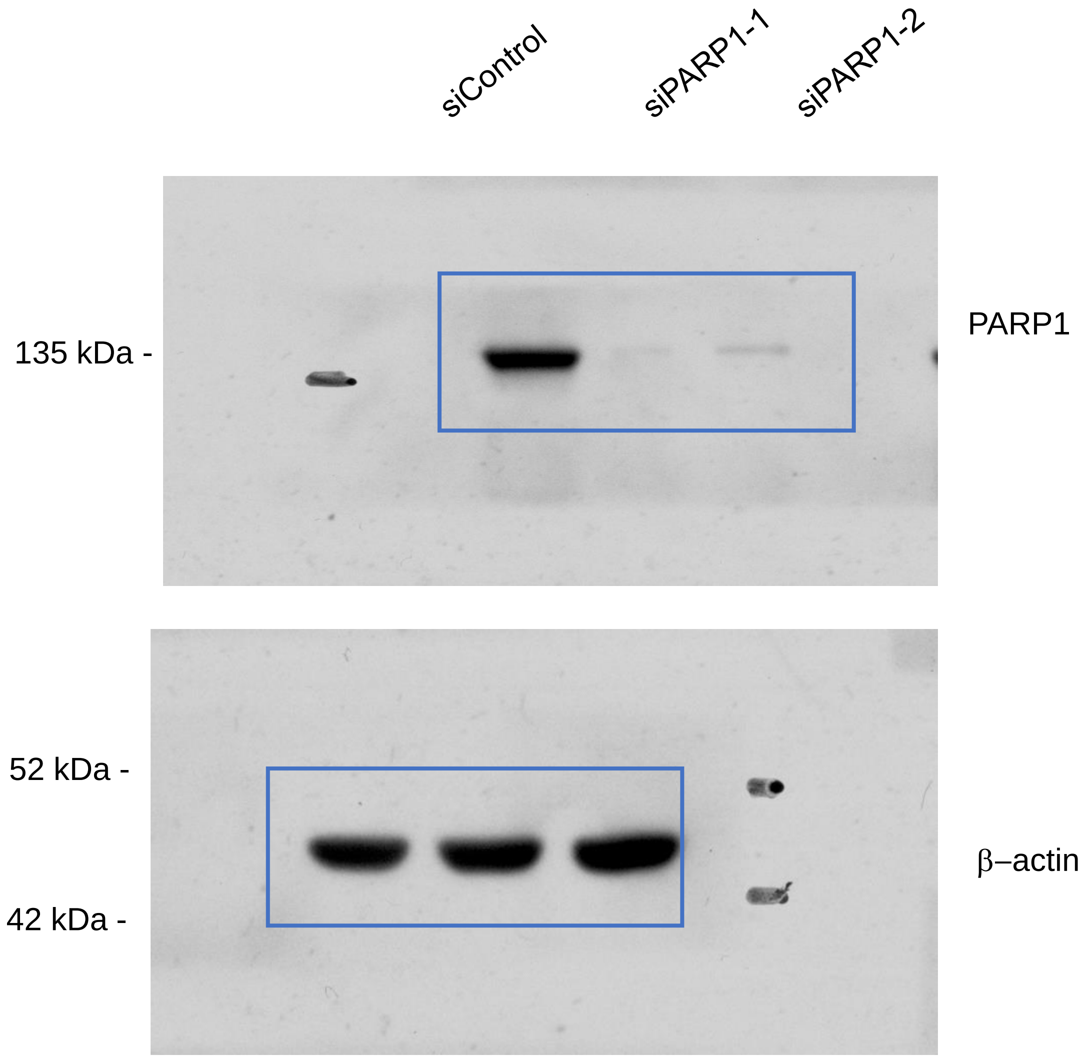

Full unedited gel for Figure S3M

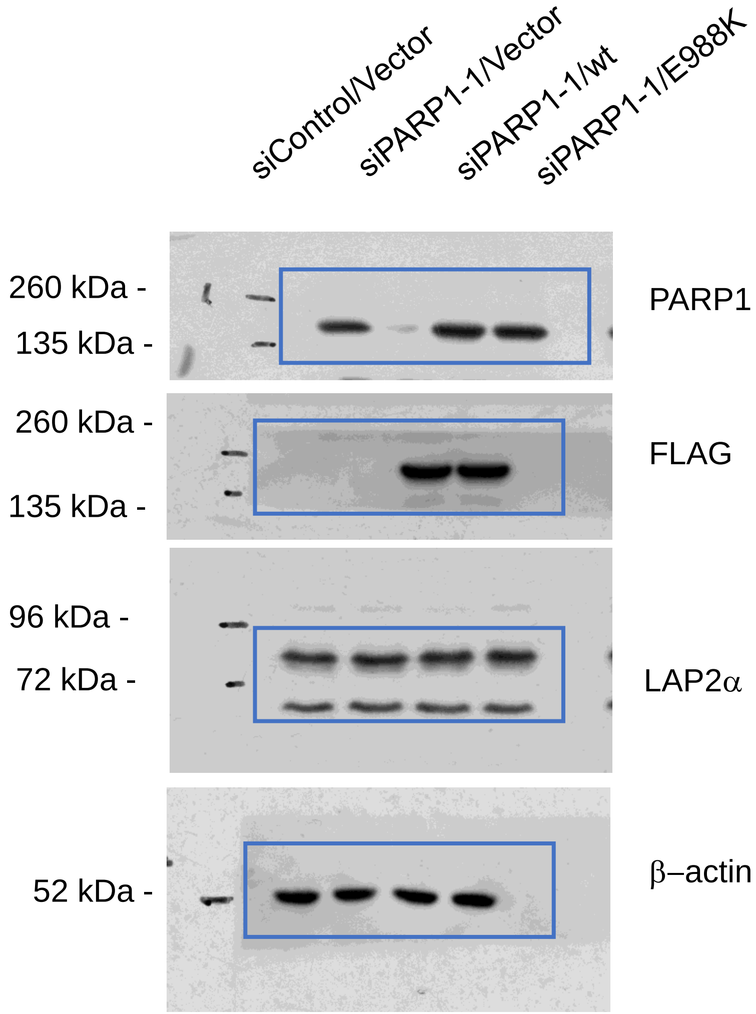

Full unedited gel for Figure S3N

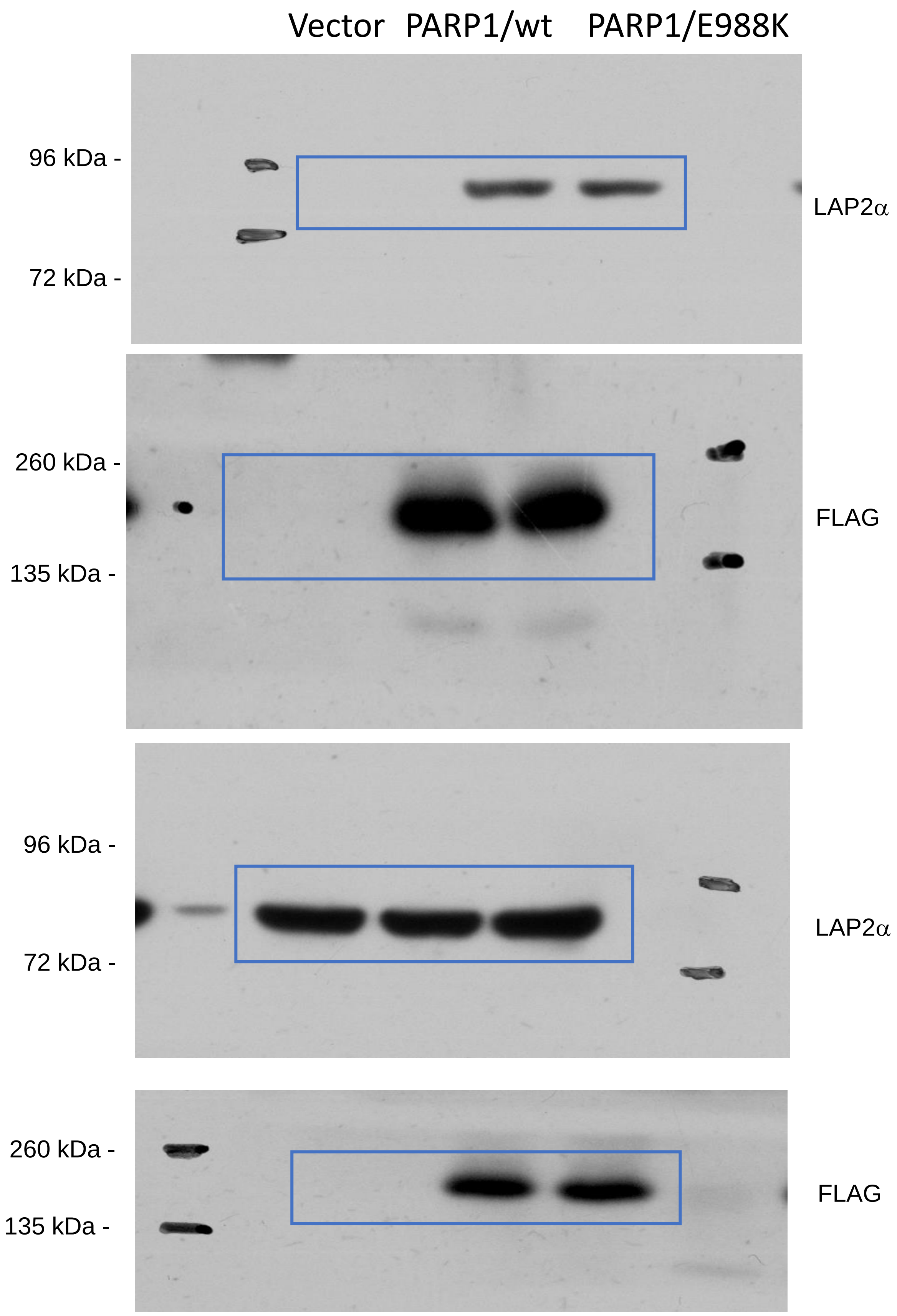

Supplement: Supplementary file 5 — Additional file 5. Uncropped versions of all blots. [file 13059_2022_2638_MOESM5_ESM.pdf]
