# Supplementary material for: LAP2α preserves genome integrity through assisting RPA deposition on damaged chromatin
Source: Genome Biol. 2022 Feb 28;23:64. doi: 10.1186/s13059-022-02638-6 (PMC8883701; doi:10.1186/s13059-022-02638-6)
Supplement: Supplementary file 6 — Additional file 6. Review history. [file 13059_2022_2638_MOESM6_ESM.docx]

**1^st^ round**

**Reviewer 1**

In this manuscript Bao et al describe a novel role for Lap2alpha in loading RPA to ssDNA. Lap2alpha is convincingly shown to contribute to RPA-mediated signaling upon replication stress and DNA damage. Furthermore, the authors show that signaling is mediated through ATR and loading of RPA involves PARP1. This work represents a heroic biochemical effort that convincingly links Lap2alpha to DNA repair mechanisms via RPA. A role for Lap2alpha in DNA repair has been suggested previously, but to my knowledge, has not been shown so convincingly through a direct link as presented in this manuscript. I do wonder though; how suitable this work is for the audience of Genome Biology. This manuscript does not include any genomics.

General comments

1. I qualified this work as a heroic effort, yet this also makes it difficult to digest for some parts of the text/figures. Some more specific comments will follow below, yet in general the manuscript could benefit from more extensive explanations of the experimental settings both in the text as in the very succinct figure legends. At times, It involved quite an effort to uncover the exact experimental details, and for some panels I did not manage to grasp the full context. The figures are also very information dense. Perhaps it would help to split some panels further into sub-panels and possibly move certain -seemingly- redundant analyses to supplemental.

2. One big outstanding question that is not addressed in this manuscript is the role of Lamin A/C in the processes attributed to Lap2alpha in this manuscript. Lamin A/C is considered an important partner of Lap2Alpha and much previous literature points towards an important role for Lamin A/C in DNA damage repair. Is the role for lamin A/C exerted via the same mechanisms? This can be quite easily addressed with a few additional experiments. E.g. in Lamin A/C -/- MEFs similar to those performed with Lap2alpha.

Figure-by-figure comments

Figure 1a.

What are the other proteins that are not annotated in this figure? A list is missing with all significant hits from the proteomics experiment. This is essential to better value the quality of the experiment. Furthermore, I wonder whether e.g. Lamin A/C is one of the interaction partners of RPA and/or other LEM-domain proteins.

I am not a proteomics expert, but to my knowledge the best way to represent such data is in a volcano plot. Again, a volcano plot would help better interpret and assign importance to the most significant and enriched interaction partners of RPA.

Figure 1d.

Here it is not clear to me what the black line represents. In addition, it is not clear with which RPA protein (1, 2 or 3) the binding is performed. I am guessing from the Coomassie that it is performed with all three simultaneously, but this should then be clarified further in the legend (see also my general comment above).

Figure 2

This is a clear example of a figure that would benefit from reducing the information content that is represented per figure panel (perhaps split up some panels) and in addition less succinct descriptions in the figure legends.

Figure 2d.

I am not sure I fully comprehend what is depicted here. The result with the 2RA (R86A, R88A) is clear, yet I do not know what else is included in this blot. I assume the 90-109 refers to the deletion that is shown in Figure 2c? Or is this a protein with two mutations in the domain corresponding to AA 90-109?

Also, can the authors explain why the delta 90-109 from Figure 2c almost completely abrogates binding to RPA1, yet in Figure 2d the point mutations in two AAs at positions AA86 & AA88 are shown to be responsible for the interaction. AA86 and AA88 are outside the delta 90-109 mutant that also shows loss of RPA interaction.

Figure 3a.

Not clear is whether this involves RPA1 or the complex of the three proteins.

The additive effect of the Lap2Alpha on ssDNA binding of RPA is minor. In fact, from the gel it is very hard to tell the difference. From the figure on the right, it appears to me that the mutant Lap2Apha has the same effect as the wildtype. This can be best seen from the unbound ssDNA fraction.

Figure 3 general.

To be able to fully comprehend the observed outcomes presented in figure 3, It should be better explained what the origin of the ssDNA is in figure 3a-c, compared to the ssDNAs used in the following figure panels.

Figure 3e.

I am not sure what the fundamental difference is between figures 3a and 3e. Is it exclusively the use of the three differently sized oligo's? That is my interpretation. Can the authors explain why for this experiment 0 to 10nM RPA protein is used, while for 3a the range is from 0 to 30nm? I am asking especially because there appears to be a clear difference in the percentage RPA-bound substrates between 10nM in panel 3a versus 3e. I understand from the Materials and Methods that the ssDNA used for figure 3a is 34bp in length (assuming I counted the nucleotides correctly) which should correspond nicely to the results obtained with 30bp in figure 3e (middle panel). But this is not the case at all. This should be better explained.

Figure 4

"Comparing to CDC45 depletion"

Please explain better the reasoning behind this depletion.

Fig. 4d

It should be explained why Lap2Alpha has an increased molecular weight for the last two lanes compared to the first three.

Fig. 4h

From this figure it does not appear that RPA loading is affected in the Lap2Alpha -/- cells as the DSB-repair stripe is already visible after 10 seconds. Rather, it is the accumulation of RPA that is severely affected. Is this consistent with the models by the authors?

**Reviewer 2**

This is a really nice study that further confirms the link between lamins, lamin associated proteins and DNA metabolism proteins such as RPA and Rad51. The work is pretty well done and clear and I do not have major issues with the paper.

I do have one issue that I would like the authors to rectify or remove>

The data showing that LAP enhances the DNA binding activity of RPA cannot be accurate. There is something not accurate about the concentration of RPA or the DNA in those reactions. Kindly consider repeating those experiments as noted below. As it does not add much to the paper, one option will be to leave out the DNA binding data. The protein-protein interaction data is important and convincing. So, please leave that included in the manuscript.

In the EMSA experiment, RPA binds to DNA stoichiometrically. We and other have observed and reported this behavior in many papers and even at 10 nM DNA (used in this paper) we should see 1:1 binding. The Kd for this interaction is ~2-3 nM and thus it is impossibly to state or uncover that LAP enhances the DNA binding activity of RPA.

Now, if we were to consider the data shown at face value, there clearly appears to be a concentration shift in the bound fraction in the presence of LAP. One explanation is that multiple DNA molecules are trapped in the presence of LAP. But, this might show up as a super-shifted complex. I do not have a better explanation either at the moment, but given the discrepancy in the lack of stoichiometry it might best to leave the DNA binding data out of this paper.

**Authors’ response**

Response to reviewers’ comments

Reviewer #1:

*In this manuscript Bao et al describe a novel role for Lap2alpha in loading RPA to ssDNA. Lap2alpha is convincingly shown to contribute to RPA-mediated signaling upon replication stress and DNA damage. Furthermore, the authors show that signaling is mediated through ATR and loading of RPA involves PARP1. This work represents a heroic biochemical effort that convincingly links Lap2alpha to DNA repair mechanisms via RPA. A role for Lap2alpha in DNA repair has been suggested previously, but to my knowledge, has not been shown so convincingly through a direct link as presented in this manuscript. I do wonder though; how suitable this work is for the audience of Genome Biology. This manuscript does not include any genomics.*

Author: We thank the reviewer for the time and effort in reviewing our manuscript and highly appreciate the very constructive comments on our story. We have re-organized the manuscript and performed additional experiments to address the reviewer’s concerns, as detailed below. This work provides “insights into molecular, cellular and organismal biology” thus meets the aims and scope of *Genome Biology*.

General comments

1. I qualified this work as a heroic effort, yet this also makes it difficult to digest for some parts of the text/figures. Some more specific comments will follow below, yet in general the manuscript could benefit from more extensive explanations of the experimental settings both in the text as in the very succinct figure legends. At times, It involved quite an effort to uncover the exact experimental details, and for some panels I did not manage to grasp the full context. The figures are also very information dense. Perhaps it would help to split some panels further into sub-panels and possibly move certain -seemingly- redundant analyses to supplemental.

Authors: We thank the reviewer’s comments and suggestions. We have provided more extensive explanations of the experimental settings both in the revised manuscript text and the corresponding figure legends. The correction could be found in a track-change style of the revised manuscript.

According to the reviewer’s suggestions, we have split the previous Figure 2C into new figures as Figure 2C, 2D, 2E, 2F and 2G. Similarly, the previous Figure 3E has been divided into new figures and shown as Figure 3E, 3F and 3G.

In addition, the previous Figure 3F, 3G, and 3H have been moved into the Supplemental Figure 1 and shown as Figure S1D, S1E, and S1F, respectively. The previous Figure 4C, 4D, and 4G have been move into the Supplemental Figure 2 and shown as Figure S2B, S2D, and S2G, respectively. The previous Figure 6B and 6E have been move into the Supplemental Figure 3 and shown as Figure S3A and S3C, respectively. The previous Figure 7A, 7B, and 7C have been move into the Supplemental Figure 4 and shown as Figure S4A, S4B, and S4H-S4I, respectively. Accordingly, the following figures have been renumbered.

2. One big outstanding question that is not addressed in this manuscript is the role of Lamin A/C in the processes attributed to Lap2alpha in this manuscript. Lamin A/C is considered an important partner of Lap2Alpha and much previous literature points towards an important role for Lamin A/C in DNA damage repair. Is the role for lamin A/C exerted via the same mechanisms? This can be quite easily addressed with a few additional experiments. E.g. in Lamin A/C -/- MEFs similar to those performed with Lap2alpha.

Authors: We appreciate the reviewer’s suggestions and insightful thoughts. The experiments suggested by the reviewer have been performed. We found, in *Lmna*−− MEFs, no discernable change of the GFP-RPA1 laser stripe or RPA2 foci was detected (Figure S2L and S2M). To consolidate this observation, we generated Lamin A/C knockout U2OS cells with CRISPR/Cas9 technology, and similar observations were obtained when examining the foci formation of RPA1 and RPA2 (Figure S2N). In addition, we showed, unlike LAP2 Lamin A/C could not be precipitated by RPA1 (Figure S2O). These results suggest that Lamin A/C is not involved in RPA loading at damaged chromatin and LAP2α functions independently of Lamin A/C in this process. The description of these results has been incorporated into the revised manuscript text (at the end of page 18 in the track change format).

Figure-by-figure comments

1. Figure 1a.

What are the other proteins that are not annotated in this figure? A list is missing with all significant hits from the proteomics experiment. This is essential to better value the quality of the experiment. Furthermore, I wonder whether e.g. Lamin A/C is one of the interaction partners of RPA and/or other LEM-domain proteins.

Authors: The list of proteins that are associated with RPA1 has been provided in Table S1. Indeed, Lamin A/C is one of the potential interaction partners of RPA1, but it is only a weak candidate (Table S1). Co-immunoprecipitation experiments with DNase-treated cellular extracts showed that Lamin A/C could not be precipitated by RPA1 (Figure S2O). Mammalian genomes contain seven individual genes that encode LEM-domain proteins: LAP2 (also known as TMPO), Emerin, MAN1, LEM2, LEMD1, Ankle1 and Ankle2 [1, 2]. Among these LEM-domain proteins, only LAP2α was identified in RPA1-containing protein complex.

I am not a proteomics expert, but to my knowledge the best way to represent such data is in a volcano plot. Again, a volcano plot would help better interpret and assign importance to the most significant and enriched interaction partners of RPA.

Authors: We thank the reviewer’s suggestion. The volcano plot has been provided in the revision and shown as Figure 1B. The 11 top candidates (fold change > 15 and *P* value < 0.01) are indicated. According to the information from The Human Protein Atlas (https://www.proteinatlas.org) and Uniprot (https://www.uniprot.org) databases, BAG3, EEF1A1P5, EIF2S3, FARSA, and SPAG5 mainly localize in the cytoplasm of cells. RUVBL1 is an abundant nuclear protein and it possesses single-stranded DNA-stimulated ATPase and ATP-dependent DNA helicase activity [3]. NAP1L1 is a histone chaperone that plays a role in the nuclear import of H2A-H2B and nucleosome assembly [4, 5], but its enrichment is lower than LAP2α. Thus, we did not focus on these proteins. We are currently working on RUVBL1 and NAP1L1 to test that whether RUVBL1 and NAP1L1 could cooperate with RPA to regulate DNA replication or DNA damage response.

2. Figure 1d.

Here it is not clear to me what the black line represents. In addition, it is not clear with which RPA protein (1, 2 or 3) the binding is performed. I am guessing from the Coomassie that it is performed with all three simultaneously, but this should then be clarified further in the legend (see also my general comment above).

Authors: The black line indicates fitted curves and the color traces represent raw data. The RPA complex including RPA1, RPA2, and RPA3 was co-purified (as shown in the “RPA purification” section in Supplemental Methods) and used in the BLI assay (now shown as Figure 1E in the revision). The above information has been provided in the figure legend. In addition, we have replaced the wrong spelling “kof” with “koff” in this figure.

3. Figure 2

This is a clear example of a figure that would benefit from reducing the information content that is represented per figure panel (perhaps split up some panels) and in addition less succinct descriptions in the figure legends.

Authors: According to the reviewer’s suggestion, Figure 2C has been split and shown as Figure 2C, 2D, 2E, 2F, and 2G. More detailed descriptions of these figures have been provided in the revised manuscript text (paragraph 1 and paragraph 2 on page 10 of the track change format) and the corresponding figure legends.

4. Figure 2d.

I am not sure I fully comprehend what is depicted here. The result with the 2RA (R86A, R88A) is clear, yet I do not know what else is included in this blot. I assume the 90-109 refers to the deletion that is shown in Figure 2c? Or is this a protein with two mutations in the domain corresponding to AA 90-109?

Authors: We apologize for the unclear description of this figure (now shown as Figure 2H in the revision). RK>AA (90-109) means that all of the arginine and lysine residues from amino acid 90 to 109 of LAP2α are simultaneously replaced by alanine, and so forth. The information has been added to the figure legend.

Also, can the authors explain why the delta 90-109 from Figure 2c almost completely abrogates binding to RPA1, yet in Figure 2d the point mutations in two AAs at positions AA86 & AA88 are shown to be responsible for the interaction. AA86 and AA88 are outside the delta 90-109 mutant that also shows loss of RPA interaction.

Authors: The results in the new Figure 2F with truncation mutants of LAP2α indicated that the region spanning from amino acid 76 to 89 is required for the interaction of LAP2α with RPA1, and co-immunoprecipitation assays with deletion mutants of LAP2α in the new Figure 2G suggested that the fragment 90-109 is also essential in LAP2α-RPA1 binding. In the new Figure 2H (previous shown as Figure 2D), mutational analysis showed that only R86 and R88 substitution could impair the interaction, whereas all of the point mutations in 90-109 appeared to have little effect on RPA interaction. These results suggest that the fragment 76-89 in the linker region of LAP2α likely binds to RPA1 directly, while 90-109 may contribute to the interaction indirectly. We have re-described these results in the revised manuscript text (paragraph 1 and paragraph 2 on page 10 of the track change format).

5. Figure 3a.

Not clear is whether this involves RPA1 or the complex of the three proteins.

Authors: The RPA complex of the three proteins (RPA1, RPA2 and RPA3) was used in all of the EMSA assays of Figure 3 and Figure S1C. So were the BRET assays (now shown as Figure S1D, S1E, and S1F). To clearly present our data, we have added the information at the end of the figure legend of Figure 3 and Figure S1. The additive effect of the Lap2Alpha on ssDNA binding of RPA is minor. In fact, from the gel it is very hard to tell the difference. From the figure on the right, it appears to me that the mutant Lap2Apha has the same effect as the wildtype. This can be best seen from the unbound ssDNA fraction.

Authors: We appreciate the reviewer’s criticism. The gel blots related to the previous Figure 3A at different exposure times were provided (Data to the reviewer: Figure R1A). From the longer exposure blot, we can see the RPA bound ssDNA in the 5th lane (RPA 7.5 nM, LAP2α present) is stronger than that in the 2nd lane (RPA only). In the shorter exposure blot (the same as previous Figure 3A), the RPA bound ssDNA fraction in the presence of LAP2α is stronger than that in the absence of LAP2α (lane 6 vs lane 3, and lane 7 vs lane 4), and the free ssDNA is less when LAP2α was preincubated with RPA (lane 5 vs lane 2, lane 6 vs lane 3, and lane 7 vs lane 4). The most evident difference of free ssDNA fraction could be seen between lane 6 and lane 3.

**Figure R1A.** The longer (left panel) and shorter (right panel) exposure blots related to EMSA results in previous Figure 3A.

Similarly, we also provided the gel blots related to the previous Figure 3B at different exposure times (Data to the reviewer: Figure R1B). From the shorter exposure blot, we can see the unbound ssDNA in LAP2α/2RE lane is discernably stronger than that in the LAP2α/wt lane, albeit it is less than that in the LAP2α absent lane. In addition, the RPA bound ssDNA fraction in the presence of LAP2α/wt is much stronger than that in the presence of LAP2α/2RE.

**Figure R1B.** The longer (left panel) and shorter (right panel) exposure blots related to EMSA results in previous Figure 3B.

In the revision, we have optimized the experimental procedures and re-performed the EMSA assays in Figure 3A, 3B and 3C. The amount of ssDNA was 10 nM in the final reaction system of these EMSA assays. In the new Figure 3A, the concentration of RPA (0, 5, 10, and 15 nM) and LAP2α (15 nM ) was proportionally adjusted according to the titration assays in the middle panel of previous Figure 3E (now shown as Figure 3F). From the new data, we can see that the alterations of RPA bound ssDNA and free ssDNA are largely in the opposite direction and, at maximum, almost 3 folds increase of RPA-ssDNA binding was detected when LAP2α was present. For EMSA assays in the new Figure 3B, 10 nM LAP2α variants and 10 nM RPA (proportionally adjusted according to the titration assays in Figure S1C) were used. Consistent with the results using 5’ Cy3 labelled dT30 ssDNA in Figure S1C, LAP2α/2RE failed to alter the RPA-ssDNA formation. The same amounts of LAP2α (10 nM) and RPA (10 nM) were used in the new Figure 3C. The previous data in Figure 3A, 3B and 3C have been replaced with the new ones.

Considering that RPA alone binds to ssDNA with sub-nanomolar affinity, it is reasonable to see that the potential chaperone proteins or assistant factors only mildly increase RPA loading efficiency. In agreement with our observations, the yeast RPA chaperone Rtt105 enhances RPA-ssDNA binding to a similar degree (3-4 folds), as shown in Figure 4C of Li’s study [6]. To precisely present our data, we have replaced the word “dramatically” with “significantly” in the revised manuscript text (the middle paragraph on page 11 of track change format).

6. Figure 3 general.

To be able to fully comprehend the observed outcomes presented in figure 3, It should be better explained what the origin of the ssDNA is in figure 3a-c, compared to the ssDNAs used in the following figure panels.

Authors: In Figure 3A, 3B and 3C, 5’ biotin-labelled 34-nucleotide (nt) ssDNA with randomized sequence [7], was used to perform EMSA assays. The ssDNA used in Figure 3D, the previous Figure 3E (now shown as Figure 3E, 3F, and 3G), and the previous Figure 3G-3H (now shown as Figure S1E-S1F) consists of oligodeoxythymidine (oligo(dT)) with different lengths. These oligo(dT)s are homogenous in composition and make the results of EMSA assays (Figure 3A-3C) or competition binding assays (Figure 3D) comparable. The information has been provided in the corresponding figure legend of the revised manuscript.

In addition, we found the unbound ssDNA fraction did not change markedly in the dT30 panel of previous Figure 3E (now shown as Figure 3F), although the RPA bound fraction increased evidently in the presence of LAP2α. We re-examined our original EMSA results, and have replaced the previously submitted data (Replicate 1) with the middle image (Replicate 2) from biological triplicate results we performed before the first submission (Data to the reviewer: Figure R1C). The new data that more intuitively matches with the binding curve has been provided in Figure 3F of the revision.

**Figure R1C.** Biological triplicate EMSA results (performed before the first submission) related to previous Figure 3E. Replicate 1 (the middle panel of previous Figure 3E) has been replaced by Replicate 2 and shown as Figure 3F in the revision.

7. Figure 3e.

I am not sure what the fundamental difference is between figures 3a and 3e. Is it exclusively the use of the three differently sized oligo’s? That is my interpretation. Can the authors explain why for this experiment 0 to 10nM RPA protein is used, while for 3a the range is from 0 to 30nm? I am asking especially because there appears to be a clear difference in the percentage RPA-bound substrates between 10nM in panel 3a versus 3e. I understand from the Materials and Methods that the ssDNA used for figure 3a is 34bp in length (assuming I counted the nucleotides correctly) which should correspond nicely to the results obtained with 30bp in figure 3e (middle panel). But this is not the case at all. This should be better explained.

Authors: When we initiated the project, the biotin-labelled 34-nt ssDNA was used in Figure 3A-3C, and ssDNA signals on the gel were transferred to a nylon membrane followed by blotting with the chemiluminescent EMSA kit. To quantitatively assess the effect of ssDNA length in LAP2α-promoted RPA loading and further understand how LAP2α affects the formation of ssDNA-RPA complex, we took different lengths of 5’ Cy3-labelled oligo(dT) including dT17, dT30 and dT70 to perform the fluorescence-based EMSA assays. These oligo(dT)s are homogenous in composition and make the results of EMSA assays comparable. In the EMSA assays of previous Figure 3E (now shown as Figure 3E, 3F, and 3G), fluorescent ssDNA signals on the gel were directly detected with an Alliance Q9 imager, which ensures a more fast and accurate examination.

When performing chemiluminescent based EMSA assays (Figure 3A-3C), we first titrated the amount of ssDNA, and found that 10 nM ssDNA gave more bright and consistent binding results, thereby we chose 10 nM ssDNA in these EMSA assays. Accordingly, we used more RPA and LAP2α to scale up the reaction system of EMSA assays with biotin-labelled 34-nt ssDNA, albeit not exactly proportional to the EMSA assays with Cy3-labelled oligo(dT). In the previous Figure 3E, we chose 5 nM ssDNA, since this concentration produced equal results as 10 nM ssDNA in the binding assay. When comparing the RPA binding curve (34 nt vs dT30) at the same molar ratio of RPA:ssDNA from previous Figure 3A and the middle panel of previous Figure 3E (now shown as Figure 3F), we found the binding efficiency of 10 nM RPA and 10 nM ssDNA in previous Figure 3A (20%) was similar to that of 5 nM RPA and 5 nM ssDNA in the middle panel of previous Figure 3E (20%), and the same is true when the amount of RPA was increased to 1.5 times or 2 times in these figures (40% vs 40%; 50% vs 60%). Meanwhile, at 1:1, 1.5:1, or 2:1 molar ratio of RPA:ssDNA, the stimulation effect induced by LAP2α in EMSA assays with biotin-labelled 34-nt ssDNA from the previous Figure 3A was comparable to that using Cy3-labelled dT30 in the middle panel of previous Figure 3E (2-3 folds). The concentration of ssDNA, RPA, and LAP2α was provided in the corresponding figure legends in our previously submitted manuscript. Thereby, the results in these EMSA assays with different forms of ssDNA are largely consistent (Figure R1D).

Figure R1D. The RPA binding curves in previous Figure 3A and the middle panel of previous Figure 3E (now shown as Figure 3F).

To address the reviewer’s concern in “question#2 of figure 3a” and to more clearly present our data, we have optimized the experimental procedures and re-performed the EMSA assays in the previous Figure 3A, 3B and 3C. The amounts of ssDNA and proteins in the new data provided in the revision, are proportional to that of the EMSA assays with Cy3-labelled oligo(dT).

8. Figure 4

"Comparing to CDC45 depletion"

Please explain better the reasoning behind this depletion.

Authors: As described in the “BACKGROUND” section, CDC45 has been reported to directly load RPA on the emerging nascent ssDNA at replication fork [7]. Thereby, we took it as a control in our iPOND assays. A brief description on this has been provided in the revised manuscript text (paragraph 1 on page 15 of the track change format).

9. Fig. 4d

It should be explained why Lap2Alpha has an increased molecular weight for the last two lanes compared to the first three.

Authors: LAP2α or its point mutant was carried by lentiviral vector encoding a 3 X FLAG tag in front of *LAP2*α CDS (Plasmid section of the Supplemental Methods). The 3 X FLAG and its following linker will produce approximate 30 amino acids. Theoretically, the FLAG-tagged LAP2α variants will express protein products with an increased molecular weight of 3-4 kDa comparing to the endogenous LAP2α. This could be the reason that LAP2α has an increased molecular weight for the last two lanes compared to the first three in the previous Figure 4D (now shown as Figure S2D). The explanation has been incorporated into the figure legend in Figure S2D of the revision. Yet, this is not applicable to FLAG-tagged PARP1 as shown in the previous Figure S4I (now shown as Figure S4M). PARP1 is a huge protein and its molecular weight (larger than 135 kD) is almost twofold comparing to that of LAP2α (75-85 kD). Thus, addition of a 3 X FLAG tag with 3-4 kDa should not induce an apparent retardation of PARP1 on the gel.

10. Fig. 4h

From this figure it does not appear that RPA loading is affected in the Lap2Alpha -/- cells as the DSB-repair stripe is already visible after 10 seconds. Rather, it is the accumulation of RPA that is severely affected. Is this consistent with the models by the authors?

Authors: We thank the reviewer for this comment. The DSB-repair stripe of RPA in the *Lap2*α−− cells is indeed visible after 10 seconds of laser micro-IR, but it is still weaker than that in *Lap2*α++ cells. The statistical results of the early time points have been provided in Figure S2H. The increasing difference of RPA intensity at laser stripes from early to late time points in wild type versus knockout cells (Figure S2H), suggests that there could be a differential extent of requirement of LAP2α for RPA loading during a progressive DNA end resection-coupled RPA deposition process. When DNA damage occurs, the ssDNA is shorter and only a small number of RPA is required to coat the ssDNA. Possibly, RPA does not rely too much on the assistance from chaperone factors at this time point. Once the break ends are processed into longer stretch of ssDNA, a growing number of RPA is needed to meet the demand of extensive ssDNA and RPA chaperones are likely more required to timely raise the efficiency of RPA loading. Additionally, the alterations and dynamic changes of damaged chromatin environment may also contribute to the increased efficiency of LAP2α-promoted RPA loading. These are the possible reasons that, in *Lap2*α−− cells, we observed a mild decrease of RPA binding after 10 seconds of laser micro-IR and a severe defect of RPA accumulation in the following time points.

As the reviewer pointed, LAP2α may control RPA accumulation through mechanisms other than promoting its loading. If LAP2α is able to promote RPA retention or inhibit its removal, we should see forced expression of LAP2α in LAP2α-deficient cells will suppress RPA clearance and, to certain extent, may result in an impairment of RAD51 foci formation, given that RPA needs to be replaced by the recombinase RAD51 [8, 9]. However, the foci formation of RAD51 was not evidently changed in these cells (previous Figure 6D, now shown as Figure 6C). Thus, it seems that LAP2α may not act to inhibit RPA removal or promote its retention. The intrinsic ssDNA binding activity of RPA excludes the possibility that LAP2α functions to directly recruit RPA. The term “RPA recruitment” in the manuscript text is used to reflect the binding affinity of RPA and ssDNA.

Although we can’t provide direct evidence for the above arguments, our study suggests that the effect of LAP2α on RPA accumulation could be largely attributed to its role in RPA loading. This is supported by the following observations: 1) LAP2α actively promotes the formation of the ssDNA-RPA complex *in vitro* and LAP2α-RPA interaction plays an essential role in this process (Figure 3 and Figure S1); 2) LAP2α/wt could significantly restore LAP2α depletion-associated defects of RPA binding to ssDNA and recruitment to DNA damage sites, whereas LAP2α/2RE that could not stimulate RPA loading failed to do so (Figure 4). In addition, we showed LAP2α loss had minor effect on BrdU foci or laser stripe formation under non-denaturing conditions (previous Figure 6E and S3B, now shown as Figure S3C and Figure S3D), ruling out the possibility that LAP2α-promoted RPA deposition on DNA damage sites is a secondary effect of enhanced DNA end resection.

To address the reviewer’s concern and to precisely present our data, we have discussed this issue in the revised manuscript text (paragraph 2 on page 17 in the RESULTS section and paragraph 1 on page 31 in the DISCUSSION section of the track change format).

RE: MS#GBIO-D-21-00876

Title: LAP2α Preserves Genome Integrity through Actively Loading RPA onto Single-Stranded DNA

Response to reviewers’ comments

Reviewer #2:

This is a really nice study that further confirms the link between lamins, lamin associated proteins and DNA metabolism proteins such as RPA and Rad51. The work is pretty well done and clear and I do not have major issues with the paper.

Author: We thank the reviewer for the time and efforts in reviewing our manuscript and highly appreciate the very positive and constructive comments.

I do have one issue that I would like the authors to rectify or remove>

The data showing that LAP enhances the DNA binding activity of RPA cannot be accurate. There is something not accurate about the concentration of RPA or the DNA in those reactions. Kindly consider repeating those experiments as noted below. As it does not add much to the paper, one option will be to leave out the DNA binding data. The protein-protein interaction data is important and convincing. So, please leave that included in the manuscript.

In the EMSA experiment, RPA binds to DNA stoichiometrically. We and other have observed and reported this behavior in many papers and even at 10 nM DNA (used in this paper) we should see 1:1 binding. The Kd for this interaction is ~2-3 nM and thus it is impossibly to state or uncover that LAP enhances the DNA binding activity of RPA.

Now, if we were to consider the data shown at face value, there clearly appears to be a concentration shift in the bound fraction in the presence of LAP. One explanation is that multiple DNA molecules are trapped in the presence of LAP. But, this might show up as a super-shifted complex. I do not have a better explanation either at the moment, but given the discrepancy in the lack of stoichiometry it might best to leave the DNA binding data out of this paper.

Authors: We thank the reviewer’s comments and criticisms. According to the editor’s suggestion, we keep the EMSA results in the revised manuscript. For the stoichiometry of RPA-ssDNA binding, the increasing concentration of RPA over ssDNA (30 nt) alters the binding mode from 30 nt to 8-10 nt [10-12]. In specific, the 1:1 stoichiometry suggests that RPA binds ssDNA in the stable 30 nt binding mode, while in the 2:1 molar ratio RPA interacts in the 13-22 nt binding mode and finally formation of 3:1 complexes would be in accordance with a less stable 8-10 nt binding mode [10, 13]. Only when the ratio of RPA to ssDNA is over 5 times or more, the stoichiometry or binding mode for 30 nt ssDNA starts to change [10, 13]. Since the largest RPA:ssDNA (34 nt or dT30) ratio is 3:1 in our study (previous Figure 3A, 3B and 3C), we believe that the binding mode change should not happen, and the stoichiometry will stay at 1:1.

Although we believe the stoichiometry of RPA-ssDNA (34-nt or dT30) binding is 1:1 in our study, we repeatedly found that only 20%-25% of dT30 or 34-nt ssDNA is bound to RPA when the molecular ratio of RPA:ssDNA is 1:1 (Figure 3A, 3B, 3C, 3F and Figure S1C), and more ssDNA is occupied by RPA when the ratio increases (Figure 3A and 3F). When checking the literatures, we found Pokhrel’s study [14] and Yates’s study [15] have reported a near 100% of RPA-ssDNA binding from the EMSA and BRET assays, respectively. However, our observation is largely consistent with previous reports from Dr. Marc S. Wold whose lab initially established the methods of RPA purification and the EMSA experiments (as shown in Figure 6A of the referred paper) [16], Dr. Qing Li whose lab identified Rtt105 is a yeast RPA chaperone (as shown in Figure 4B and 4C of the referred paper) [6], Dr. Frank Grosse whose lab characterized CDC45 as an assistant factor for RPA loading during DNA replication (as shown in Figure 2A of the referred paper) [7], Miyake’s study (as shown in Figure 4D of the referred paper) [17], Salas’s study (as shown in Figure 1A and 1B of the referred paper) [10], Shuck’s study (as shown in Figure 1A of the referred paper) [18], Salas’s study (as shown in Figure 2 of the referred paper) [13], and Martínez-Jiménez’s study (as shown in Figure 3B of the referred paper) [19]. In these studies, approximately 10%-50% ssDNA is bound to RPA when RPA:ssDNA ratio is 1:1 [6, 13, 18], and a discernable fraction of free ssDNA remained even when the ratio is greater than 1:1 for the other cases [7, 10, 16, 17, 19]. Possibly, the reaction condition including buffer composition, incubation time and the temperature may affect the binding efficiency. Indeed, Salas’s study (as shown in Figure 2 of the referred paper) [13] showed that RPA-ssDNA complex forms with different efficiencies in the presence of 50 mM NaCl or 50 mM KCl. It could be also due to the formed RPA-ssDNA products may be dynamically replaced by the free RPA in solution [20], or they may inhibit the undergoing reaction so that an equilibrium of the binding is achieved before most or all ssDNA is occupied by RPA.

Our study together with the Rtt105 and CDC45 work suggests that in a favorable, yet not optimal, condition of RPA-ssDNA binding, RPA chaperones or assistant factors could facilitate the shift of the binding equilibrium and promote RPA loading onto ssDNA. Considering that LAP2α does not bind ssDNA (Figure 3A and 3B) and LAP2α mutant (LAP2α/2RE) that could not bind RPA is not able to promote the ssDNA-RPA complex formation (Figure 3B and Figure S1C), we propose that the stimulating effect in our EMSA assays is attributed to LAP2α itself, but not an artificial effect or some unknown systematic bias. Hopefully, the reviewer will agree with our response on this issue.

According to the reviewer#1’s suggestion, we have optimized and re-performed the EMSA assays with the maximum RPA:ssDNA ratio as 2:1 in Figure 3A, 3B, and 3C and new data have been provided, and we have replaced the middle panel of previous Figure 3E with new data from biological triplicate results we performed before the first submission (Figure R1C and the new Figure 3F).

**References**

1. Brachner A, Foisner R: **Lamina-associated polypeptide (LAP)2alpha and other LEM proteins in cancer biology.** *Adv Exp Med Biol* 2014, **773:**143-163.

2. Brachner A, Foisner R: **Evolvement of LEM proteins as chromatin tethers at the nuclear periphery.** *Biochem Soc Trans* 2011, **39:**1735-1741.

3. Puri T, Wendler P, Sigala B, Saibil H, Tsaneva IR: **Dodecameric structure and ATPase activity of the human TIP48/TIP49 complex.** *J Mol Biol* 2007, **366:**179-192.

4. Okuwaki M, Kato K, Nagata K: **Functional characterization of human nucleosome assembly protein 1-like proteins as histone chaperones.** *Genes Cells* 2010, **15:**13-27.

5. Ohtomo H, Akashi S, Moriwaki Y, Okuwaki M, Osakabe A, Nagata K, Kurumizaka H, Nishimura Y: **C-terminal acidic domain of histone chaperone human NAP1 is an efficient binding assistant for histone H2A-H2B, but not H3-H4.** *Genes Cells* 2016, **21:**252-263.

6. Li S, Xu Z, Xu J, Zuo L, Yu C, Zheng P, Gan H, Wang X, Li L, Sharma S, et al: **Rtt105 functions as a chaperone for replication protein A to preserve genome stability.** *EMBO J* 2018, **37**.

7. Szambowska A, Tessmer I, Prus P, Schlott B, Pospiech H, Grosse F: **Cdc45-induced loading of human RPA onto single-stranded DNA.** *Nucleic Acids Res* 2017, **45:**3217-3230.

8. Heyer WD, Ehmsen KT, Liu J: **Regulation of homologous recombination in eukaryotes.** *Annu Rev Genet* 2010, **44:**113-139.

9. Huang TH, Fowler F, Chen CC, Shen ZJ, Sleckman B, Tyler JK: **The Histone Chaperones ASF1 and CAF-1 Promote MMS22L-TONSL-Mediated Rad51 Loading onto ssDNA during Homologous Recombination in Human Cells.** *Mol Cell* 2018, **69:**879-892 e875.

10. Salas TR, Petruseva I, Lavrik O, Saintome C: **Evidence for direct contact between the RPA3 subunit of the human replication protein A and single-stranded DNA.** *Nucleic Acids Res* 2009, **37:**38-46.

11. Fan J, Pavletich NP: **Structure and conformational change of a replication protein A heterotrimer bound to ssDNA.** *Genes Dev* 2012, **26:**2337-2347.

12. Bochkareva E, Korolev S, Lees-Miller SP, Bochkarev A: **Structure of the RPA trimerization core and its role in the multistep DNA-binding mechanism of RPA.** *EMBO J* 2002, **21:**1855-1863.

13. Salas TR, Petruseva I, Lavrik O, Bourdoncle A, Mergny JL, Favre A, Saintome C: **Human replication protein A unfolds telomeric G-quadruplexes.** *Nucleic Acids Res* 2006, **34:**4857-4865.

14. Pokhrel N, Origanti S, Davenport EP, Gandhi D, Kaniecki K, Mehl RA, Greene EC, Dockendorff C, Antony E: **Monitoring Replication Protein A (RPA) dynamics in homologous recombination through site-specific incorporation of non-canonical amino acids.** *Nucleic Acids Res* 2017, **45:**9413-9426.

15. Yates LA, Aramayo RJ, Pokhrel N, Caldwell CC, Kaplan JA, Perera RL, Spies M, Antony E, Zhang X: **A structural and dynamic model for the assembly of Replication Protein A on single-stranded DNA.** *Nat Commun* 2018, **9:**5447.

16. Henricksen LA, Umbricht CB, Wold MS: **Recombinant replication protein A: expression, complex formation, and functional characterization.** *J Biol Chem* 1994, **269:**11121-11132.

17. Miyake Y, Nakamura M, Nabetani A, Shimamura S, Tamura M, Yonehara S, Saito M, Ishikawa F: **RPA-like mammalian Ctc1-Stn1-Ten1 complex binds to single-stranded DNA and protects telomeres independently of the Pot1 pathway.** *Mol Cell* 2009, **36:**193-206.

18. Shuck SC, Turchi JJ: **Targeted inhibition of Replication Protein A reveals cytotoxic activity, synergy with chemotherapeutic DNA-damaging agents, and insight into cellular function.** *Cancer Res* 2010, **70:**3189-3198.

19. Martinez-Jimenez MI, Lahera A, Blanco L: **Human PrimPol activity is enhanced by RPA.** *Sci Rep* 2017, **7:**783.

20. Ma CJ, Gibb B, Kwon Y, Sung P, Greene EC: **Protein dynamics of human RPA and RAD51 on ssDNA during assembly and disassembly of the RAD51 filament.** *Nucleic Acids Res* 2017, **45:**749-761.

**2^nd^ round**

**Reviewer 1**

I have no further comments. The authors did a great job in adressing my concerns.

**Reviewer 2**

The authors have done a good job of revising the manuscript and I’m OK with most of the changes. I will sign off on the paper only if the authors either:

1. remove the section, data, and all statements about LAP2a enhancing the DNA binding activity of RPA. In their rebuttal, the authors cite the paper from the Li group that shows Rtt105 enhancing the DNA binding activity of RPA. This again is simply erroneous conclusion from the Li paper. Please see the data shown below from our unpublished work. The most obvious explanation is that these RPA protein preps are not fully active. There should be no scenario with RPA where you have only 30% of the protein binding to DNA as explained by the authors. Some of the explanations they provide are also bizarre as those scenarios will produce distinct super-shifted complexes which are not observed in their experiments.

We have a paper coming out soon which will address this [a figure was provided by the reviewer but has been removed at their request before publication of this report]

2. My primary concern is the propagation of the idea that these proteins (including Rtt105) being stated as enhancers of RPA’s DNA binding activity *in vitro*. We are striving very hard to make sure folks produce proper data to support such a conclusion. The Li data is incorrect (confusing at best) and here we have the authors already citing it as evidence. Please work with us to put an end to this!
3. Now, stating that case, even if one were to qualitatively assess the data in Figure 3, there is a clear shift in the amount of DNA bound when Lap2a is present in the reaction. An alternate explanation is the presence of a proper DNA binding site(s) in LAP2a, that become exposed (or activated) when in complex with RPA. This is about the only reasonable explanation I have for the increase in binding activity. More, higher resolution DNA binding experiments will have to be carried out to better understand this behavior and we are happy to extend our expertise in in depth RPA-DNA interactions to the authors if they so desire to resolve the differences as a follow up study.

As such, if the authors are willing to edit the manuscript to address the concerns above, then I am OK with acceptance for publication.
